# Selective N-terminal functionalization of native peptides and proteins†
†Electronic supplementary information (ESI) available: Experimental details. See DOI: 10.1039/c6sc04744k



**DOI:** 10.1039/c6sc04744k

**Published:** 2017-01-09

**Authors:** Diao Chen, Maria M. Disotuar, Xiaochun Xiong, Yuanxiang Wang, Danny Hung-Chieh Chou

**Affiliations:** a Department of Biochemistry , University of Utah , 15 N. Medical Drive East 4100 , Salt Lake City , UT 84112 , USA . Email: dchou@biochem.utah.edu

## Abstract

We report an efficient, highly selective modification on the N-terminal amines of peptides and proteins using aldehyde derivatives *via* reductive alkylation. After modification of a library of unprotected peptides XYSKEASAL (X varies over 20 natural amino acids) by benzaldehyde at room temperature, pH 6.1 resulted in excellent N-terminal selectivity (α-amino/ε-amino: >99 : 1) and high reaction conversion for 19 out of the 20 peptides. Under similar conditions, highly selective N-terminal modifications were achieved with a variety of aldehydes. Furthermore, N-termini of native peptides and proteins could be selectively modified under the same conditions to introduce bioorthogonal functional groups. Using human insulin as an example, we further demonstrated that preserving the positive charge in the N-terminus using reductive alkylation instead of acylation leads to a 5-fold increase in bioactivity. In summary, our reported method provides a universal strategy for site-selective N-terminal functionalization in native peptides and proteins.

## Abstract 2

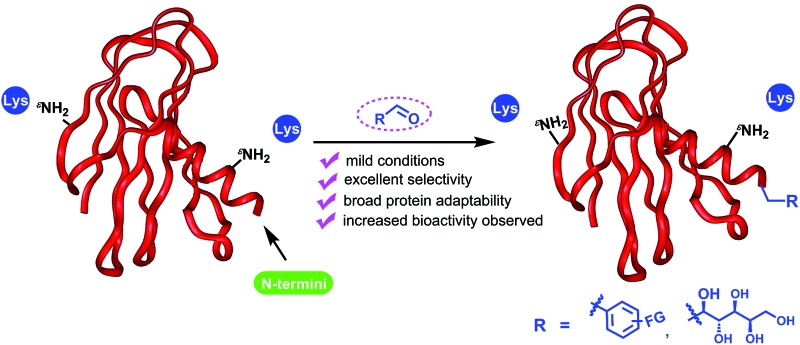
A highly site-selective modification of peptides/proteins with aldehydes or carbohydrates under mild conditions was achieved.

## Introduction

**Fig. 1:**
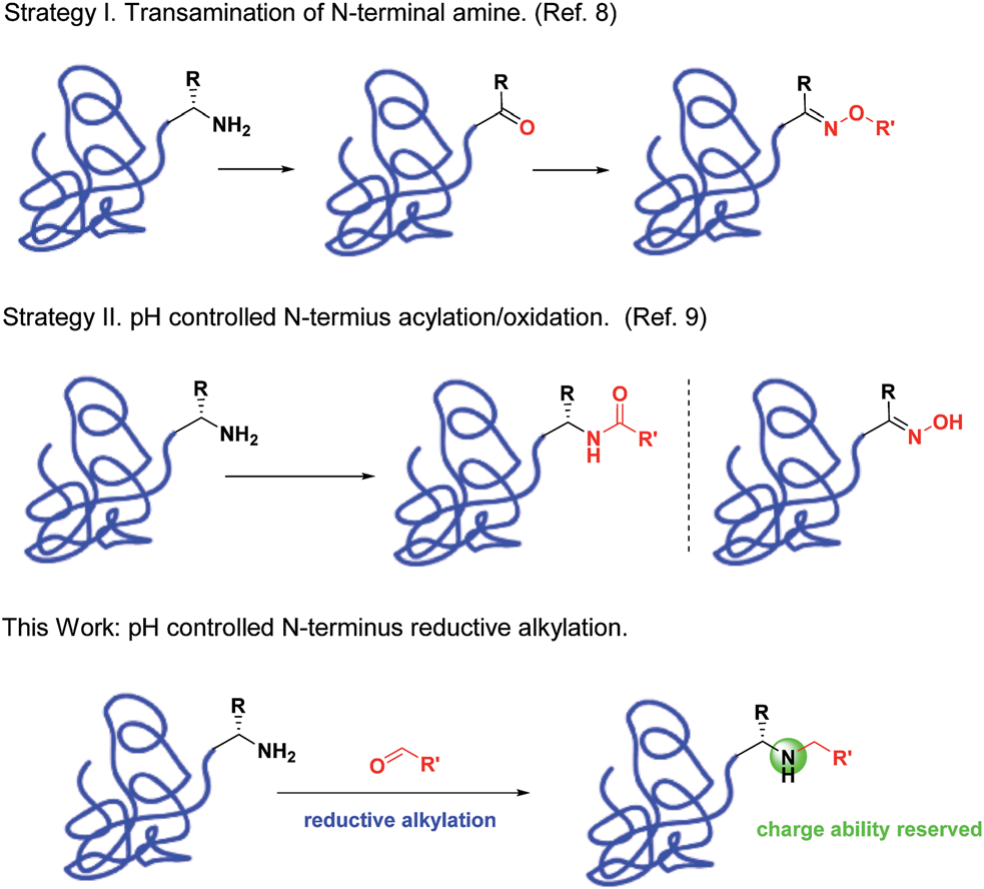
Selective modification of protein N-terminus.

Modified peptides and proteins have led to a suite of tool molecules to study biological processes and valuable therapeutics to treat human diseases.^1^ Due to the wide applications, a series of selective modification methods^2^ have been developed to modify lysines,^3^ cysteines,^1b,2f,g,4^ tyrosines,^5^ tryptophans^6^ or N-termini.^7–10^ Among these positions, the N-terminus position is an appealing target because there is generally only one such group in a single-chain peptide or protein. Chemoselective modifications on these sites can therefore result in site-specific bioconjugations. To date, a number of methods have been reported to achieve this specificity. For example, it was reported that the N-termini can be selectively conjugated with other bioorthogonal functional groups through transamination reaction.^8^ Furthermore, pH-controlled N-terminal selective acylations^9a,b^ or oxidation^9*c*^ have also been reported. Recently, Francis *et al.* demonstrated that 2-pyridinecarbaldehyde (2-PCA) can be used for site-specific modification of the N-terminus through the formation of an imidazolidinone ring.^9*d*^ All these methods represent a good list for various applications. However, more complementary methods are required to provide a comprehensive set of tools to address challenges faced by protein modifications. For instance, N-terminal transamination enables direct introduction of new functionalities for subsequent reactions;^8^ however, this is at the cost of replacing the N-terminal amines, which in certain situations can lead to loss of bioactivity. On the other hand, modifications using ketenes^9a,b^ or 2-PCA^9*d*^ preserve the nitrogen atom. However, derivatives of ketene or 2-PCA that can be used for direct introduction of bioorthogonal groups are not commercially available and require multistep synthesis. Moreover, the modified proteins with acylated N-terminus will lose the positive charge ability *in vivo*,^9a,b^ which is important for the function of proteins such as insulin.^13^ Therefore, an efficient and convenient method to introduce N-terminal functionalities, with charge ability *in vivo* reserved, in peptides and proteins in a single step would be a valuable tool for biological studies and therapeutic development. Herein, we report an efficient method of N-terminal modifications of peptides and proteins *via* reductive alkylation (Fig. 1).

It is known that the p*K*_a_ value of N-terminal amine (∼8) is lower than that of the lysine amine (∼10).^9,10^ In the literature, N-terminal selective transamination,^8^ acylation,^9a,b^ and oxidation^9*c*^ have been achieved taking advantage of this p*K*_a_ difference. Since the reductive alkylation of amine normally occurs under acidic conditions, we envisaged that the modification would selectively take place on the N-termini since the lysine ε-amine is largely pronated under this condition. Reductive alkylation preserves the N-terminal amine, which may lead to retained bioactivity, as demonstrated in previous studies.^13^ Due to the commercial availability of a variety of benzaldehyde derivatives with bioorthogonal functional groups, it is advantageous to obtain various bioconjugates using this method. Interestingly, similar strategies with limited substrates have succeeded in selective N-terminal PEGylation of peptides^11a,b^ or therapeutic proteins.^11*c*^ The corresponding PEGylated conjugates indicated improved pharmacokinetic properties with preserved bioactivity.^11^ Furthermore, reductive alkylation has been used for glycosylations on both the lysine and N-termini of the insulin molecule.^12^ Herein, we systematically investigated the reductive alkylation of peptides and proteins with aldehyde derivatives to explore their features in selective N-terminal functionalization. We demonstrated that under mild conditions, aldehyde derivatives, as well as carbohydrates, can site-specifically react with peptide and protein N-termini. Bioorthogonal functional groups, such as alkyne and ketone, could be inserted on the N-termini in a single step to enable site-specific labeling of the resulting products. Furthermore, in the case of insulin modifications, we showed that the resulting secondary amines from reductive alkylation led to a 5-fold increase in the bioactivity compared to an amide linkage. This further demonstrated the value of this method to provide complementary advantages over existing methods.

## Results and discussion

We first investigated the conditions that provide the optimal N-terminal selectivity (Table 1). Our initial study began with the unprotected model peptide GYSKEASAL (**1a**) and benzaldehyde (**2a**) in acetic acid buffer (pH 3.3) in the presence of sodium cyanoborohydride at room temperature for 6 hours. We obtained the mono-modified products (**3a** and **3a′**) in 49% conversion with a >99 : 1 N-terminal selectivity (Table 1, entry 1). We also observed 38% of the product being the di-modified peptide (both on N-terminus, **3a′′**). The di-modified product was observed likely due to the fact that the desired secondary amine product is more reactive than the starting peptide. Following this observation, a series of buffer conditions with different pH were screened to identify better mono-modified conversion and N-terminal selectivity (Table 1, entries 2–7). We observed the best outcome (78% conversion, >99 : 1 selectivity) with pH 6.1 citric acid buffer as the solvent (entry 4). This condition was chosen for further studies.

**Table 1:** Condition optimization of N-terminal alkylation^*a*^ ^,^ ^*b*^ ^,^ ^*c*^ ^,^ ^*f*^

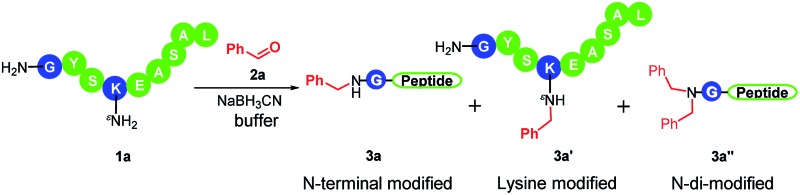					
Entry	Buffer	pH	(**3a** + **3a′**) (%)	N-Selectivity^*d*^	**3a′′** (%)
1	A	3.3	49	>99 : 1	38
2	B	4.0	58	98 : 2	30
3	A	6.0	60	95 : 5	22
4	B	6.1	78	>99 : 1	18
5	C	7.4	60	80 : 20	20
6	D	9.7	35	73 : 27	7
7	C	10.0	50	90 : 10	10
8^*e*^	B	6.1	60	>99 : 1	16

^*a*^The reaction was performed with 2.7 μmol peptide, 5.4 μmol, benzaldehyde (0.5 M in DMSO), and 13.5 μmol NaBH_3_CN in 300 μL aqueous solvent at room temperature for 6 h.

^*b*^Buffer A is 25 mmol L^–1^ acetic acid buffer; buffer B is 25 mmol L^–1^ citric acid buffer; buffer C is 25 mmol L^–1^ phosphate buffer; buffer D is 25 mmol L^–1^ borate buffer.

^*c*^The conversion was calculated by area of the corresponding peak under 280 nm UV detection of the reaction system.

^*d*^Determined by MS/MS analysis.

^*e*^1.5 equivalents benzaldehyde was used.

^*f*^See ESI Table S1 for more condition screening data.

**Scheme 1:**
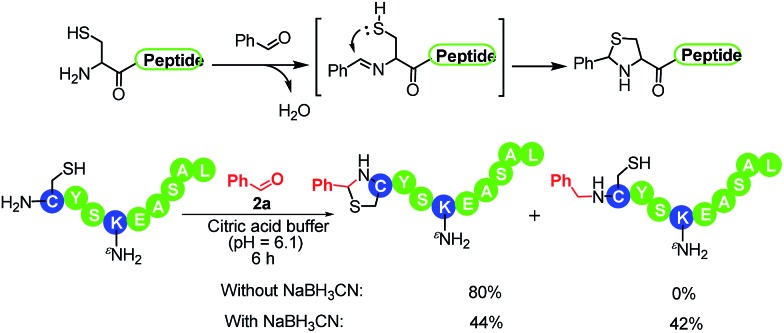
Reaction of N-terminal cysteines and benzaldehydes.

The N-terminal residue plays an important role in affecting N-terminal modifications. To demonstrate the scope of this method, we sought to systematically investigate the effects of all N-terminal residues on the reaction performance by individually reacting peptides **1a–1t** with benzaldehyde. The results are summarized in Table 2. In all the cases, the reaction was accomplished in 4–6 hours, resulting in the corresponding modified peptides in good conversion with excellent regioselectivity (**3b** to **3t**, all >99 : 1 selectivity). There was only one exception: 42% conversion was obtained in the case of an N-terminal cysteine along with another 44% conversion to the thiazolidine (entry 16). The formation of thiazolidine is generated through the intramolecular addition of the thiol to the iminium ion (Scheme 1). This observation is consistent with a recent study reported by Gao *et al.* that N-terminal cysteines can react with benzaldehyde under acidic conditions (pH 5) to form thiazolidines in 2 days.^7*e*^ We further demonstrated that the reaction was accelerated (6 hours) at pH 6.1 with a high conversion without the addition of hydride (Scheme 1). In summary, the reductive amination reaction led to excellent conversion in 19 out of 20 possible N-terminal residues. Furthermore, N-terminal methionine (most frequent N-terminal residues in proteins) gives excellent conversion (Table 2, entry 12).^14^ Considering the low-occurrence of N-terminal cysteines, this method can be applied to the majority of native peptides and proteins.

**Table 2:** Peptide scope for the N-terminal alkylation^*a*^

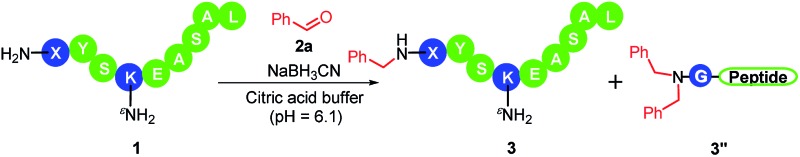					
Entry	N-Terminal amino acid	**3**	**3** + **3′** ^*b*^ (%)	N-Selectivity^*c*^ (%)	**3′′** ^*b*^ (%)
1	Glycine (G)	**3a**	78	>99 : 1	18
2	Alanine (A)	**3b**	82	>99 : 1	13
3	Serine (S)	**3c**	81	>99 : 1	17
4	Leucine (L)	**3d**	93	>99 : 1	6
5	Asparagine (N)	**3e**	82	>99 : 1	17
6	Aspartic acid (D)	**3f**	89	>99 : 1	10
7	Arginine (R)	**3g**	83	>99 : 1	15
8	Lysine (K)	**3h**	94	>99 : 1	4
9	Glutamine (Q)	**3i**	95	>99 : 1	4
10	Histidine (H)	**3j**	85	>99 : 1	12
11	Isoleucine (I)	**3k**	86	>99 : 1	12
12	Methionine (M)	**3l**	93	>99 : 1	5
13	Phenylalanine (F)	**3m**	85	>99 : 1	13
14	Threonine (T)	**3n**	85	>99 : 1	12
15	Proline (P)	**3o**	83	>99 : 1	13
16^*d*^	Cysteine (C)	**3p**	42	>99 : 1	1
17	Tyrosine (Y)	**3q**	93	>99 : 1	5
18	Valine (V)	**3r**	85	>99 : 1	10
19	Tryptophan (W)	**3s**	80	>99 : 1	13
20	Glutamic acid (E)	**3t**	92	>99 : 1	6

^*a*^The reaction was performed with 2.7 μmol peptide, 5.4 μmol benzaldehyde (0.5 M in DMSO), and 13.5 μmol NaBH_3_CN in 300 μL citric acid buffer (pH 6.1) at room temperature for 4–6 h.

^*b*^Determined by the area under 280 nm detection of reaction system.

^*c*^Determined by MS/MS analysis of the products.

^*d*^There was 44% conversion to thiazolidine side product.

**Scheme 2:**
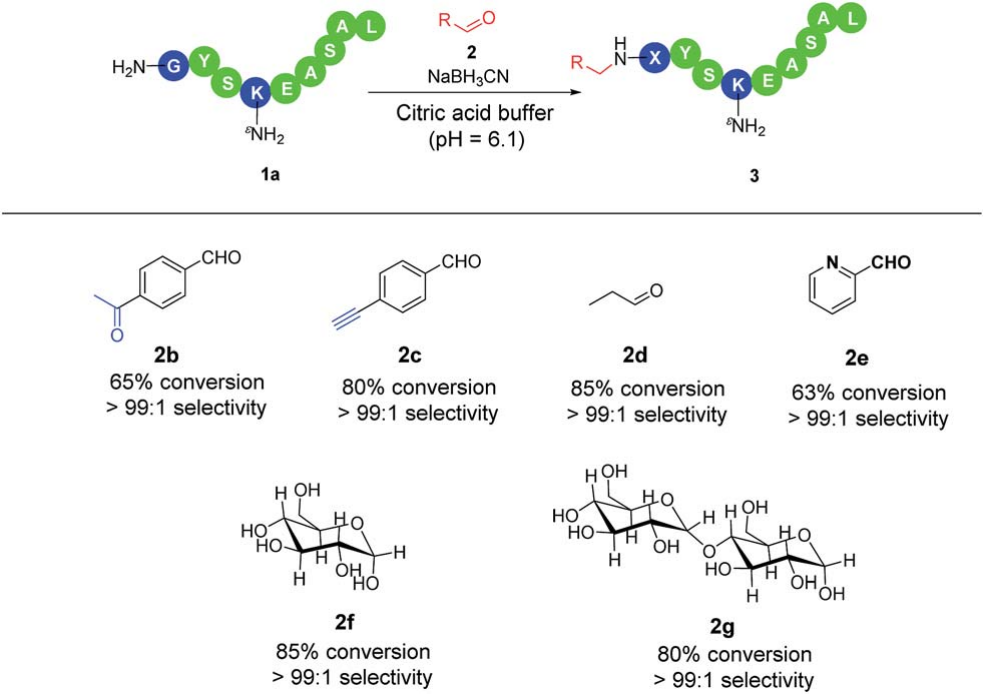
Scope of reaction in various aldehydes. ^a^ The reaction was performed with 2.7 mmol peptide GYSKEASAL, 5.4 mmol aldehyde **2b–e** (0.5 mol L^–1^ in DMSO), and 13.5 mmol NaBH_3_CN in 300 μL citric acid buffer (pH 6.1) at room temperature unless noted. ^b^ Conversion was determined by the area under 280 nm detection. ^c^ Selectivity was determined by MS/MS analysis of products. ^d^ 1.5 equivalents of **2b**, 10 equivalents of **2f**, 10 equivalents of **2g** were used. **2f** or **2g** were reacted in acetic acid buffer (pH 6.0) at 37 °C incubation for 68 h.

Next, we investigated the scope of the reaction towards aldehydes with different functionalities (**2b** to **2g**, Scheme 2). The alkynyl, keto carbonyl, and alkyl group were all well tolerated in the transformation and the corresponding N-terminally modified peptides were achieved in high conversion and >99 : 1 selectivity. Bioorthogonal functional groups (**2b**, **2c**) could be further modified with other molecules. In the case of **2b**, although both aldehyde and ketone are substrates for reductive alkylation, the more electrophilic aldehyde was more reactive and would predominantly react with the N-terminal amine. In the case of 2-PCA (**2e**), high conversion and excellent selectivity was observed. We did not observe the formation of imidazolidinone as described by Francis *et al.* in the absence of hydrides.^9*d*^ This may be due to the lower pH of our condition (pH 6.1 *vs.* 7.5) and the presence of hydride to compete for the nucleophilic addition. Furthermore, the reaction of glucose or maltose (**2f**, **2g**) with **1a** under a slightly modified condition also led to the glycosylated peptides in high conversion and high regioselective manner. In this case, a longer reaction time (2 days) was required most likely due to the equilibrium between keto and enol forms.

**Scheme 3:**
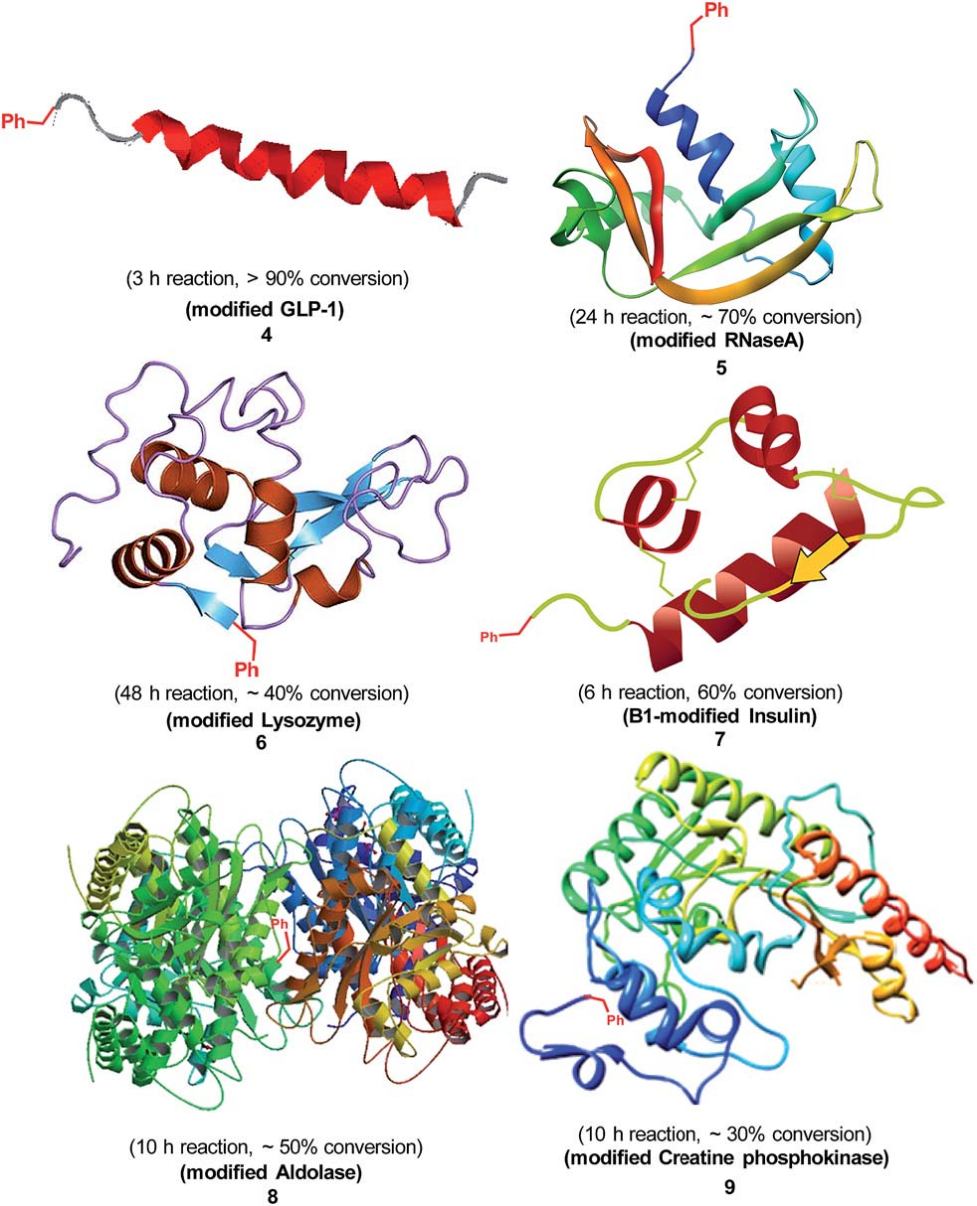
Scope of reaction in native peptides and proteins. The reaction was performed with 3.0 mg protein, 2 equivalents of benzaldehyde (0.5 mol L^–1^ in DMSO) and 5 equivalents of NaBH_3_CN in 300 μL citric acid buffer (pH 6.1) at room temperature. The conversion was determined by LC-MS analysis under 280 nm detection of the reaction system. The N-terminal selectivity was determined by a trypsin digestion method. **7** was treated with dithiothreitol to reduce the internal disulfide bonds to confirm the B chain modification. For modified protein **4**, **7**, the MS/MS analysis of trypsin or dtt digested segments confirmed the N-terminal modification. Single modification was seen for protein **5–9**. See the ESI† for details.

**Scheme 4:**
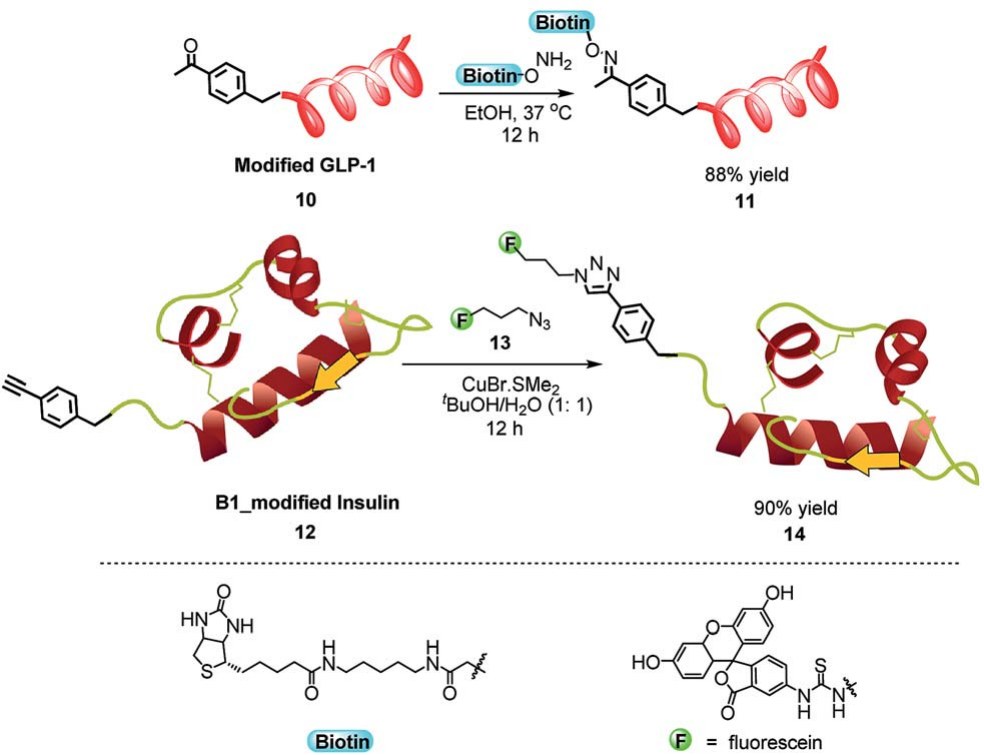
Transformations of modified proteins.

To demonstrate the ability of our method in modifying native peptides and proteins, we investigated the reaction of benzaldehyde and the following proteins: glucagon-like peptide 1 (GLP-1) (with N-terminal histidine and 2 internal lysines), RNase A (with N-terminal lysine and nine internal lysines), lysozyme (with N-terminal lysine and five internal lysines), insulin (with N-terminal glycine on A-chain, N-terminal phenylalanine on B-chain and one internal lysine), aldolase (with N-terminal methionine and 24 internal lysines), and creatine phosphokinase (with N-terminal proline and 36 internal lysines). All these peptides and proteins were selectively modified by benzaldehyde on their N-termini (Scheme 3) with reasonable reaction conversions. In the case of insulin, the modification completely occurred on the N-terminal phenylalanine of the B-chain, which is consistent with previous literature on insulin modification using other methods.^9*b*^ To further demonstrate the utility of this methodology, we directly inserted bioorthogonal groups onto peptides and proteins. First, GLP-1 was reacted with **2b** to yield *para*-acetyl benzylated GLP-1 **10**, which was treated with aminooxy-biotin in ethanol leading to the biotinated product **11** in 88% yield as a stable oxime adduct (Scheme 4). As another example, insulin was reacted with **2c** to form the alkyne-labeled insulin. A 1,3-dipolar cycloaddition between the *para*-ethynyl benzylated insulin **12** and the azide-containing fluorescein **13** gave the fluorescein-labeled insulin **14** with 90% yield. Our method enabled a single-step introduction of bioorthogonal groups with high specificity and efficiency in native peptides and proteins. This is especially useful for some proteins where genetic incorporation of unnatural amino acids with bioorthogonal side chains is difficult.

**Scheme 5:**
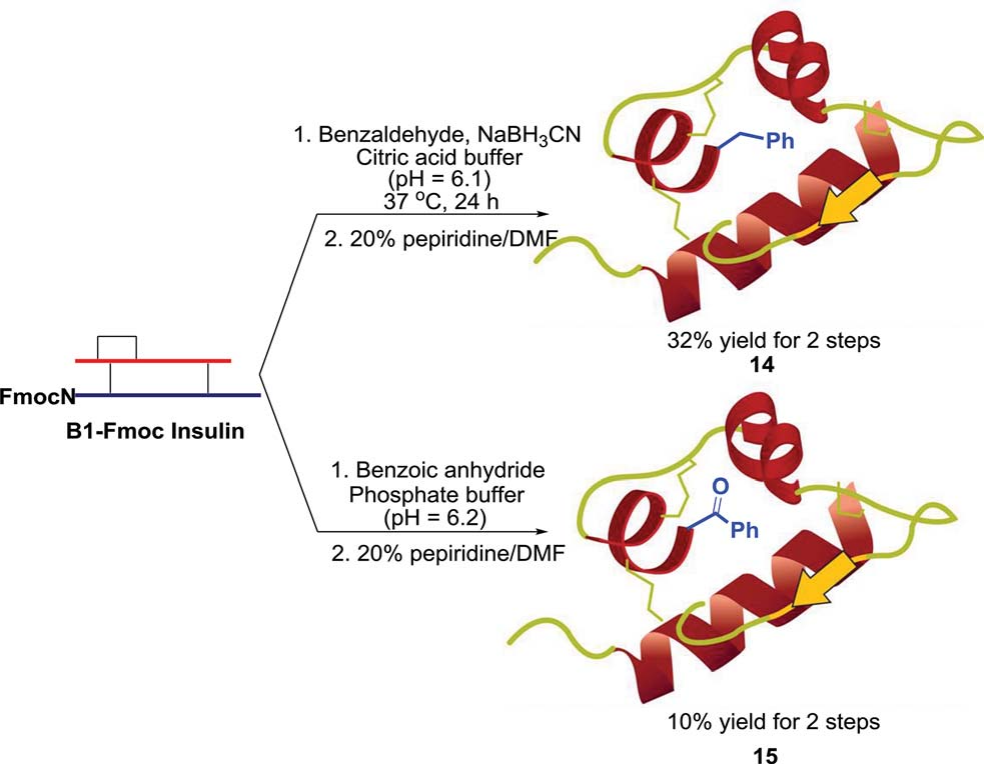
Insulin A chain modification.

**Fig. 2:**
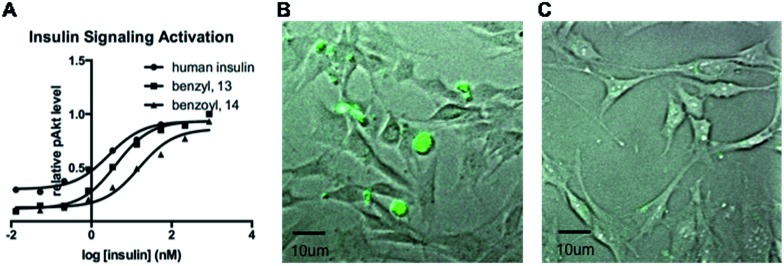
Biological studies of the modified human insulin analogues. (A) Bioactivity of human insulin and modified analogues. (B and C) Insulin uptake study. HEK293 cells were treated with fluorescein-modified insulin 12 (1 μg mL^–1^) for 30 minutes in the absence (B) or presence (C) of human insulin (10 μg mL^–1^). The cells were then washed three times using PBS to remove fluorescein-labeled insulin due to non-specific interactions.

Finally, we investigated the influence of our modification method on protein bioactivity. In this study, we selectively synthesized the modified human insulin A-chain N-terminal amine by either reductive alkylation or acylation (Scheme 5). The resulting A1-benzyl and A1-benzoyl analogues, **13** and **14**, have a similar structure with a difference in the positive charges on the N-terminal amine. Using insulin receptor-overexpressed HEK293 cells to evaluate insulin signaling by measuring the levels of p-Akt at Ser 473, we observed that analogue **16** demonstrated 5-fold lower activity compared to analogue **15**, which maintained a similar bioactivity (1.4-fold reduction) as that of the native insulin (Fig. 2A). This observation is consistent with the previous literature finding that the A1 N-terminal charge is important for insulin bioactivity.^13^ This result further demonstrates the value of the reported method for proteins where the existence of the N-terminal amine is essential for their bioactivity. Furthermore, fluorescein-labeled insulin **14** was incubated in the culture of HEK293 cells for 30 minutes in the absence or presence of human insulin. We observed that **14** was able to bind to insulin receptors and get internalized into the cells, which suggests that the modification does not affect the receptor binding property (Fig. 2B). On the other hand, the presence of human insulin was able to reduce the amount of insulin uptake, which indicates that this binding is specific to insulin receptor (Fig. 2C). Overall, insulin modification through reductive amination yields bioactive analogues that can be further used for biological studies.

## Conclusions

In summary, we have reported an efficient method for utilizing reductive alkylation to selectively introduce N-terminal functionalities on peptides and proteins. Nearly, all N-terminal residues led to high reaction conversion with an exception of N-terminal cysteines, which resulted in thiazolidine derivatives. We demonstrated that the resulting peptide and protein derivatives with bioorthogonal groups can be further modified with molecules, such as fluorophores or biotins, for biological studies. More importantly, in the case of N-terminal modifications, we observed that secondary amines generated from the reductive amination may lead to higher protein bioactivities than amide generated from acylation, as demonstrated in the case of insulin modification. This can be applied to proteins where the N-terminal charge is crucial for protein bioactivity. Our results demonstrated that the use of benzaldehyde derivatives is a facile method for the selective N-terminal functionalization. It is also complementary to other existing methods and may provide derivatives with higher bioactivity. Therefore, this method can be used to site-specifically label proteins-of-interest, and efforts in using this method to generate new peptide and protein derivatives are currently underway.
