# Supplementary material for: Selective N-terminal functionalization of native peptides and proteins
Source: Chem Sci. 2017 Jan 9;8(4):2717–22. doi: 10.1039/c6sc04744k (PMC5426342; doi:10.1039/c6sc04744k)

Electronic Supplementary Material (ESI) for Chemical Science.
This journal is © The Royal Society of Chemistry 2017

Supplementary Information for:

Selective N-terminal Functionalization of Native Peptides and Proteins

Diao Chen, Maria M. Disotuar, Xiaochun Xiong, Yuanxiang Wang, and Danny Hung-Chieh Chou*

E-mail: dchou@biochem.utah.edu

Contents

1. **General Information: S2**
2. **Peptide Synthesis S2**
3. **General procedure for the reductive amination between peptide/protein and aldehyde/carbohydrate........S3**
4. **For synthesis of derivatives 11, 14, 15 and 16: S3**
5. **Biological assays........S7**
6. **Copy of LC chromatogram, MS spectrum, MS/MS spectrum, and trypsin digested segments MS spectrum. S7**

1. General Information:

Chemical and Protein Materials.

Benzaldhyde, n-propionaldhyde, p-acetylbenzaldehyde, p-ethynylbenzaldehyde, 2-pyridinecarboxyaldehyde, glucose, Maltose, sodium cyanoborohydride, Fmoc *N*-hydroxy-succinimide ester, RNase A, Lysozyme were purchase from Sigma-Aldrich and used without purification. Insulin was purchased from Life Technologies Co. Model peptides and GLP-1 were synthesized via Fmoc solid phase peptide synthesis. Dithiothreitol (DTT) was purchased from Gold Bio Technology. Fmoc-protected amino acids were purchased from Protein Technologies Inc. O-(Benzotriazol-1-yl)-N,N,N',N'-tetramethyluronium (HBTU) and 1-[Bis(dimethylamino)methylene]-1H-1,2,3-triazolo[4,5-b]pyridinium 3-oxid hexafluorophosphate (HATU) were purchased from ChemPep. Rink Amide MBHA resin HL was obtained from Novabiochem and Rink amide ChemMatrix was purchased from Biotage. Dimethylformamide (DMF), trifluoroacetic acid (TFA), acetonitrile and ethyl ether were purchased from Fisher Scientific and used as supplied.

HPLC, LC/MS and Mass Spectrometry

Preparative reverse-phase HPLC of crude peptides was performed on Luna 5u C8 100 Å (250 ×10 mm) at 3 mL/min with a water/acetonitrile gradient in 0.1% TFA on an Agilent 1260 HPLC system. Fractions collected from preparative were analyzed by LC/MS on a XBridge C18 5-μm (50 × 2.1 mm) column at 0.4 mL/min with a water/acetonitrile gradient in 0.1% formic acid on an Agilent 6120 Quadrupole LC/MS system. Fractions containing targeted product (based on LC/MS) were collected and lyophilized.

Protein analyses and peptides LC-MS/MS analyses were performed using a hybrid Q-TOF mass spectrometer (Bruker, Maxis ETD-II) equipped with Eksigent Nano-LC 400 system (Captive Spray source for peptides; Microspray source for big protein). The trypsin digested peptide fragments chromatography was performed using Waters Atlantis c18 nano column (0.1 mm × 100 mm size for peptides; 0.3 mm × 100 mm size for proteins) with a $CH_3CN/^{dd}H_2O$ gradient mobile phase containing 0.1% formic acid (flow rate: 0.4 μL/min for peptides; 5 μL/min for big protein).

2. Peptide Synthesis

Peptides XYSKEASAL (X = 20 natural amino acids) and GLP-1 were synthesized via Fmoc solid phase peptide synthesis on a commercial peptide synthesizer (Alstra; Biotage, Inc). Automated peptide synthesis was carried out in a 10 mL reactor vial with the following protocols (for 0.1 mmol scale). For Fmoc deprotection: (i) 4.5 mL of 20% piperidine in DMF; (ii) mix 2 × 3 min (new solvent delivered for each mixing cycle). For amino acid coupling: (i) 1.25 mL of 0.4 M Fmoc-protected amino acid in DMF; (ii) 1.225 mL of 0.4 M HBTU or HATU (HBTU and Rink Amide MBHA resin HL was used for peptides XYSKEASAL [X = 20 natural amino acids]; HATU and H-Rink Amide ChemMatrix for GLP-1) in DMF; (iii) 1.0 mL of 1.0 M DIPEA in DMF; and (iv) mix for 5 min at 75 ^oC (for cysteine and histidine coupling: mix for 10 min at 50 ^oC; for Arginine coupling: mix for 15 min at 50 ^oC and coupling twice). For DMF washing (performed between deprotection and coupling steps): (i) 4.5 mL of DMF; (ii) mix 45 s. Upon completion of the peptide chain, resins were washed

with DCM and dried (using vacuum) for 20 min. Then peptide was cleaved from the resin by exposure to cleavage cocktail for 2.5 h, which were prepared with 12.5 mL TFA, 330 μL water, 330 μL TIS. The peptide was precipitated with ethyl ether at 4 °C, followed by HPLC purification and lyophilization.

3. General procedure for the reductive amination between peptide/protein and aldehyde/carbohydrate

For model peptide reacted with aldehyde:

The mixture of **1** (2.7 μmol) and $NaBH_3CN$ (5 equiv, 13.5 μmol, 1.0 mg) were dissolved in 300 μL citric acid buffer (pH = 6.1). 0.5 M aldehyde/DMSO solution (2 equiv, 5.4 μmol, 13 μL) was added into the system and kept stirring for 4-6 hours at room temperature. The reaction solution was diluted with distilled deionized water (2.0 mL) and subjected to preparative reverse-phase HPLC (Agilent 1260 HPLC system). The corresponding liquid fractions were collected and lyophilized.

For model peptide reacted with carbohydrate:

The mixture of **1a** (2.7 μmol), carbohydrate (10 equiv, 27 μmol) and $NaBH_3CN$ (5 equiv, 13.5 μmol, 1.0 mg) were dissolved in 300 μL acetic acid buffer (pH = 6.0) and incubated at 37 °C (Incubate 20 hours for the reaction with maltose; 2 days for the reaction with glucose). The reaction solution was diluted with distilled deionized water (2.0 mL) and subjected to preparative reverse-phase HPLC (Agilent 1260 HPLC system). The corresponding liquid fractions were collected and lyophilized.

For proteins reacted with benzaldehyde:

The mixture of **protein** (3 mg), $NaBH_3CN$ (5 equiv) was dissolved in 300 μL citric acid buffer (pH = 6.1). 0.5 M benzaldehyde/DMSO solution (2 equiv) was added into the system and kept stirring at room temperature. (3 hours for GLP-1; 6 hours for insulin; 24 hours for RNase; 48 hours for lysozyme; 10 hours for aldolase and phosphokinase). The reaction solution was diluted with distilled deionized water (2.0 mL) and subjected to preparative reverse-phase HPLC (Agilent 1260 HPLC system). The corresponding liquid fractions were collected and lyophilized.

4. For synthesis of derivatives 11, 14, 15 and 16:

Procedure for biotin-modified GLP-1 (11):

The mixture of GLP-1 (3 mg), $NaBH_3CN$ (5 equiv) were dissolved in 300 μL citric acid buffer (pH = 6.1). 0.5 M 4-acetylbenzaldehyde/DMSO solution (2 equiv) was added into the system and kept stirring at room temperature for 3 hours. The reaction solution was diluted with distilled deionized water (2.0 mL) and subjected to preparative reverse-phase HPLC. Intermediate **10** was obtained as white powder after lyophilization (2.6 mg, 83% yield).

Intermediate **10** (2 mg, 0.56 μmol), biotin-ONH_2 (2 equiv, 1.12 μmol; 0.1 mmol/L in DMSO) were dissolved in 200 μL ethanol and incubated for 12 hours. The reaction solution was diluted with distilled deionized water (2.0 mL) and subjected to preparative reverse-phase HPLC. **11** was obtained as white powder after lyophilization (1.9 mg, 88% yield).

Procedure for FITC labeled Insulin (14):

The mixture of insulin (6 mg), $NaBH_3CN$ (5 equiv) were dissolved in 300 μL citric acid buffer (pH = 6.1). 0.5 M 4-ethynylbenzaldehyde/DMSO solution (2 equiv) was added into the system and kept stirring at room temperature for 6 hours. The reaction solution was diluted with distilled deionized water (2.0 mL) and subjected to preparative reverse-phase HPLC. Intermediate **12** was obtained as white powder after lyophilization (3 mg, 55% yield).

Intermediate **12** (3 mg, 0.51 μmol), **13** (1.5 equiv, 0.76 μmol; 0.1 mmol/L in DMSO) were dissolved in ^{t}BuOH/H_2O (150/150 μL) ethanol and incubated for 12 hours. The reaction solution was diluted with distilled deionized water (2.0 mL) and subjected to preparative reverse-phase HPLC. **14** was obtained as white powder after lyophilization (2.9 mg, 90% yield).

Procedure for B1_Fmoc_Insulin:

Insulin (100 mg, 19.6 μmol) was dissolved in 5 mL phosphate buffer solution (pH = 6.2), Fmoc *N*-hydroxy-succinimide ester dissolved in dioxane (0.05 M, 1.5 equiv, 294 μL) was added into the insulin solution. The mixture was kept stirring for 30 minutes. The reaction solution was diluted with distilled deionized water (5 mL) and subjected to preparative reverse-phase HPLC, which gave the corresponding B1_Fmoc_insulin (10 mg).

Procedure for A1_benzyl Insulin (15):

B1_Fmoc_Insulin (10 mg, 1.66 μmmol), $NaBH_3CN$ (5 equiv, 8.29 μmmol) was dissolved in 600 μL citric acid buffer (pH = 6.1). 0.5 M benzaldehyde/DMSO solution (2 equiv, 6.62 μL) was added into the system and incubated at 37 ^oC for 24 hours. The reaction solution was diluted with distilled deionized water (3 mL) and subjected to preparative reverse-phase HPLC, which gave the corresponding modified product. Then it was dissolved in 20% piperidine/DMF and stirred for 20 minutes. The reaction solution was diluted with distilled deionized water (3 mL) and subjected to preparative reverse-phase HPLC, which gave the corresponding A1_benzyl insulin **15** (3.1 mg, 32% yield for two steps).

Procedure for A1_benzoyl Insulin (16):

B1_Fmoc_Insulin (10 mg, 1.66 μmmol) was dissolved in 800 μL phosphate buffer solution (pH = 6.2). 0.05 M benzoic anhydride/dioxane solution (2 equiv, 66.4 μL) was added into the system and incubated at 37 ^oC for 24 hours. The reaction solution was diluted with distilled deionized water (3 mL) and subjected to preparative reverse-phase HPLC, which gave the corresponding bezoylated product. Then it was dissolved in 20% piperidine/DMF and stirred for 20 minutes. The reaction solution was diluted with distilled deionized water (3 mL) and subjected to preparative reverse-phase HPLC, which gave the corresponding A1_benzoyl insulin **14** (0.9 mg, 9% yield).

Table S1. Further condition optimization[a,b,f].

Entry	Buffer	pH	(**3a** + **3a**') (%)	N-selectivity[d]	**3a**'' (%)
1	A	2.5	13	99:1	-
2	A	3.2	63	99:1	25

3	A	4.0	58	98:2	30
4	A	5.1	62	98:2	33
5	A	6.1	78	>99:1	18
6	B	3.3	49	>99:1	38
7	B	4.4	60	95:5	21
8	B	5.3	56	95:5	37
9	B	6.0	60	95:5	22

[a] The reaction was performed with 2.7 µmol peptide, 5.4 µmol, benzaldehyde (0.5 M in DMSO) and 13.5 µmol $NaBH_3CN$ in 300 µL aqueous solvent at room temperature for 6 h unless noted. [b] Buffer A is 25 mmol/L citric acid buffer; buffer B is 25 mmol/L acetic acid buffer. [c] The conversion was calculated by area of the corresponding peak under 280 nm UV detection of reaction system. [d] Determined by MS/MS analysis.

Table S2. Mass spectrometry for the compounds described in this manuscript.

Peptide/Protein	Calculated Mass in Da	Found Mass in Da	Method
**3a**	1013.5	1014.5 $[M+H]^+$	ESI
**3b**	1027.5	1028.5 $[M+H]^+$	ESI
**3c**	1043.5	1044.5 $[M+H]^+$	ESI
**3d**	1069.5	1070.5 $[M+H]^+$	ESI
**3e**	1070.5	1071.5 $[M+H]^+$	ESI
**3f**	1071.5	1072.5 $[M+H]^+$	ESI
**3g**	1112.5	1113.5 $[M+H]^+$	ESI
**3h**	1084.5	1085.5 $[M+H]^+$	ESI
**3i**	1084.5	1085.5 $[M+H]^+$	ESI
**3j**	1093.5	1094.5 $[M+H]^+$	ESI
**3k**	1069.5	1070.5 $[M+H]^+$	ESI
**3l**	1087.5	1088.5 $[M+H]^+$	ESI
**3m**	1103.5	1104.5 $[M+H]^+$	ESI
**3n**	1057.5	1058.5 $[M+H]^+$	ESI
**3o**	1053.5	1054.5 $[M+H]^+$	ESI
**3p**	1059.5	1060.5 $[M+H]^+$	ESI
**3q**	1119.5	1120.5 $[M+H]^+$	ESI
**3r**	1055.5	1056.5 $[M+H]^+$	ESI
**3s**	1142.5	1143.5 $[M+H]^+$	ESI
**3t**	1085.5	1086.5 $[M+H]^+$	ESI
**3u**	1055.5	1056.5 $[M+H]^+$	ESI
**3v**	1037.5	1038.5 $[M+H]^+$	ESI
**3w**	965.5	966.5 $[M+H]^+$	ESI
**3x**	1014.5	1015.5 $[M+H]^+$	ESI
**3y**	1087.5	1088.5 $[M+H]^+$	ESI
**3z**	1249.5	1250.5 $[M+H]^+$	ESI

**4**	-	3442.7 $[M+H]^{+}$	ESI
**5**	-	13764.3 $[M+H]^{+}$	ESI
**6**	-	14773.1 $[M+H]^{+}$	ESI
**7**	5898.5	1475.1 $[M+4H]^{4+}$	ESI
**8**	-	39277.2 $[M+H]^{+}$	ESI
**9**	-	43043.8 $[M+H]^{+}$	ESI
**11**	3868.6	968.4 $[M+4H]^{4+}$	ESI
**14**	6411.6	1069.3 $[M+4H]^{4+}$	ESI
**15**	5898.5	1475.1 $[M+4H]^{4+}$	ESI
**16**	5912.5	1487.7 $[M+4H]^{4+}$	ESI

Table S3. Mass spectrometry observed for the trypsin digested segments.

Peptide/Protein	Found segment 1	Found segment 2	Method
**3a**	544.3	511.1	ESI
**3b**	558.3	489.2	ESI
**3c**	574.2	489.3	ESI
**3d**	600.3	489.3	ESI
**3e**	601.3	489.3	ESI
**3f**	602.3	489.3	ESI
**3g**	643.3	489.5	ESI
**3h**	615.3	489.2	ESI
**3i**	615.3	489.2	ESI
**3j**	624.3	511.0	ESI
**3k**	600.3	489.3	ESI
**3l**	618.3	489.2	ESI
**3m**	634.3	489.2	ESI
**3n**	588.3	489.1	ESI
**3o**	584.6	489.5	ESI
**3p**	589.2	489.3	ESI
**3q**	650.3	489.3	ESI
**3r**	586.5	489.3	ESI
**3s**	673.3	489.2	ESI
**3t**	616.3	489.3	ESI
**3u**	586.3	489.2	ESI
**3v**	568.2	489.2	ESI
**3w**	496.3	489.3	ESI
**3x**	545.3	489.3	ESI
**3y**	618.2	489.3	ESI
**3z**	780.3	511.1	ESI
**4**	1095.0	1005.5	ESI

5. Biological assays

Insulin bioactivity assay

To determine the extent of insulin signaling induced by insulin molecules, pAkt Ser473 levels were measured in HEK293 cells, overexpressed with human IR-B. The cell line was cultured in DMEM with 10% FBS, pen/strep and 2 ug/mL puromycin. For the assay, 40,000 cells per well were plated in a 96-well plates with culture media containing 1% FBS. 24 hours later, 50 uL of insulin solution was pipetted into each well after the removal of the original media. After a 30-min treatment, the insulin solution was aspirated and the HTRF pAkt Ser473 kit (Cisbio, Massachusetts, USA) was used to measure the intracellular level of pAkt Ser473. Briefly, the cells were first treated with cell lysis buffer (50 uL per well) for 1 hour under mild shaking. 16uL of cell lysate was then added to 4uL of detecting reagent in a white 384-well plate. After 4-hour incubation, the plate was read in a Synergy Neo plate reader (Biotek, Vermont, USA). The data was processed according to the manufacturer's protocol.

Fluorescein-labeled insulin imaging assay

A competitive binding imaging assay was used to determine the uptake of fluorescein-labeled insulin in the presence and absence of human insulin. First, a mouse fibroblast cell line, NIH 3T3, overexpressed with human IR-B was cultured in DMEM with 10% FBS, pen/strep and 2 ug/mL puromycin. For the assay, 20,000 cells per well were plated in a 96-well plates with culture media containing 1% FBS. 24 hours later, the media was aspirated and 1 µg/mL of fluorescein-labeled insulin was added to 16 wells and 10 µg/mL of human insulin was added to 8 wells containing 1 µg/mL fluorescein-insulin. These insulins were dissolved in an imaging buffer consisting of 140 mM NaCl, 2.5 mM KCl, 1.8 mM $CaCl_2$, 1 mM $MgCl_2$, 20 mM Hepes (mOsm = 300) pH 7.4 (Life Technologies Corporation). Immediately after addition of insulin solution, cells were imaged for a period of 30 minutes using a GFP filter and 10X magnification on an Axio Observer.A1.

6. Copy of LC chromatogram, MS spectrum, MS/MS spectrum, and trypsin digested segments MS spectrum.

TIC for **3a**:

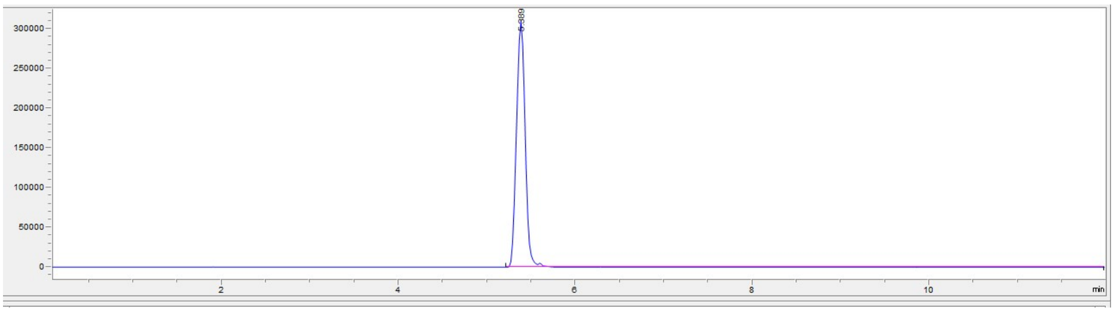

MS Spectrum for **3a**:

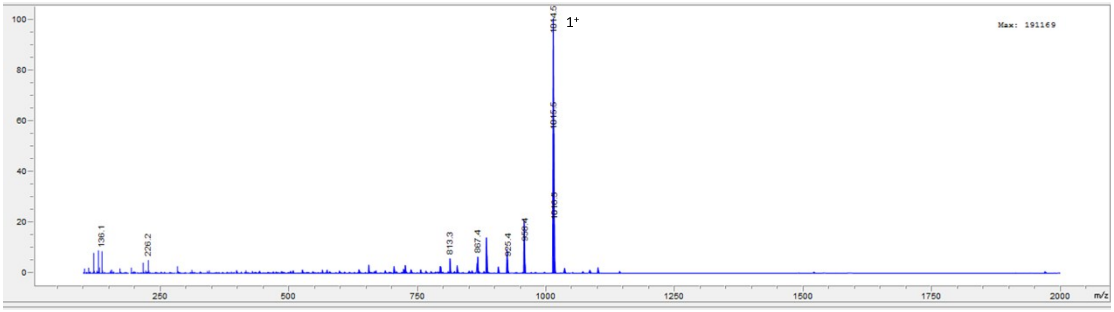

MS MS Spectrum for **3a**:

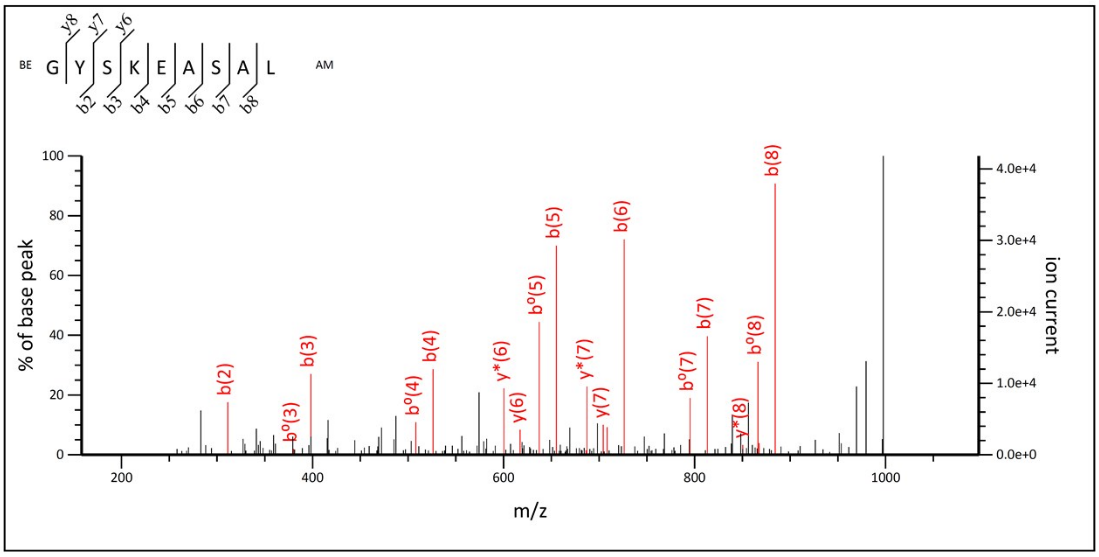


```
Monoisotopic mass of neutral peptide Mr(calc): 1013.5182
Variable modifications:
N-term : Benzyl (N-term)
C-term : Amidated (C-term)
Ions Score: 77   Expect: 1.8e-008    (help)
```

#	b	b*	b^0	Seq.	y	y*	y^0	#
1	148.0757			G				9
2	**311.1390**			Y	**867.4571**	**850.4305**	849.4465	8
3	**398.1710**		**380.1605**	S	**704.3937**	**687.3672**	686.3832	7
4	**526.2660**	509.2395	**508.2554**	K	**617.3617**	**600.3352**	599.3511	6
5	**655.3086**	638.2821	**637.2980**	E	489.2667		471.2562	5
6	**726.3457**	709.3192	**708.3352**	A	360.2241		342.2136	4
7	**813.3777**	796.3512	**795.3672**	S	289.1870		271.1765	3
8	**884.4149**	867.3883	**866.4043**	A	202.1550			2
9				L	131.1179			1

Trypsin digested MS spectrum for **3a**:

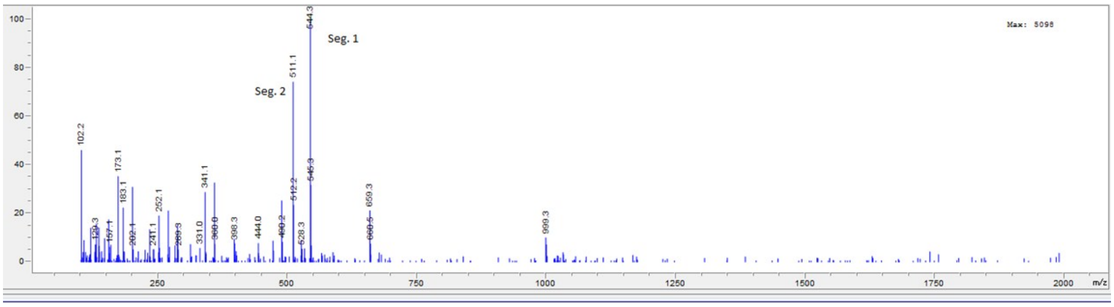

TIC for **3b**:

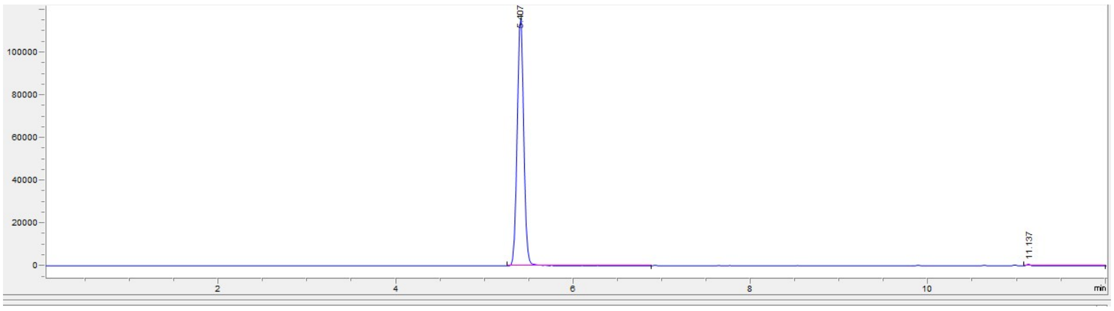

MS Spectrum for **3b**:

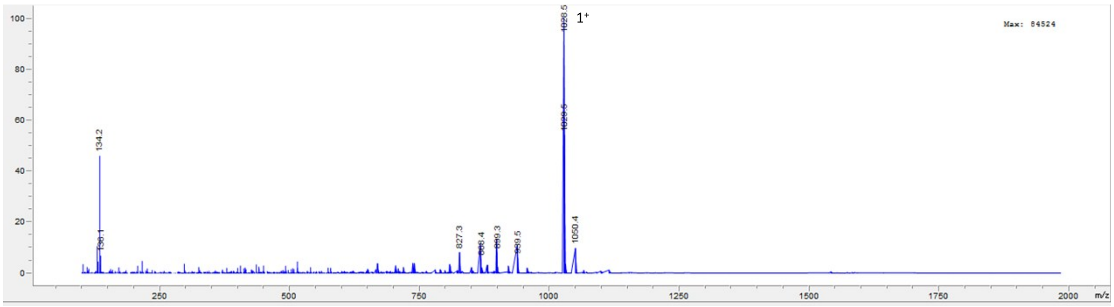

MS MS Spectrum for **3b**:

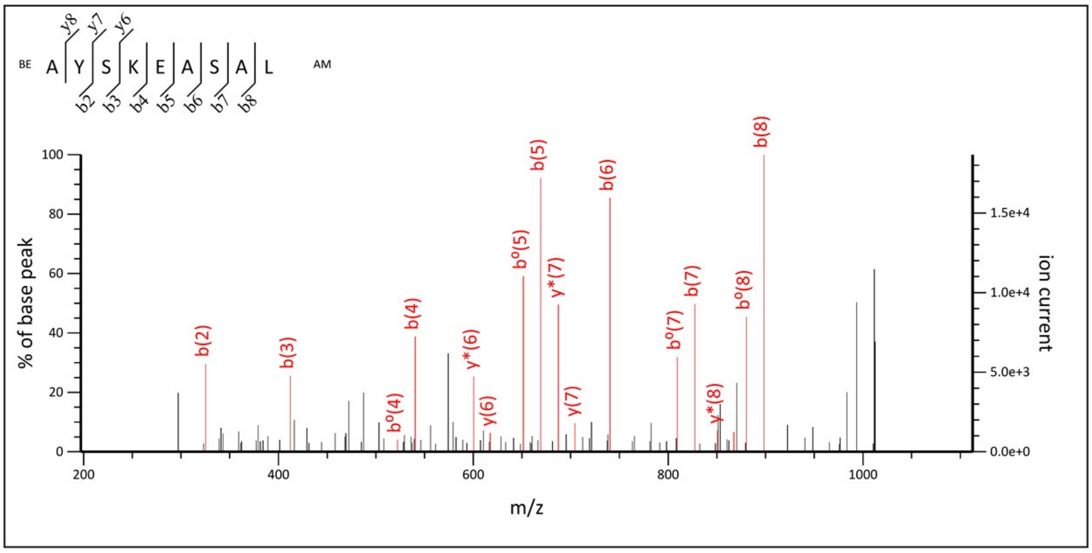

Monoisotopic mass of neutral peptide Mr(calc): 1027.5338
Fixed modifications: Amidated (Protein C-term) (apply to specified residues or termini only)
Variable modifications:
N-term : Benzyl (Protein N-term)
C-term : Amidated (C-term)
Ions Score: 77 **Expect:** 2e-008 (help)

#	b	b*	b⁰	Seq.	y	y*	y⁰	#
1	162.0913			A				9
2	**325.1547**			Y	**867.4571**	**850.4305**	849.4465	8
3	**412.1867**		394.1761	S	**704.3937**	**687.3672**	686.3832	7
4	**540.2817**	523.2551	**522.2711**	K	**617.3617**	**600.3352**	599.3511	6
5	**669.3243**	652.2977	**651.3137**	E	489.2667		471.2562	5
6	**740.3614**	723.3348	722.3508	A	360.2241		342.2136	4
7	**827.3934**	810.3668	**809.3828**	S	289.1870		271.1765	3
8	**898.4305**	881.4040	**880.4199**	A	202.1550			2

Trypsin digested MS spectrum for **3b**:

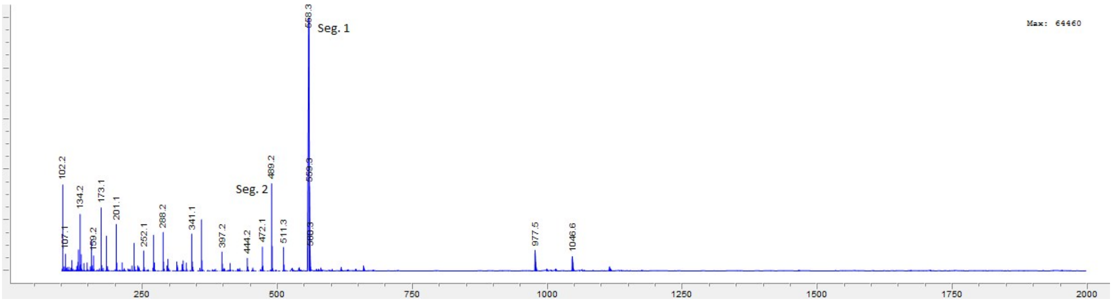

TIC for **3c**:

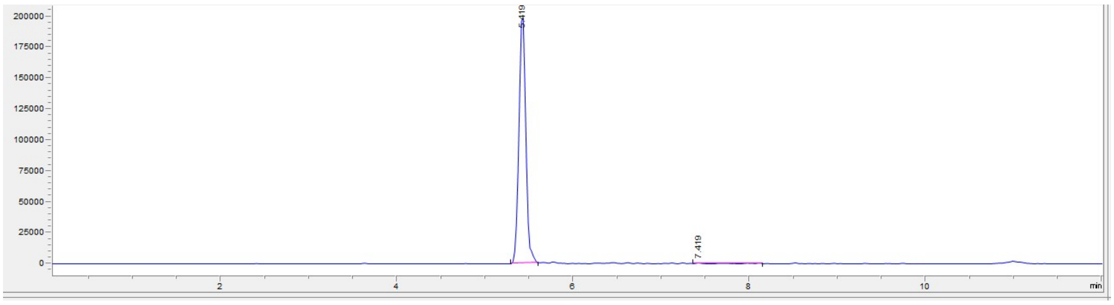

MS Spectrum for **3c**:

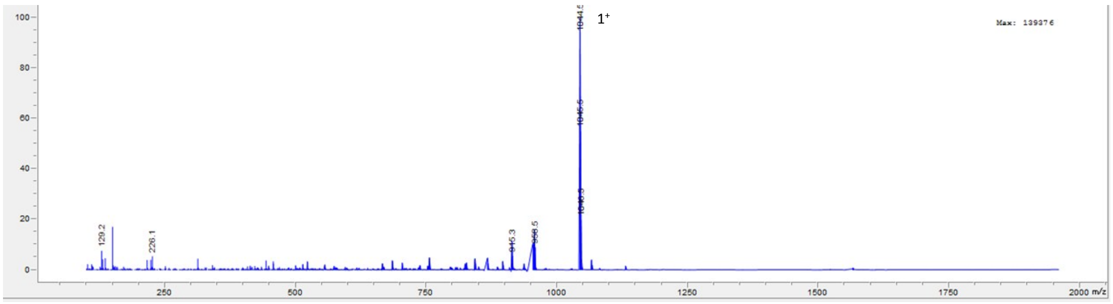

MS-MS Spectrum for **3c**:

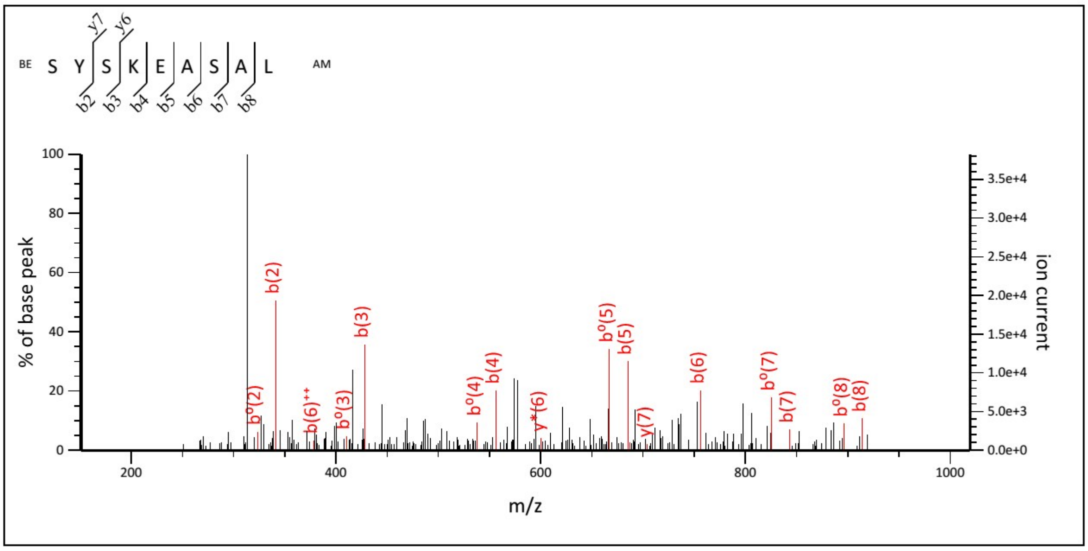

Monoisotopic mass of neutral peptide Mr(calc): 1043.5287
Variable modifications:
N-term : Benzyl (Protein N-term)
C-term : Amidated (C-term)
Ions Score: 31 **Expect:** 0.00086 (help)

#	b	b^{++}	b*	b^{*++}	b^{0}	b^{0++}	Seq.	y	y^{++}	y*	y^{*++}	y^{0}	y^{0++}	#
1	178.0863	89.5468			160.0757	80.5415	S							9
2	**341.1496**	171.0784			**323.1390**	162.0731	Y	867.4571	434.2322	850.4305	425.7189	849.4465	425.2269	8
3	**428.1816**	214.5944			**410.1710**	205.5892	S	**704.3937**	352.7005	**687.3672**	344.1872	686.3832	343.6952	7
4	**556.2766**	278.6419	539.2500	270.1287	**538.2660**	269.6366	K	617.3617	309.1845	**600.3352**	300.6712	599.3511	300.1792	6
5	**685.3192**	343.1632	668.2926	334.6499	**667.3086**	334.1579	E	489.2667	245.1370			471.2562	236.1317	5
6	**756.3563**	**378.6818**	739.3297	370.1685	738.3457	369.6765	A	360.2241	180.6157			342.2136	171.6104	4
7	**843.3883**	422.1978	826.3618	413.6845	**825.3777**	413.1925	S	289.1870	145.0972			271.1765	136.0919	3
8	**914.4254**	457.7163	897.3989	449.2031	**896.4149**	448.7111	A	202.1550	101.5811					2
9							L	131.1179	66.0626					1

Trypsin digested MS spectrum for **3c**:

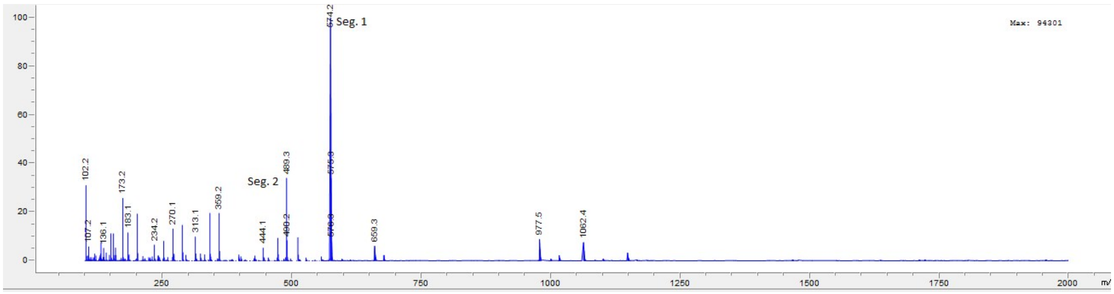

TIC for **3d**:

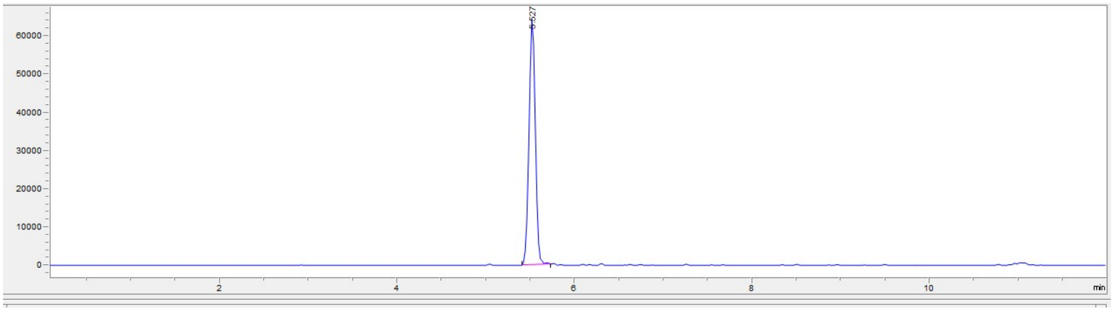

MS Spectrum **3d**:

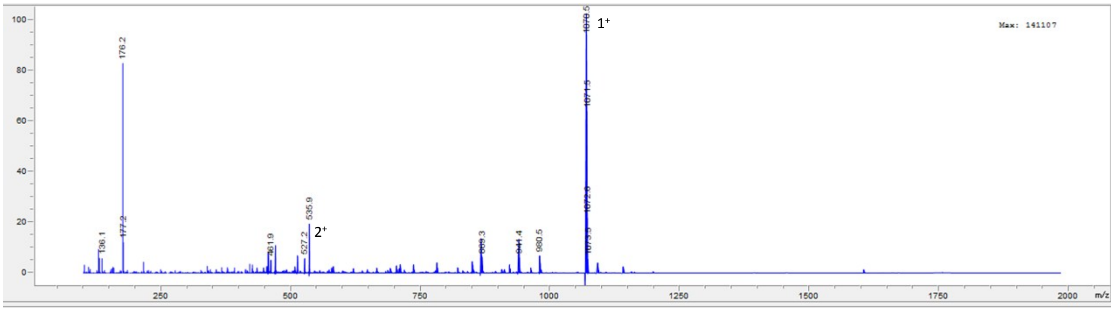

MS MS Spectrum **3d**:

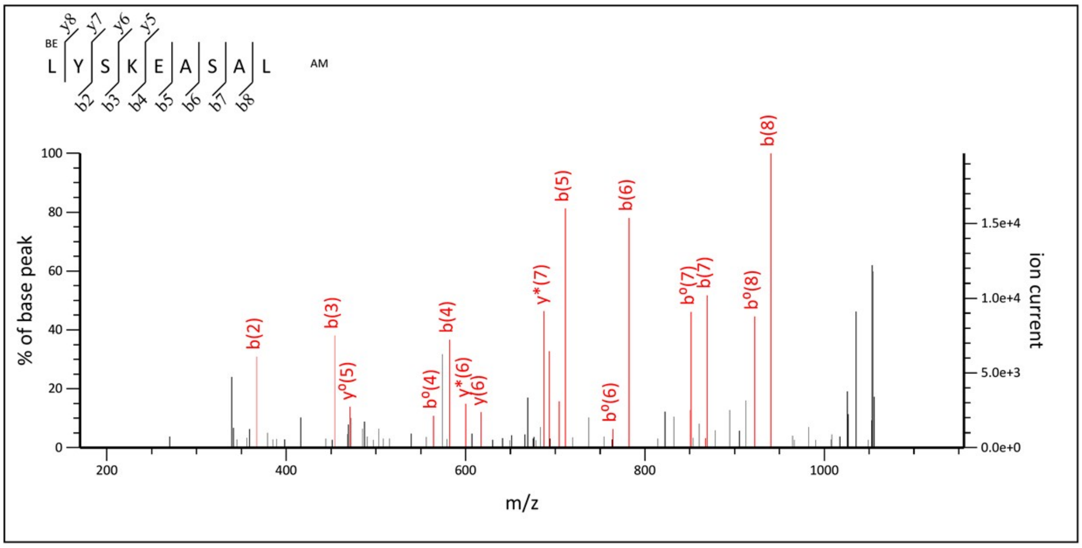

```
Monoisotopic mass of neutral peptide Mr(calc): 1069.5808
Variable modifications:
L1      : Benzyl_L (L)
C-term : Amidated (C-term)
Ions Score: 57  Expect: 2.2e-006   (help)
```

#	b	b*	b^0	Seq.	y	y*	y^0	#
1	204.1383			L				9
2	**367.2016**			Y	**867.4571**	**850.4305**	849.4465	8
3	**454.2336**		436.2231	S	**704.3937**	**687.3672**	686.3832	7
4	**582.3286**	565.3021	**564.3180**	K	**617.3617**	**600.3352**	599.3511	6
5	**711.3712**	694.3447	**693.3606**	E	489.2667		**471.2562**	5
6	**782.4083**	765.3818	**764.3978**	A	360.2241		342.2136	4
7	**869.4403**	852.4138	**851.4298**	S	289.1870		271.1765	3
8	**940.4775**	923.4509	**922.4669**	A	202.1550			2
9				L	131.1179			1

Trypsin digested MS spectrum for **3d** (seg. 1):

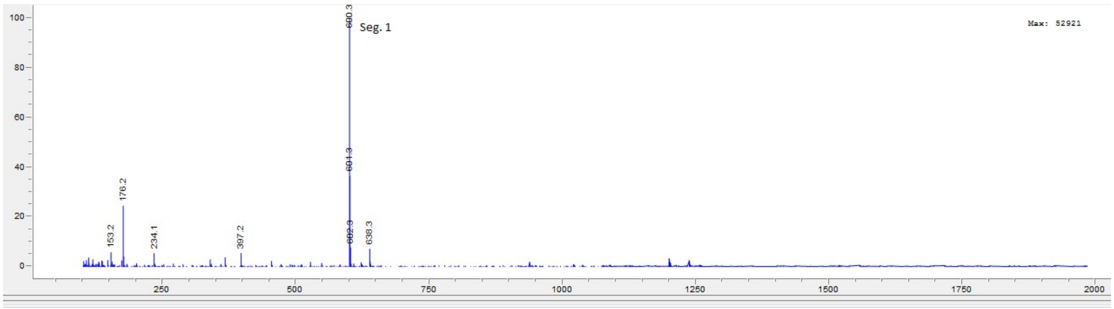

Trypsin digested MS spectrum for **3d** (seg. 2):

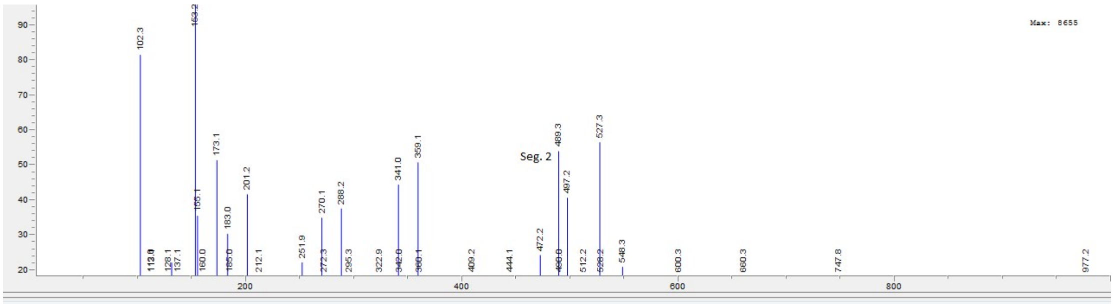

TIC for **3e**:

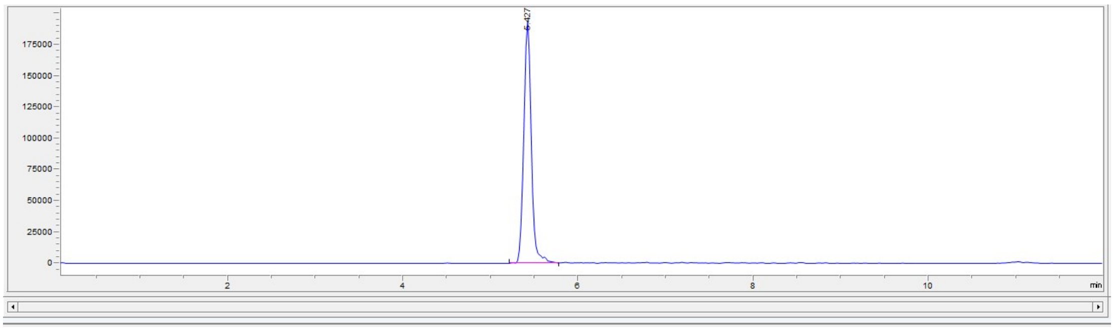

MS Spectrum **3e**:

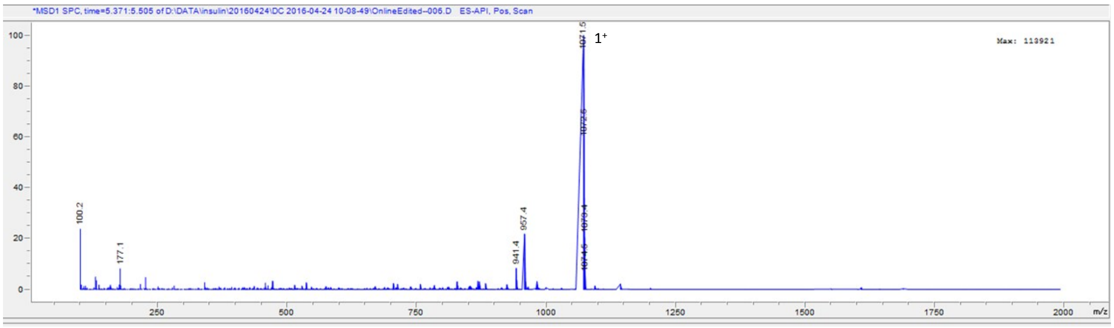

MS MS Spectrum **3e**:

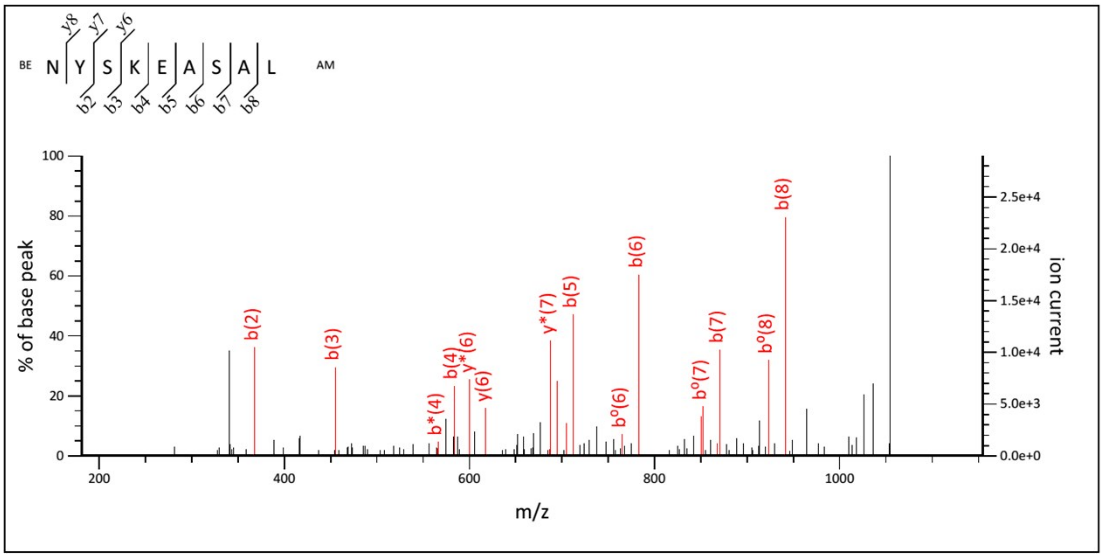

Monoisotopic mass of neutral peptide Mr(calc): 1070.5396
Variable modifications:
N-term : Benzyl (Protein N-term)
C-term : Amidated (Protein C-term)
Ions Score: 59 **Expect:** 1.3e-006 (help)

#	b	b*	b⁰	Seq.	y	y*	y⁰	#
1	205.0972	188.0706		N				9
2	**368.1605**	351.1339		Y	**867.4571**	**850.4305**	849.4465	8
3	**455.1925**	438.1660	437.1819	S	**704.3937**	**687.3672**	**686.3832**	7
4	**583.2875**	**566.2609**	**565.2769**	K	**617.3617**	**600.3352**	599.3511	6
5	**712.3301**	695.3035	**694.3195**	E	489.2667		471.2562	5
6	**783.3672**	766.3406	**765.3566**	A	360.2241		342.2136	4
7	**870.3992**	853.3727	**852.3886**	S	289.1870		271.1765	3
8	**941.4363**	924.4098	**923.4258**	A	202.1550			2
9				L	131.1179			1

Trypsin digested MS spectrum for **3e:**

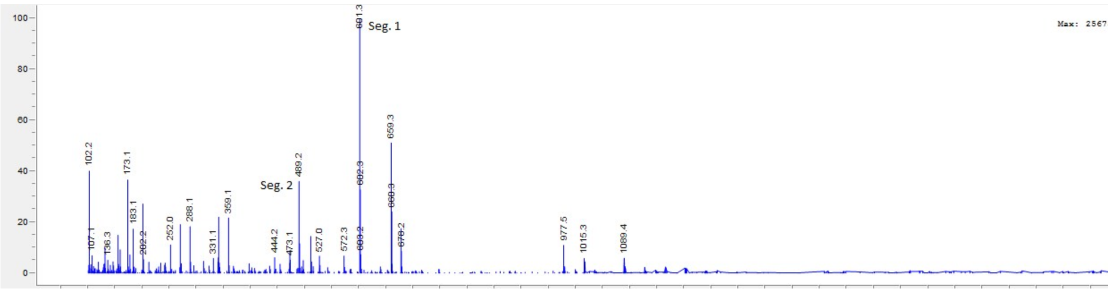

TIC for **3f**:

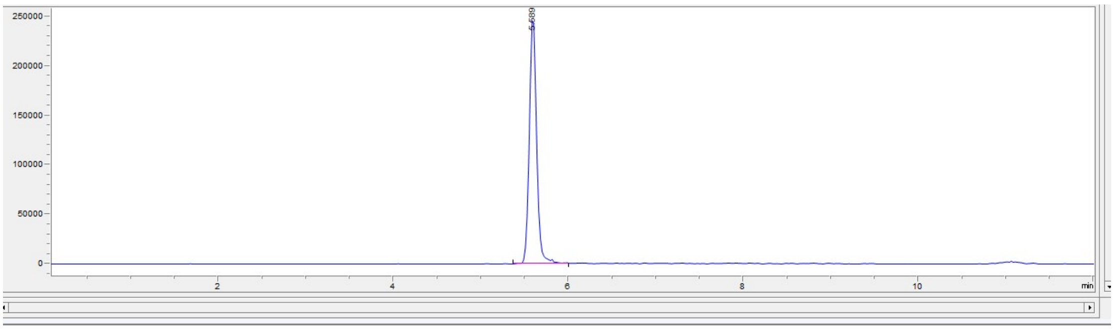

MS Spectrum **3f**:

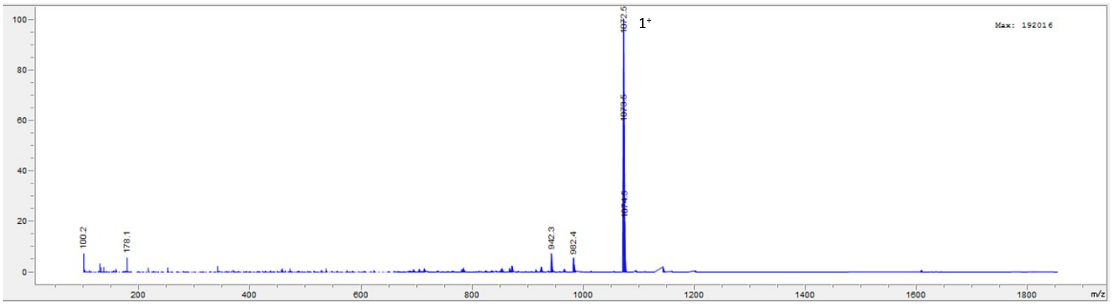

MS MS Spectrum **3f**:

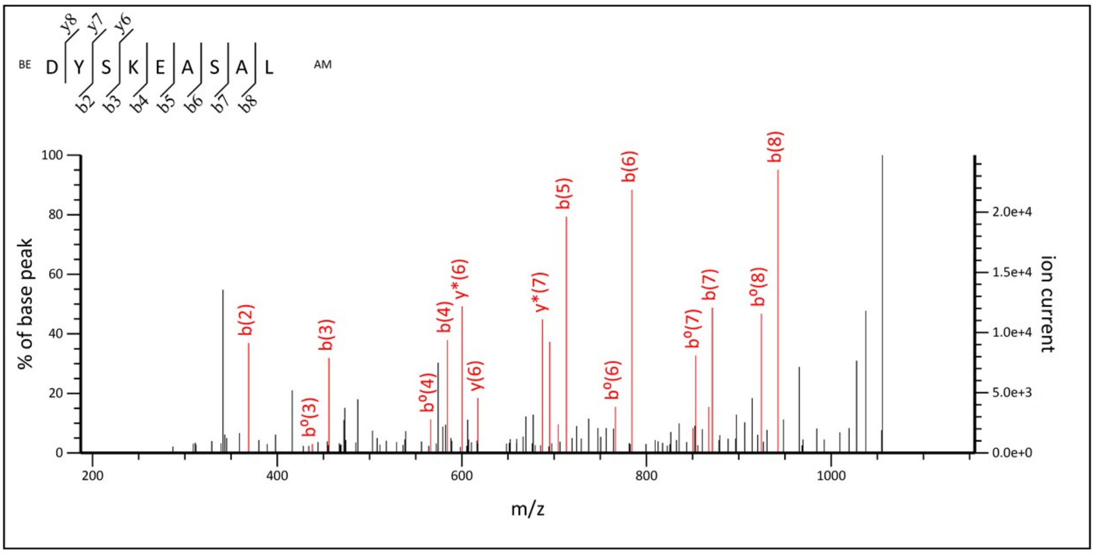

Monoisotopic mass of neutral peptide Mr(calc): 1071.5236
Variable modifications:
N-term : Benzyl (Protein N-term)
C-term : Amidated (Protein C-term)
Ions Score: 65 **Expect:** 3.2e-007 (help)

#	b	b*	b^0	Seq.	y	y*	y^0	#
1	206.0812		188.0706	D				9
2	**369.1445**		351.1339	Y	**867.4571**	**850.4305**	849.4465	8
3	**456.1765**		**438.1660**	S	**704.3937**	**687.3672**	686.3832	7
4	**584.2715**	567.2449	**566.2609**	K	**617.3617**	**600.3352**	599.3511	6
5	**713.3141**	696.2875	**695.3035**	E	489.2667		471.2562	5
6	**784.3512**	767.3246	**766.3406**	A	360.2241		342.2136	4
7	**871.3832**	854.3567	**853.3727**	S	289.1870		271.1765	3
8	**942.4203**	925.3938	**924.4098**	A	202.1550			2
9				L	131.1179			1

Trypsin digested MS spectrum for **3f**:

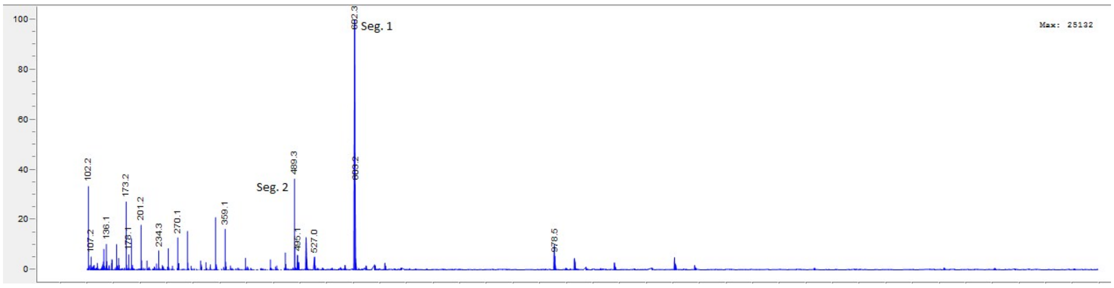

TIC for **3g**:

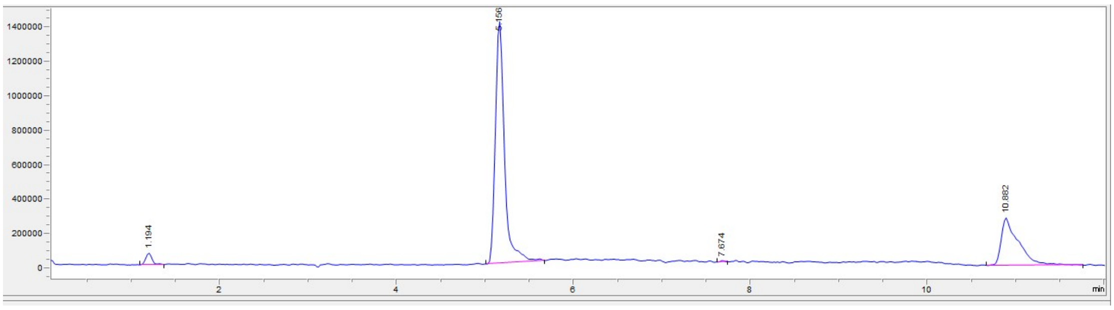

MS Spectrum **3g**:

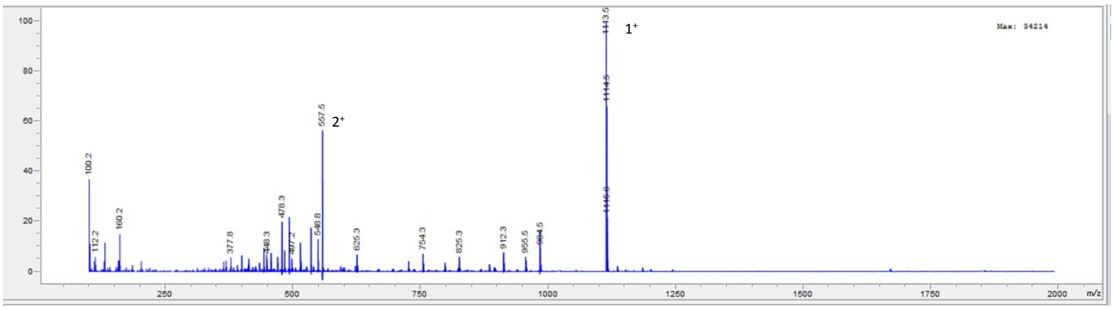

MS MS Spectrum **3g**:

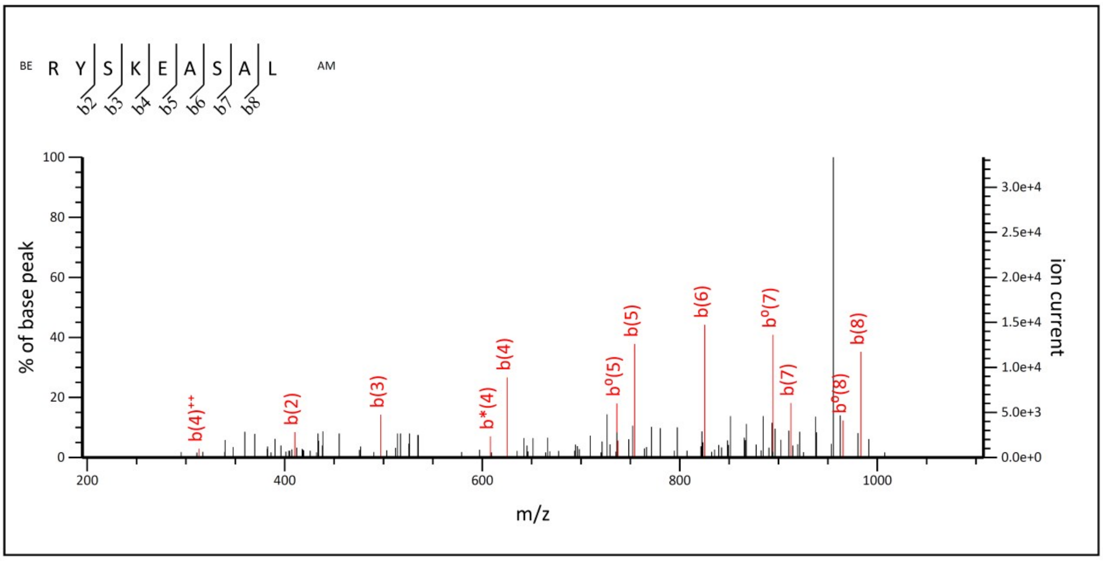

Monoisotopic mass of neutral peptide Mr(calc): 1112.5978
Variable modifications:
N-term : Benzyl (Protein N-term)
C-term : Amidated (Protein C-term)
Ions Score: 31 **Expect:** 0.00074 (help)

#	b	b^{++}	b^{*}	b^{*++}	b^{0}	b^{0++}	Seq.	y	y^{++}	y^{*}	y^{*++}	y^{0}	y^{0++}	#
1	247.1553	124.0813	230.1288	115.5680			R							9
2	**410.2187**	205.6130	393.1921	197.0997			Y	867.4571	434.2322	850.4305	425.7189	849.4465	425.2269	8
3	**497.2507**	249.1290	480.2241	240.6157	479.2401	240.1237	S	704.3937	352.7005	687.3672	344.1872	686.3832	343.6952	7
4	**625.3457**	**313.1765**	**608.3191**	304.6632	607.3351	304.1712	K	617.3617	309.1845	600.3352	300.6712	599.3511	300.1792	6
5	**754.3882**	377.6978	**737.3617**	369.1845	**736.3777**	368.6925	E	489.2667	245.1370			471.2562	236.1317	5
6	**825.4254**	413.2163	808.3988	404.7030	807.4148	404.2110	A	360.2241	180.6157			342.2136	171.6104	4
7	**912.4574**	456.7323	895.4308	448.2191	**894.4468**	447.7271	S	289.1870	145.0972			271.1765	136.0919	3
8	**983.4945**	492.2509	966.4680	483.7376	**965.4839**	483.2456	A	202.1550	101.5811					2
9							L	131.1179	66.0626					1

Trypsin digested MS spectrum for **3g**:

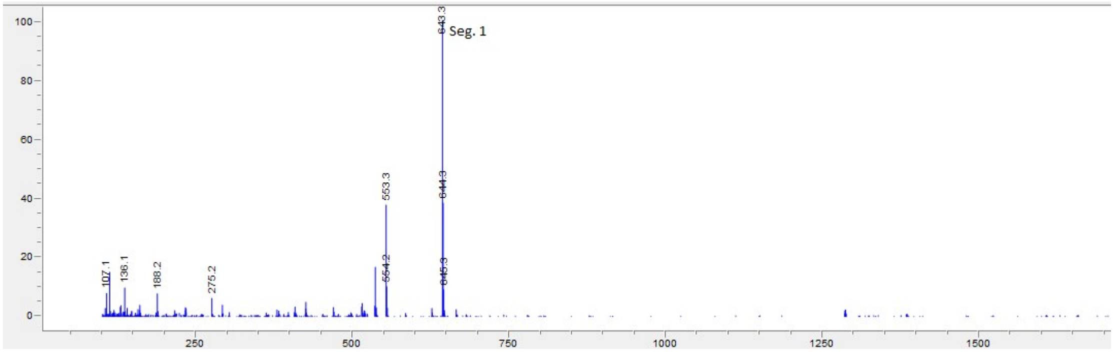

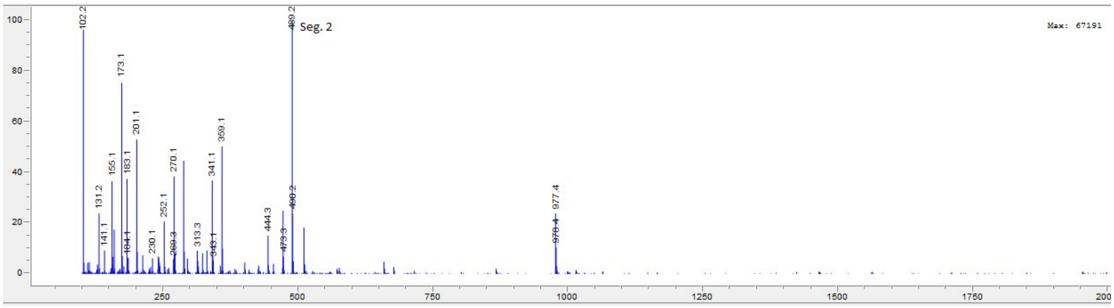

TIC for **3h**:

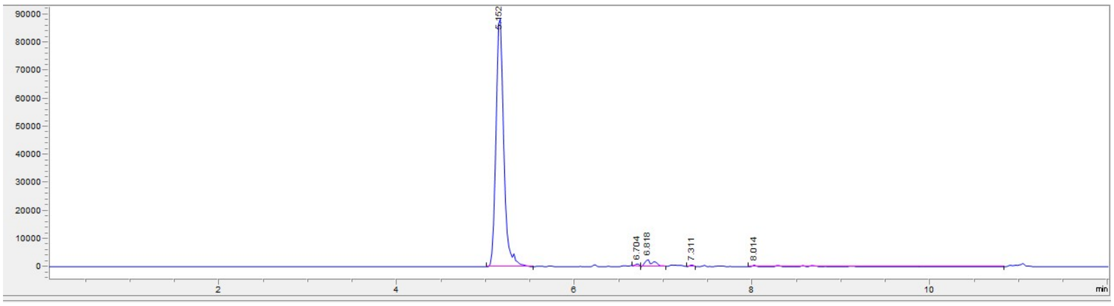

MS Spectrum **3h**:

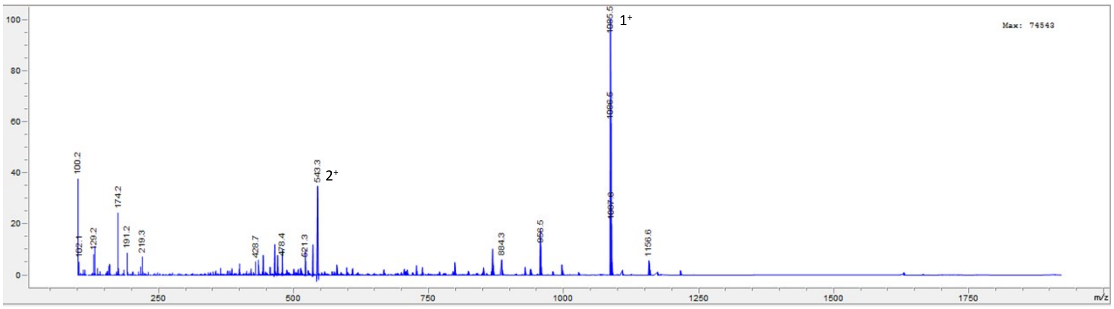

MS MS Spectrum **3h**:

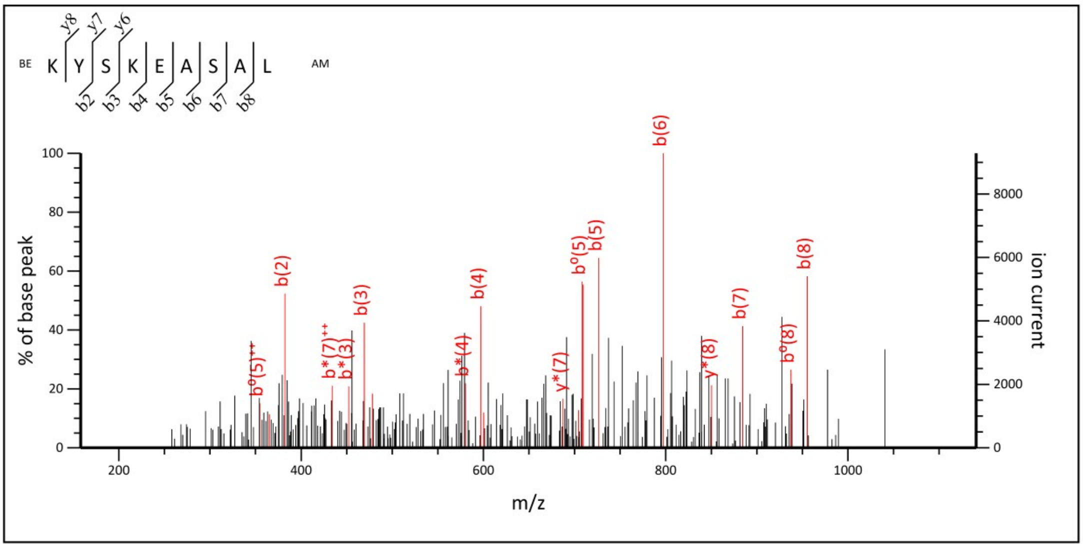

Monoisotopic mass of neutral peptide Mr(calc): 1084.5916
Variable modifications:
N-term : Benzyl (Protein N-term)
C-term : Amidated (Protein C-term)
Ions Score: 23 **Expect:** 0.0053 (help)

#	b	b^{++}	b*	b*$^{++}$	b^{0}	b^{0++}	Seq.	y	y^{++}	y*	y*$^{++}$	y^{0}	y^{0++}	#
1	219.1492	110.0782	202.1226	101.5650			K							9
2	**382.2125**	191.6099	**365.1860**	183.0966			Y	867.4571	434.2322	**850.4305**	425.7189	849.4465	425.2269	8
3	**469.2445**	235.1259	**452.2180**	226.6126	451.2340	226.1206	S	**704.3937**	352.7005	**687.3672**	344.1872	686.3832	343.6952	7
4	**597.3395**	299.1734	**580.3130**	290.6601	579.3289	290.1681	K	617.3617	309.1845	**600.3352**	300.6712	599.3511	300.1792	6
5	**726.3821**	363.6947	**709.3556**	355.1814	**708.3715**	**354.6894**	E	489.2667	245.1370			471.2562	236.1317	5
6	**797.4192**	399.2132	780.3927	390.7000	779.4087	390.2080	A	360.2241	180.6157			342.2136	171.6104	4
7	**884.4512**	442.7293	867.4247	**434.2160**	866.4407	**433.7240**	S	289.1870	145.0972			271.1765	136.0919	3
8	**955.4884**	**478.2478**	938.4618	469.7345	**937.4778**	**469.2425**	A	202.1550	101.5811					2
9							L	131.1179	66.0626					1

Trypsin digested MS spectrum for **3h**:

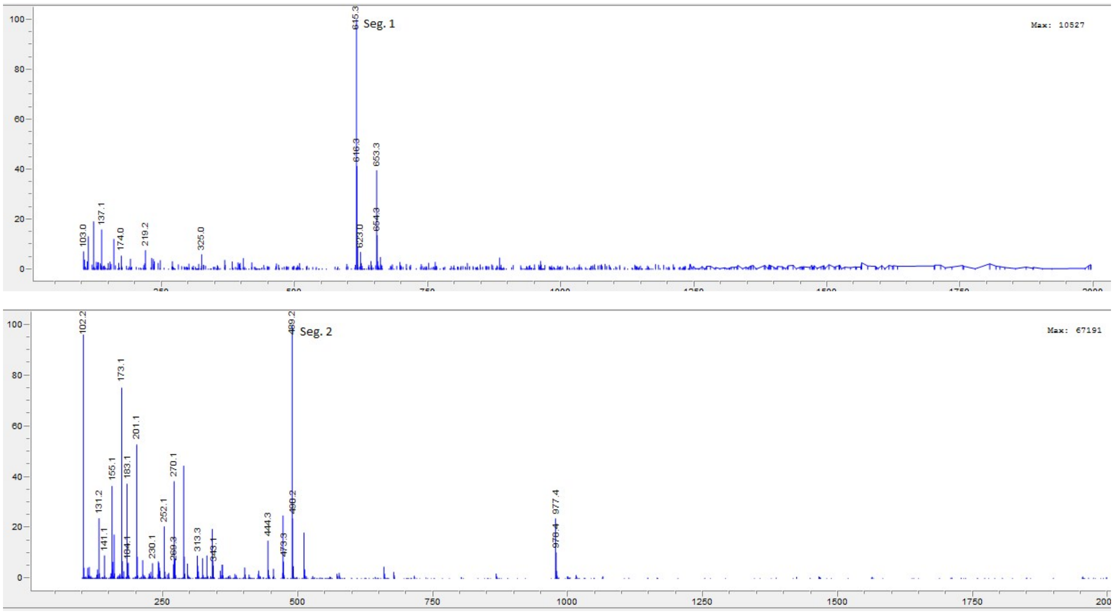

TIC for **3i**:

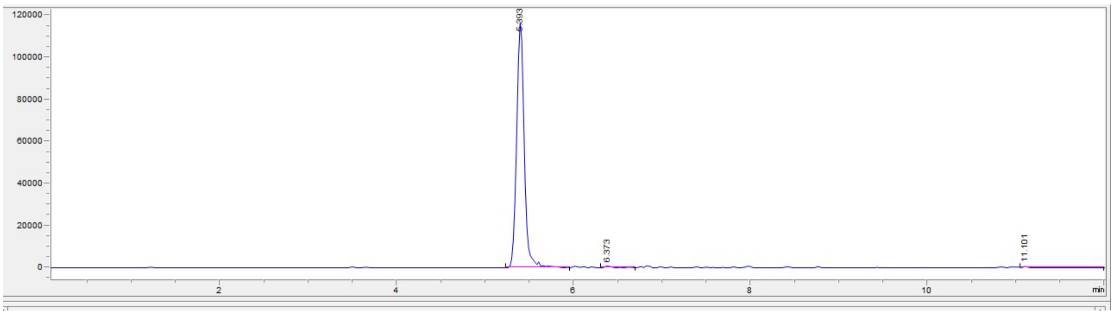

MS Spectrum **3i**:

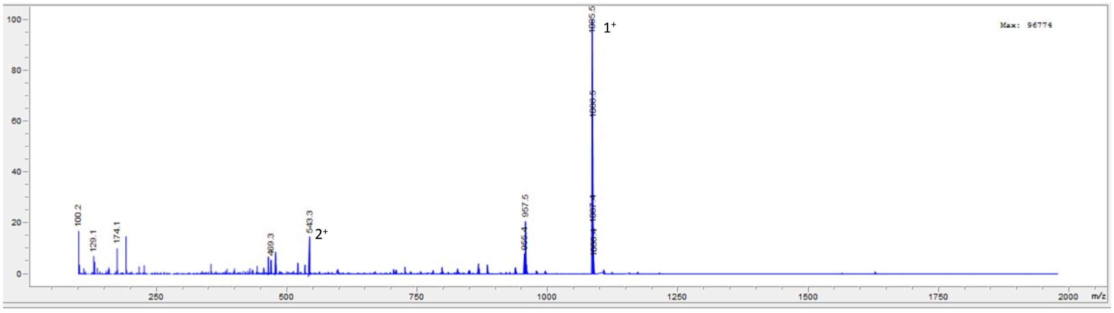

MS MS Spectrum **3i**:

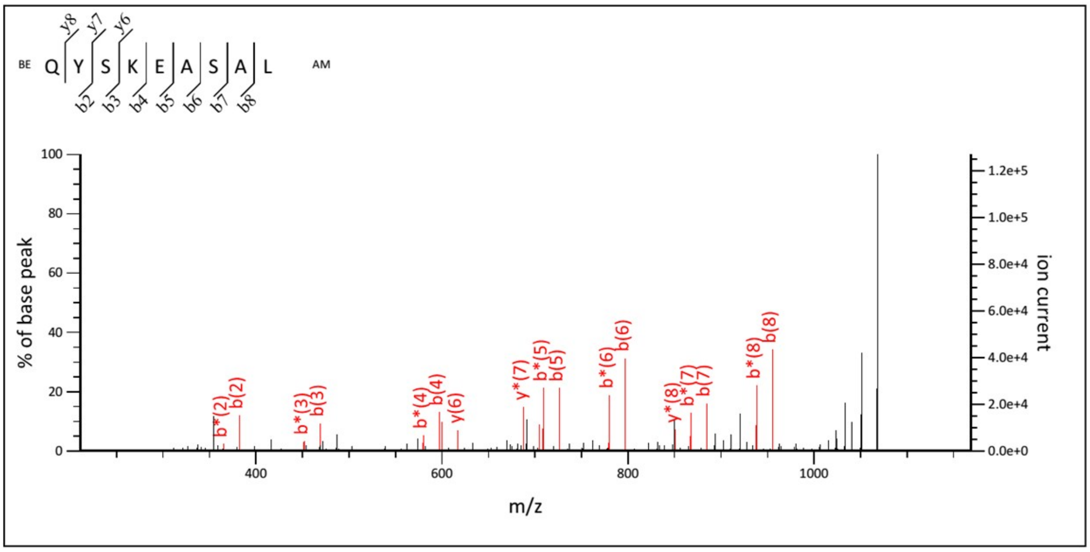

Monoisotopic mass of neutral peptide Mr(calc): 1084.5553
Variable modifications:
N-term : Benzyl (Protein N-term)
C-term : Amidated (Protein C-term)
Ions Score: 62 **Expect:** 5.7e-007 (help)

#	b	b*	b⁰	Seq.	y	y*	y⁰	#
1	219.1128	202.0863		Q				9
2	382.1761	365.1496		Y	867.4571	850.4305	849.4465	8
3	469.2082	452.1816	451.1976	S	704.3937	687.3672	686.3832	7
4	597.3031	580.2766	579.2926	K	617.3617	600.3352	599.3511	6
5	726.3457	709.3192	708.3352	E	489.2667		471.2562	5
6	797.3828	780.3563	779.3723	A	360.2241		342.2136	4
7	884.4149	867.3883	866.4043	S	289.1870		271.1765	3
8	955.4520	938.4254	937.4414	A	202.1550			2
9				L	131.1179			1

Trypsin digested MS spectrum for **3i**:

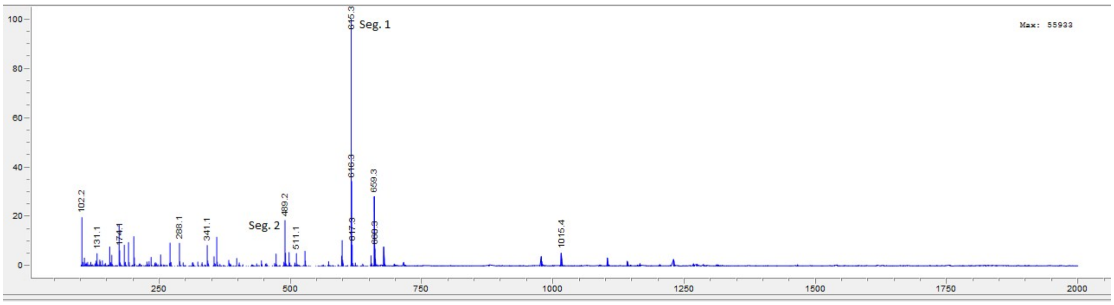

TIC for **3j**:

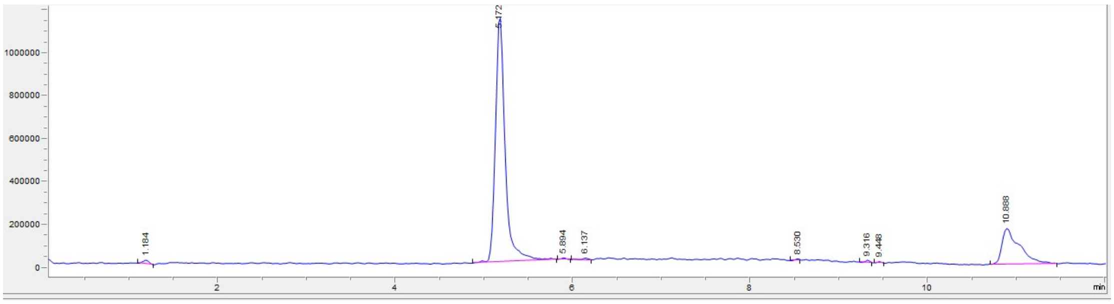

MS Spectrum **3j**:

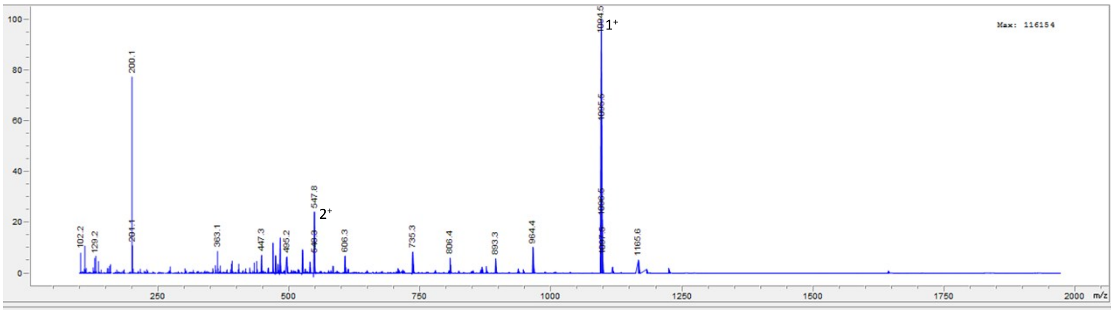

MS MS Spectrum **3j**:

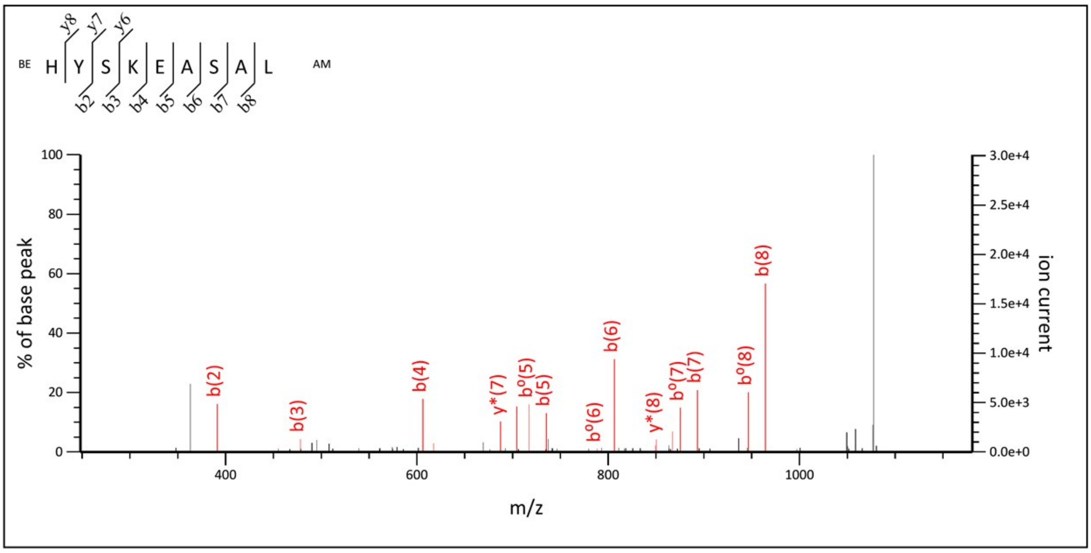

Monoisotopic mass of neutral peptide Mr(calc): 1093.5556
Variable modifications:
N-term : Benzyl (Protein N-term)
C-term : Amidated (Protein C-term)
Ions Score: 49 **Expect:** 1.2e-005 (help)

#	b	b*	b^0	Seq.	y	y*	y^0	#
1	228.1131			H				9
2	**391.1765**			Y	**867.4571**	**850.4305**	**849.4465**	8
3	**478.2085**		460.1979	S	**704.3937**	**687.3672**	686.3832	7
4	**606.3035**	589.2769	588.2929	K	**617.3617**	600.3352	599.3511	6
5	**735.3461**	718.3195	**717.3355**	E	489.2667		471.2562	5
6	**806.3832**	789.3566	**788.3726**	A	360.2241		342.2136	4
7	**893.4152**	876.3886	**875.4046**	S	289.1870		271.1765	3
8	**964.4523**	947.4258	**946.4417**	A	202.1550			2
9				L	131.1179			1

Trypsin digested MS spectrum for **3j**:

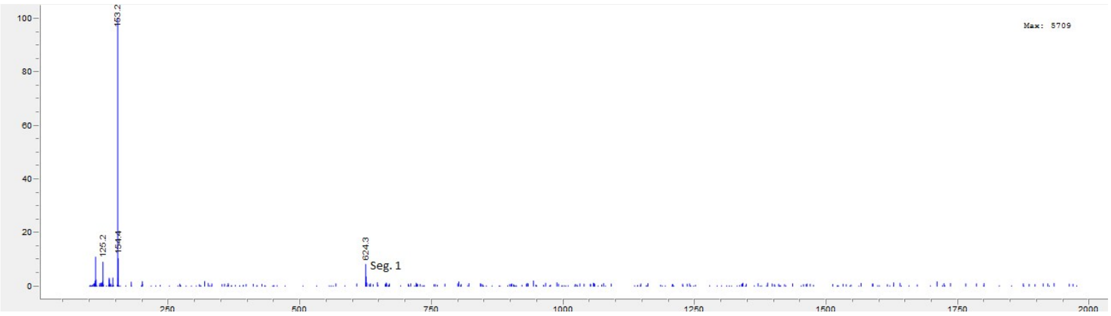

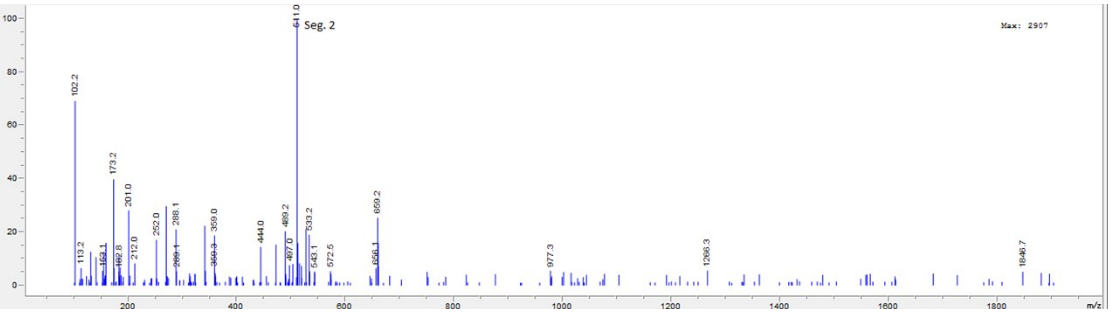

TIC for **3k**:

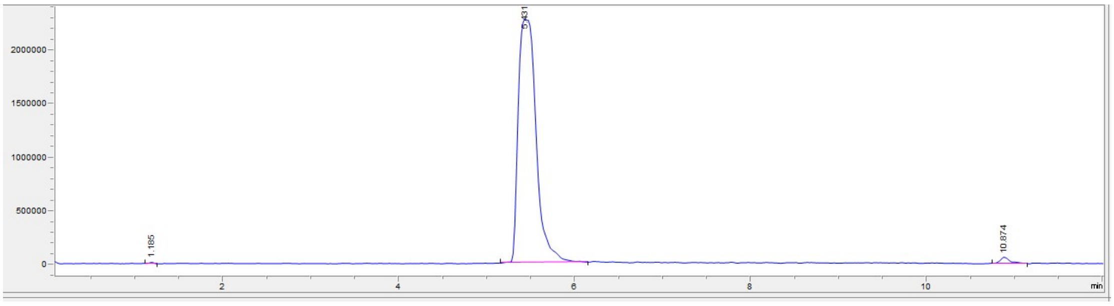

MS Spectrum for **3k**:

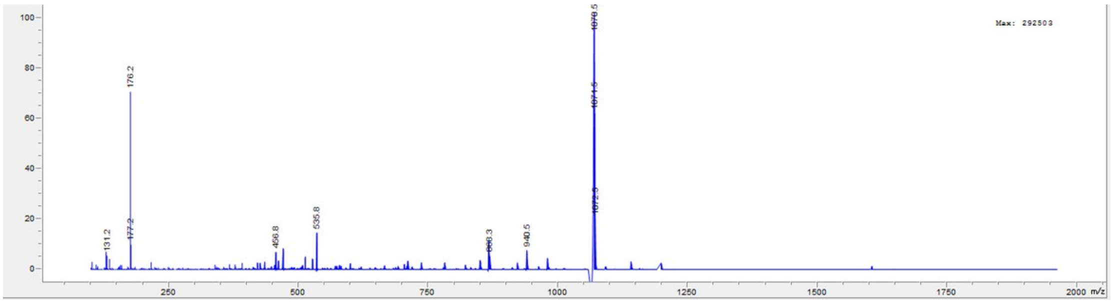

MS MS Spectrum for **3k**:

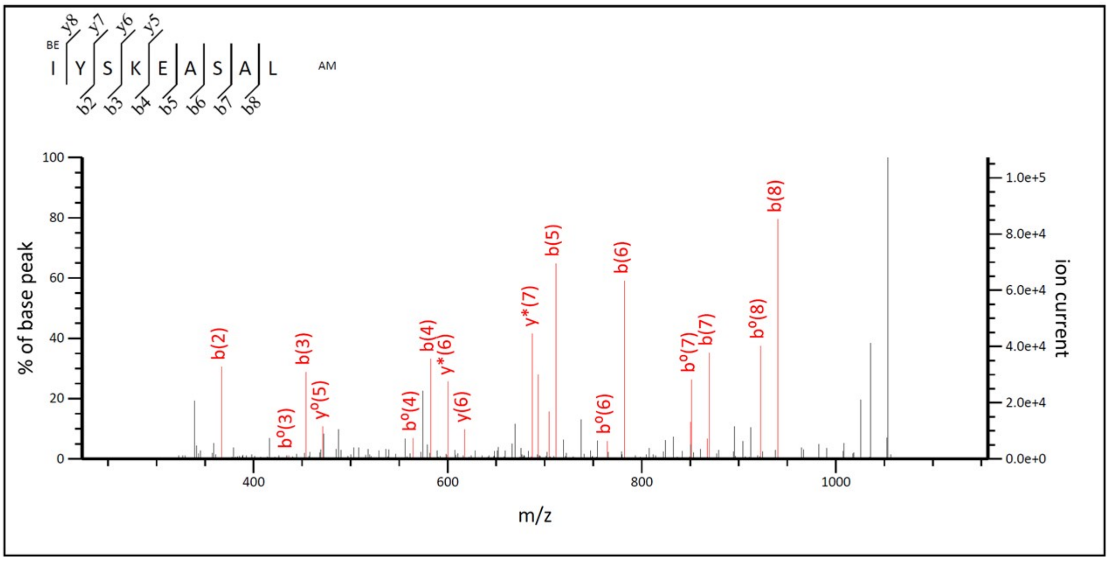

```
Monoisotopic mass of neutral peptide Mr(calc): 1069.5808
Variable modifications:
I1      : Benzyl-I (I)
C-term : Amidated (C-term)
Ions Score: 63  Expect: 4.7e-007   (help)
```

#	b	b*	b⁰	Seq.	y	y*	y⁰	#
1	204.1383			I				9
2	**367.2016**			Y	**867.4571**	**850.4305**	**849.4465**	8
3	**454.2336**		**436.2231**	S	**704.3937**	**687.3672**	**686.3832**	7
4	**582.3286**	565.3021	**564.3180**	K	**617.3617**	**600.3352**	**599.3511**	6
5	**711.3712**	694.3447	**693.3606**	E	489.2667		**471.2562**	5
6	**782.4083**	765.3818	**764.3978**	A	360.2241		342.2136	4
7	**869.4403**	852.4138	**851.4298**	S	289.1870		271.1765	3
8	**940.4775**	923.4509	**922.4669**	A	202.1550			2
9				L	131.1179			1

Trypsin digested MS spectrum for **3k**:

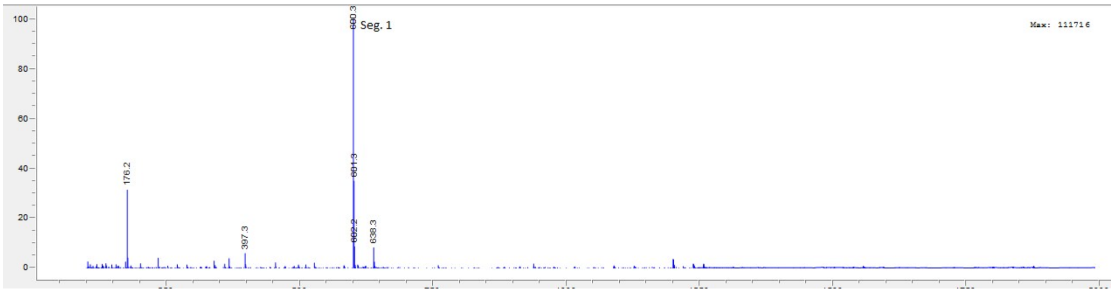

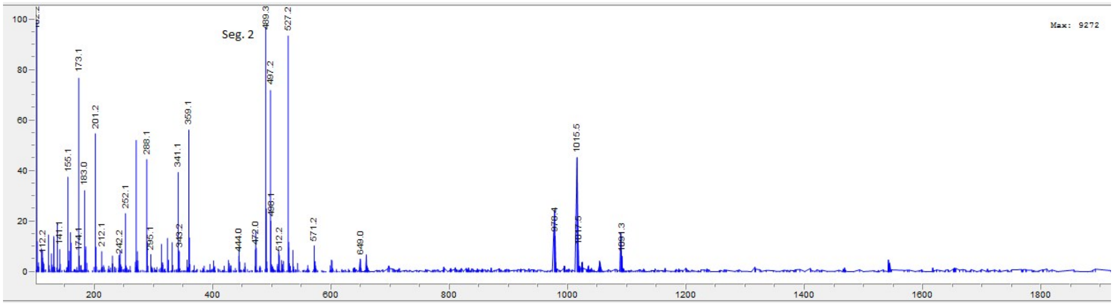

TIC for **3l**:

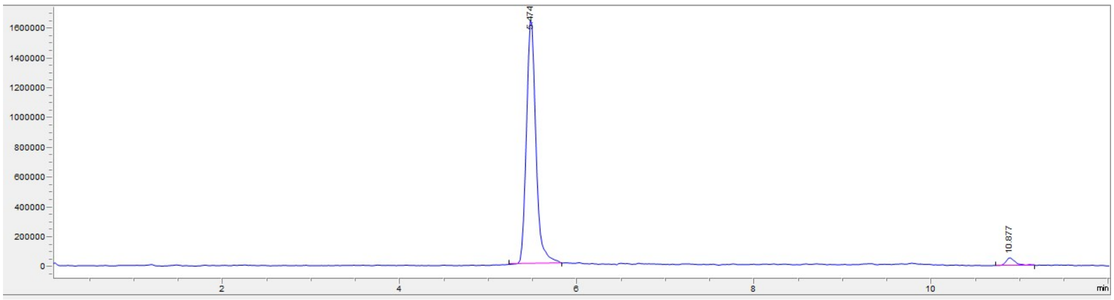

MS Spectrum **3l**:

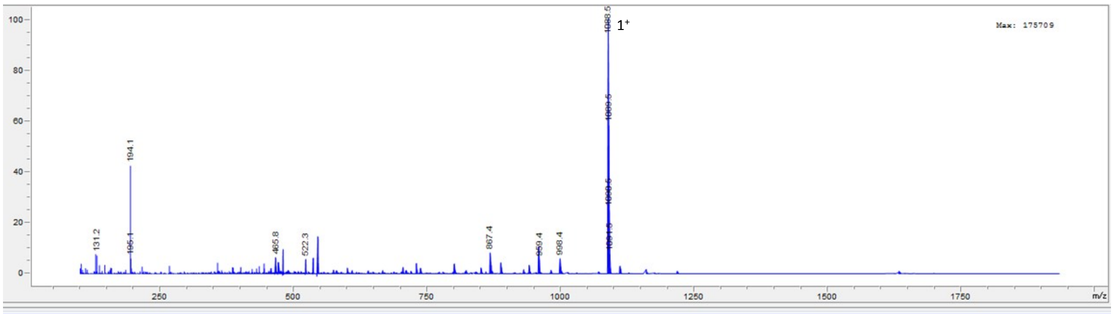

MS MS Spectrum **3l**:

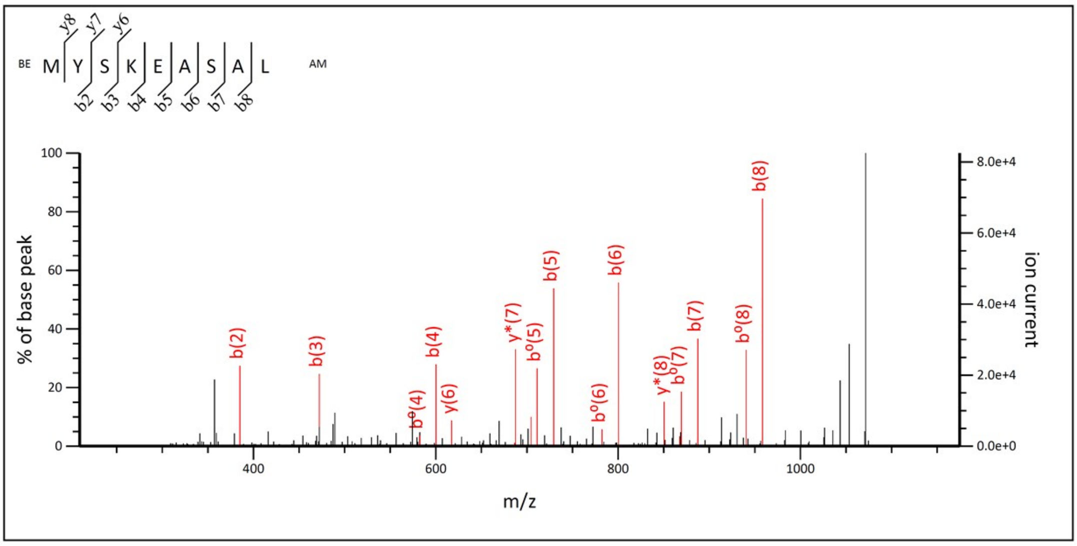

Monoisotopic mass of neutral peptide Mr(calc): 1087.5372
Variable modifications:
N-term : Benzyl (Protein N-term)
C-term : Amidated (Protein C-term)
Ions Score: 69 **Expect:** 1.2e-007 (help)

#	b	b*	b^0	Seq.	y	y*	y^0	#
1	222.0947			M				9
2	**385.1580**			Y	**867.4571**	**850.4305**	849.4465	8
3	**472.1901**		454.1795	S	**704.3937**	**687.3672**	**686.3832**	7
4	**600.2850**	583.2585	**582.2745**	K	**617.3617**	**600.3352**	599.3511	6
5	**729.3276**	712.3011	**711.3171**	E	489.2667		471.2562	5
6	**800.3647**	783.3382	**782.3542**	A	360.2241		342.2136	4
7	**887.3968**	870.3702	**869.3862**	S	289.1870		271.1765	3
8	**958.4339**	941.4073	**940.4233**	A	202.1550			2
9				L	131.1179			1

Trypsin digested MS spectrum for **3l**:

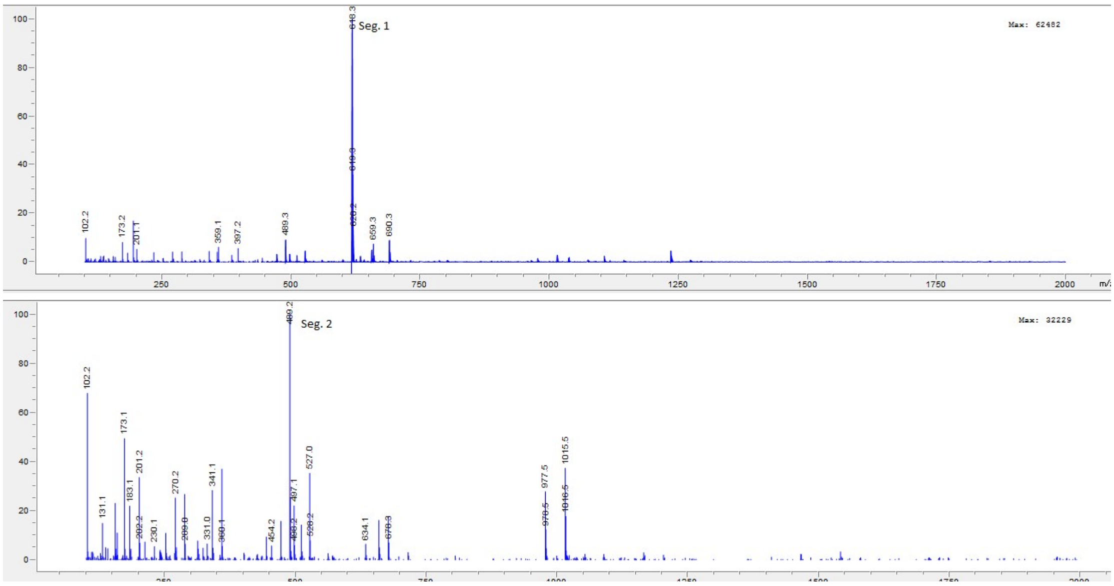

EIC for **3m**:

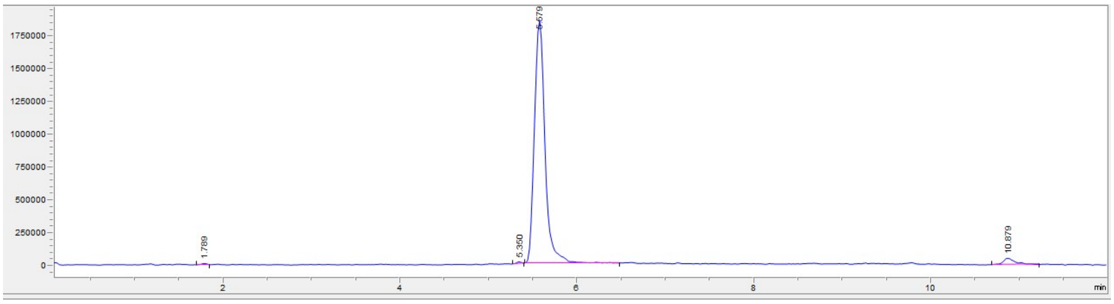

MS Spectrum **3m**:

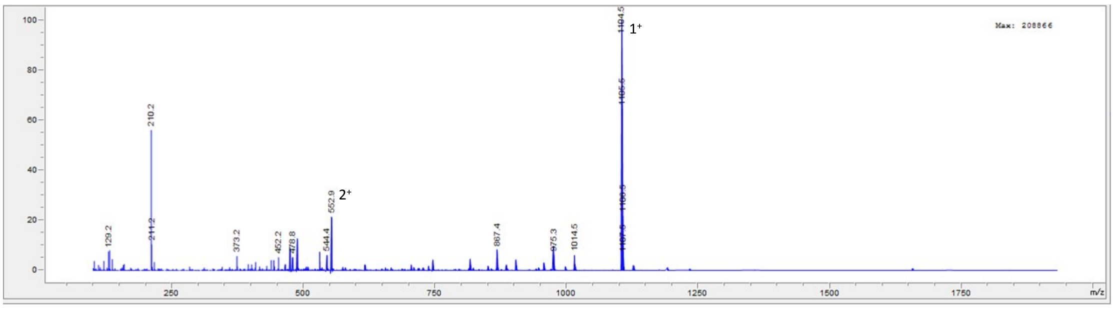

MS MS Spectrum **3m**:

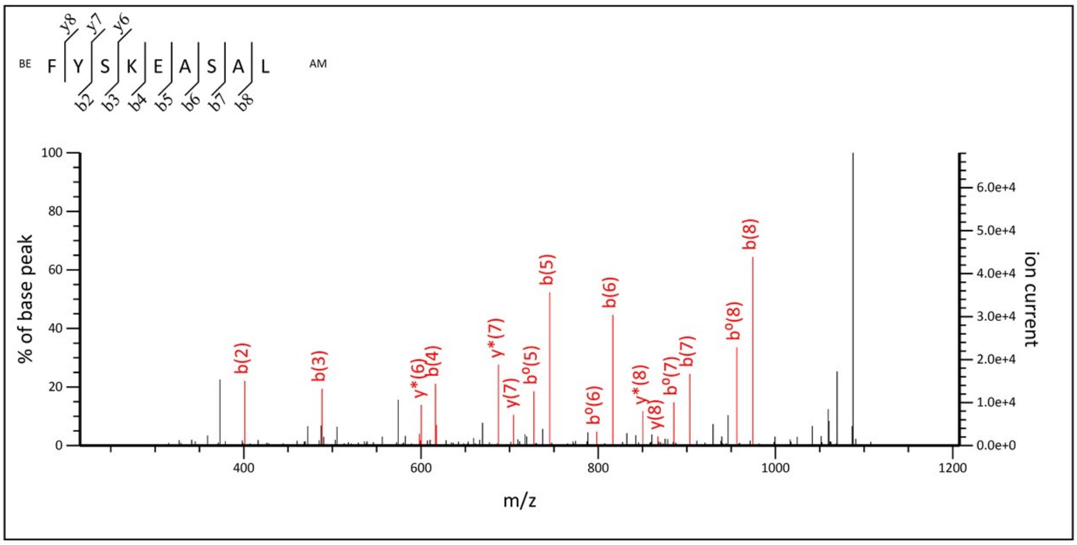

Monoisotopic mass of neutral peptide Mr(calc): 1103.5651
Variable modifications:
N-term : Benzyl (Protein N-term)
C-term : Amidated (Protein C-term)
Ions Score: 59 **Expect:** 1.1e-006 (help)

#	b	b*	b⁰	Seq.	y	y*	y⁰	#
1	238.1226			F				9
2	**401.1860**			Y	**867.4571**	**850.4305**	849.4465	8
3	**488.2180**		470.2074	S	**704.3937**	**687.3672**	**686.3832**	7
4	**616.3130**	599.2864	**598.3024**	K	**617.3617**	**600.3352**	**599.3511**	6
5	**745.3556**	728.3290	**727.3450**	E	489.2667		471.2562	5
6	**816.3927**	799.3661	**798.3821**	A	360.2241		342.2136	4
7	**903.4247**	886.3981	**885.4141**	S	289.1870		271.1765	3
8	**974.4618**	957.4353	**956.4512**	A	202.1550			2
9				L	131.1179			1

Trypsin digested MS spectrum for **3m**:

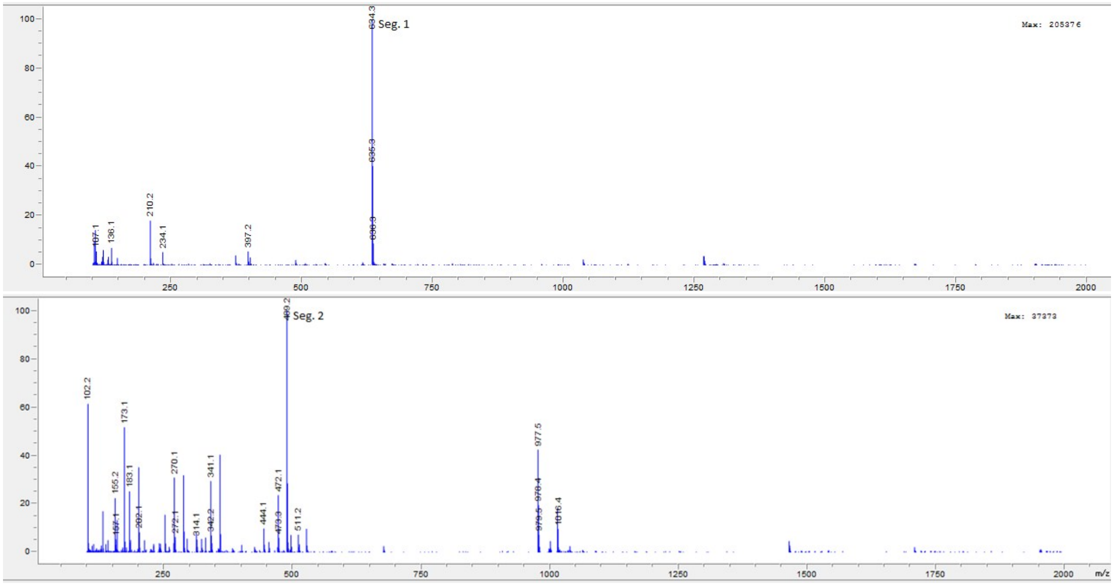

EIC for **3n**:

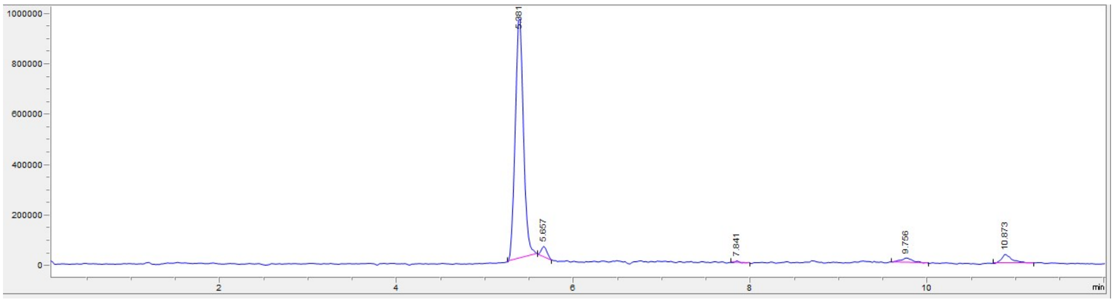

MS Spectrum for **3n**:

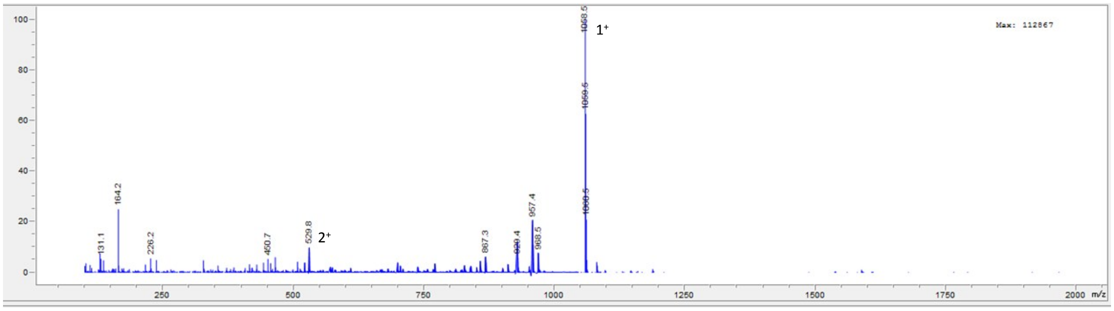

MS MS Spectrum for **3n**:

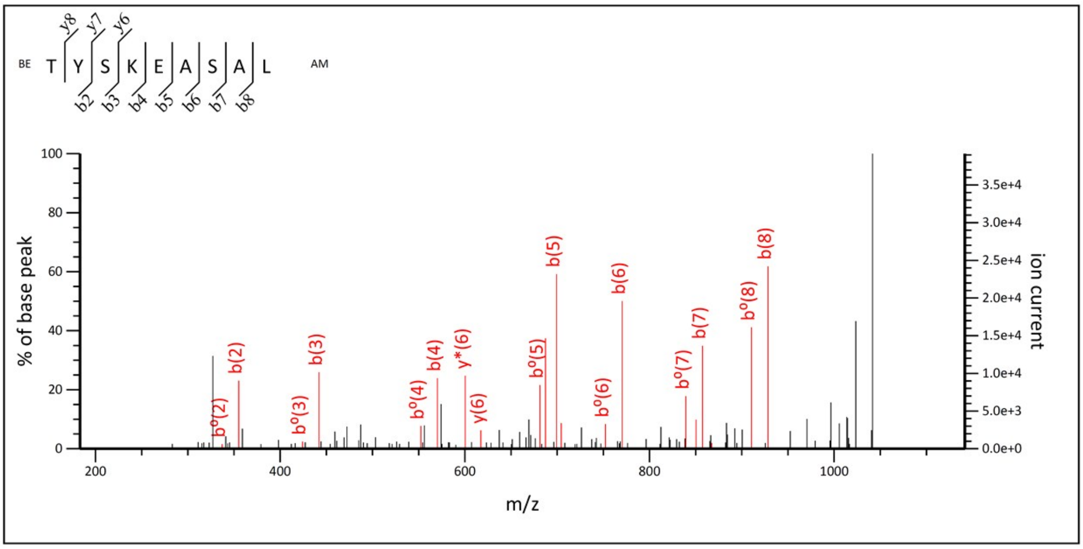

```
Monoisotopic mass of neutral peptide Mr(calc): 1057.5444
Variable modifications:
N-term : Benzyl (Protein N-term)
C-term : Amidated (Protein C-term)
Ions Score: 62   Expect: 6.1e-007    (help)
```

#	b	b*	b^0	Seq.	y	y*	y^0	#
1	192.1019		174.0913	T				9
2	**355.1652**		**337.1547**	Y	**867.4571**	**850.4305**	849.4465	8
3	**442.1973**		**424.1867**	S	**704.3937**	**687.3672**	686.3832	7
4	**570.2922**	553.2657	**552.2817**	K	**617.3617**	**600.3352**	599.3511	6
5	**699.3348**	682.3083	**681.3243**	E	489.2667		471.2562	5
6	**770.3719**	753.3454	**752.3614**	A	360.2241		342.2136	4
7	**857.4040**	840.3774	**839.3934**	S	289.1870		271.1765	3
8	**928.4411**	911.4145	**910.4305**	A	202.1550			2
9				L	131.1179			1

Trypsin digested MS spectrum for **3n**:

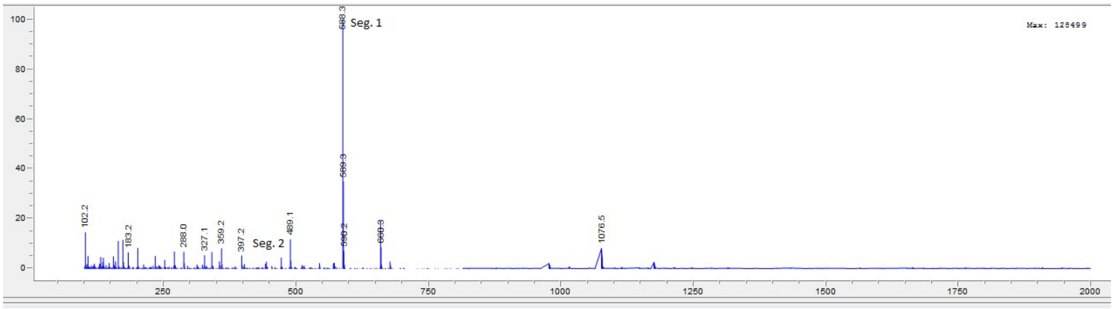

TIC for **3o**:

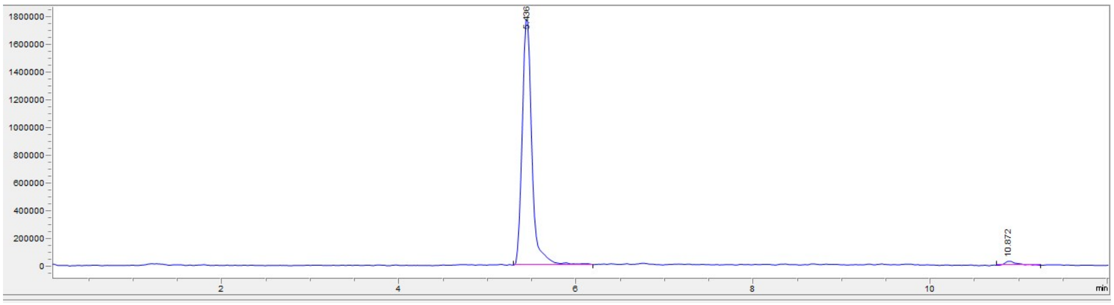

MS Spectrum for **3o**:

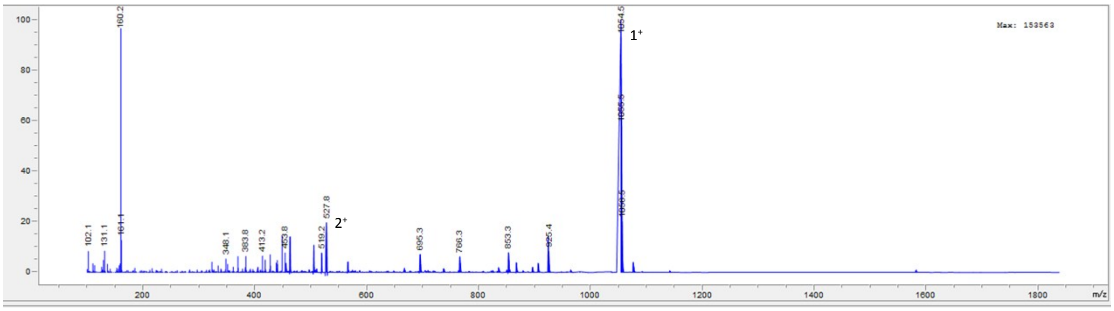

MS MS Spectrum for **3o**:

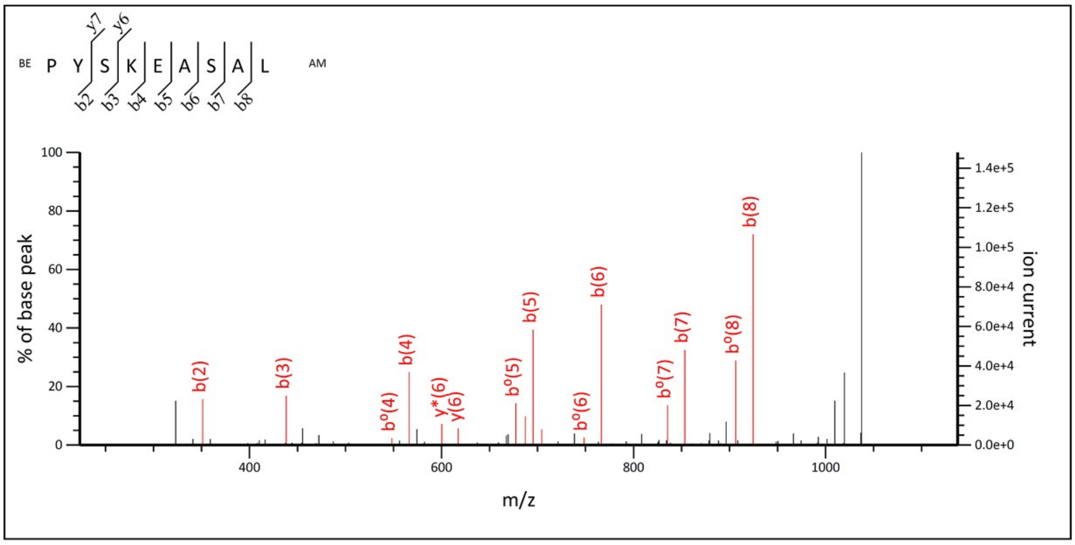


```
Monoisotopic mass of neutral peptide Mr(calc): 1053.5495
Variable modifications:
N-term : Benzyl (Protein N-term)
C-term : Amidated (Protein C-term)
Ions Score: 79   Expect: 1.3e-008   (help)
```

#	b	b*	b^0	Seq.	y	y*	y^0	#
1	188.1070			P				9
2	**351.1703**			Y	867.4571	850.4305	849.4465	8
3	**438.2023**		420.1918	S	**704.3937**	**687.3672**	**686.3832**	7
4	**566.2973**	549.2708	**548.2867**	K	**617.3617**	**600.3352**	599.3511	6
5	**695.3399**	678.3134	**677.3293**	E	489.2667		471.2562	5
6	**766.3770**	749.3505	**748.3665**	A	360.2241		342.2136	4
7	**853.4090**	836.3825	**835.3985**	S	289.1870		271.1765	3
8	**924.4462**	907.4196	**906.4356**	A	202.1550			2
9				L	131.1179			1

Trypsin digested MS spectrum for **3o**:

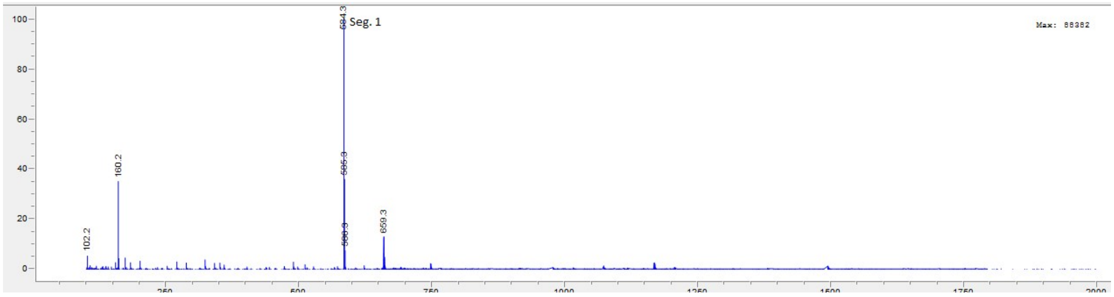

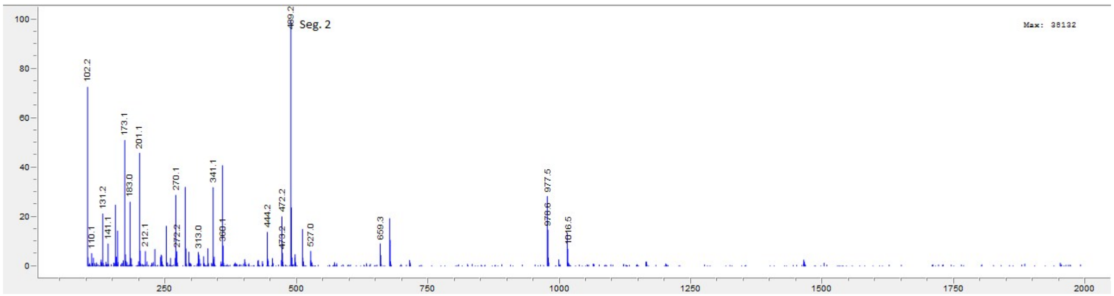

TIC for **3p**:

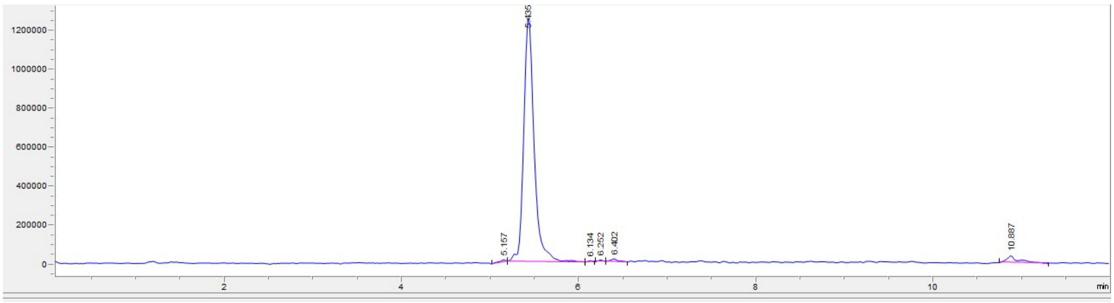

MS Spectrum for **3p**:

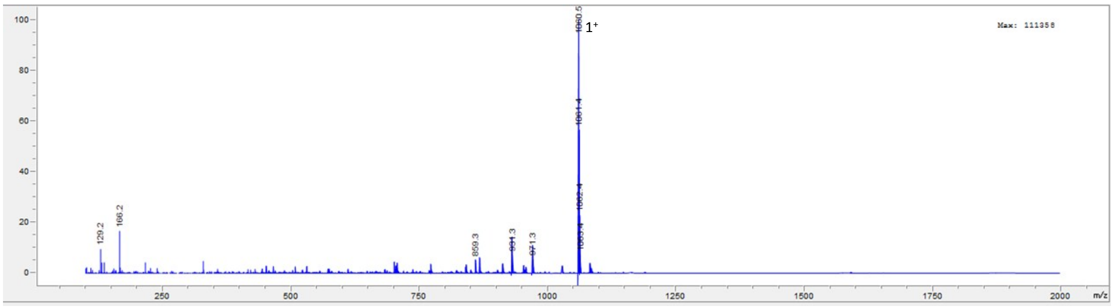

MS MS Spectrum for **3p**:

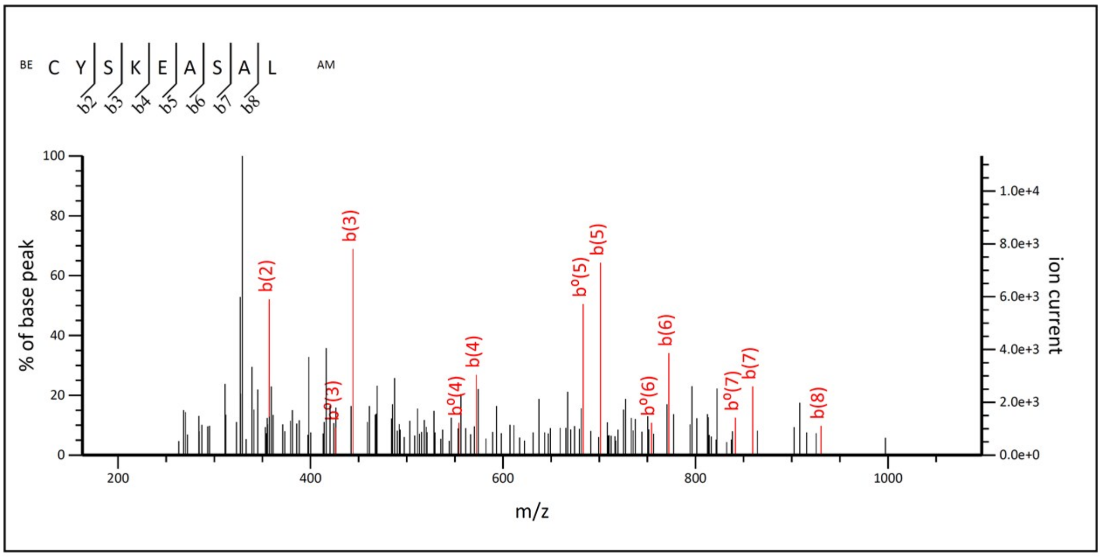

Monoisotopic mass of neutral peptide Mr(calc): 1059.5059
Variable modifications:
N-term : Benzyl (Protein N-term)
C-term : Amidated (Protein C-term)
Ions Score: 36 **Expect:** 0.00024 (help)

#	b	b++	b*	b*++	b0	b0++	Seq.	y	y++	y*	y*++	y0	y0++	#
1	194.0634	97.5353					C							9
2	**357.1267**	179.0670					Y	867.4571	434.2322	850.4305	425.7189	849.4465	425.2269	8
3	**444.1588**	222.5830			**426.1482**	213.5777	S	704.3937	352.7005	687.3672	344.1872	686.3832	343.6952	7
4	**572.2537**	286.6305	555.2272	278.1172	**554.2432**	277.6252	K	617.3617	309.1845	600.3352	300.6712	599.3511	300.1792	6
5	**701.2963**	351.1518	684.2698	342.6385	**683.2858**	342.1465	E	489.2667	245.1370			471.2562	236.1317	5
6	**772.3334**	386.6704	755.3069	378.1571	**754.3229**	377.6651	A	360.2241	180.6157			342.2136	171.6104	4
7	**859.3655**	430.1864	842.3389	421.6731	**841.3549**	421.1811	S	289.1870	145.0972			271.1765	136.0919	3
8	**930.4026**	465.7049	913.3760	457.1917	912.3920	456.6996	A	202.1550	101.5811					2
9							L	131.1179	66.0626					1

Trypsin digested MS spectrum for **3p**:

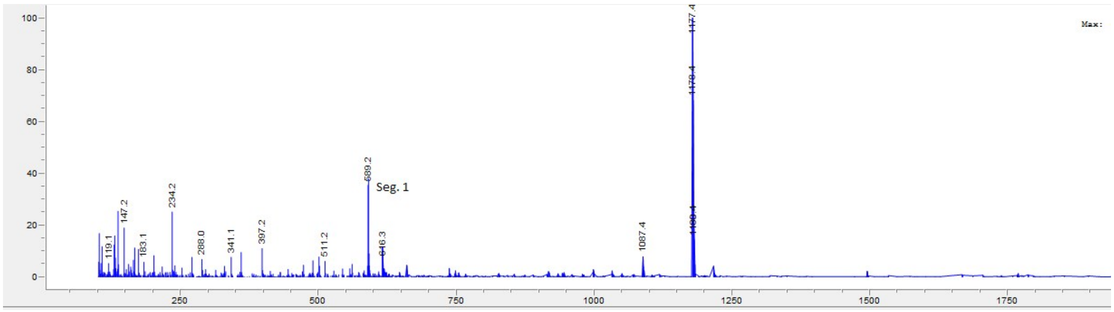

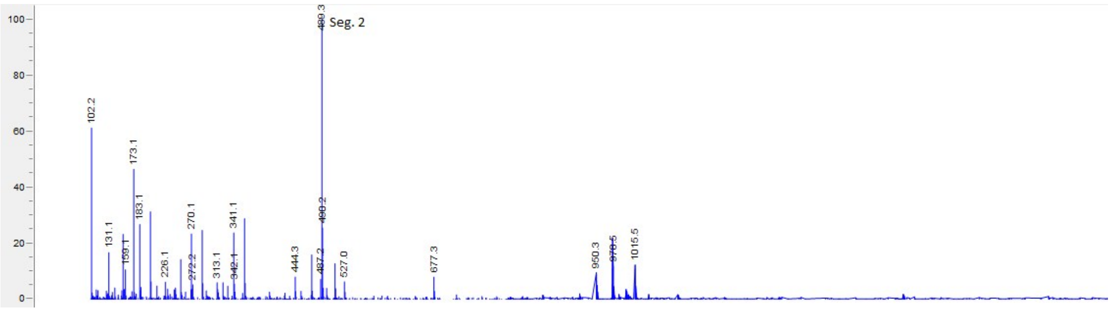

TIC for **3q**:

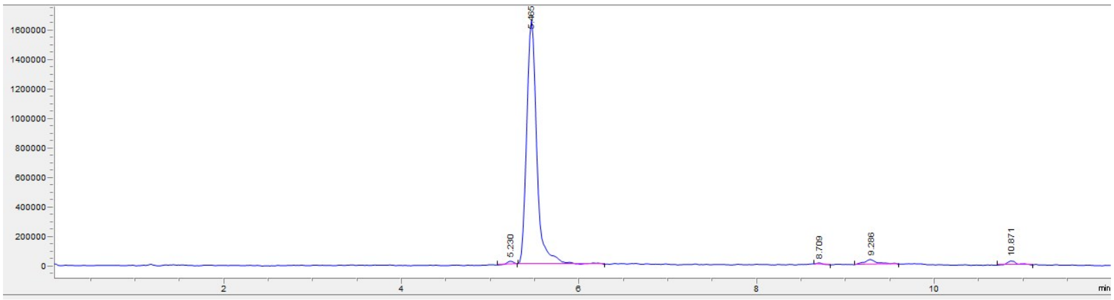

MS Spectrum for **3q**:

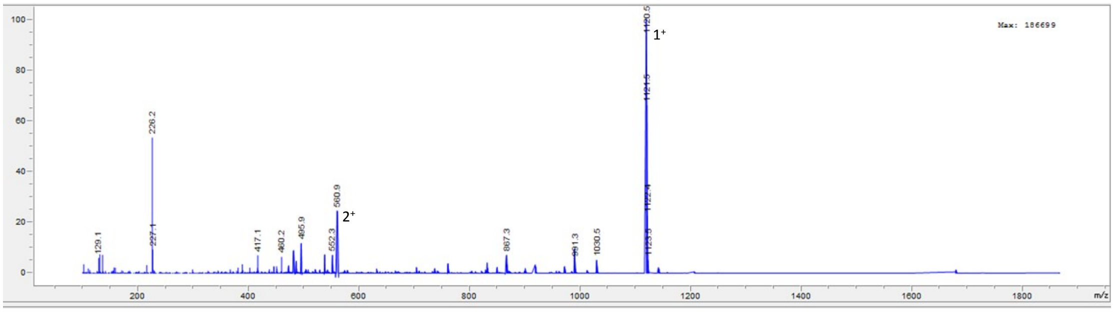

MS MS Spectrum for **3q**:

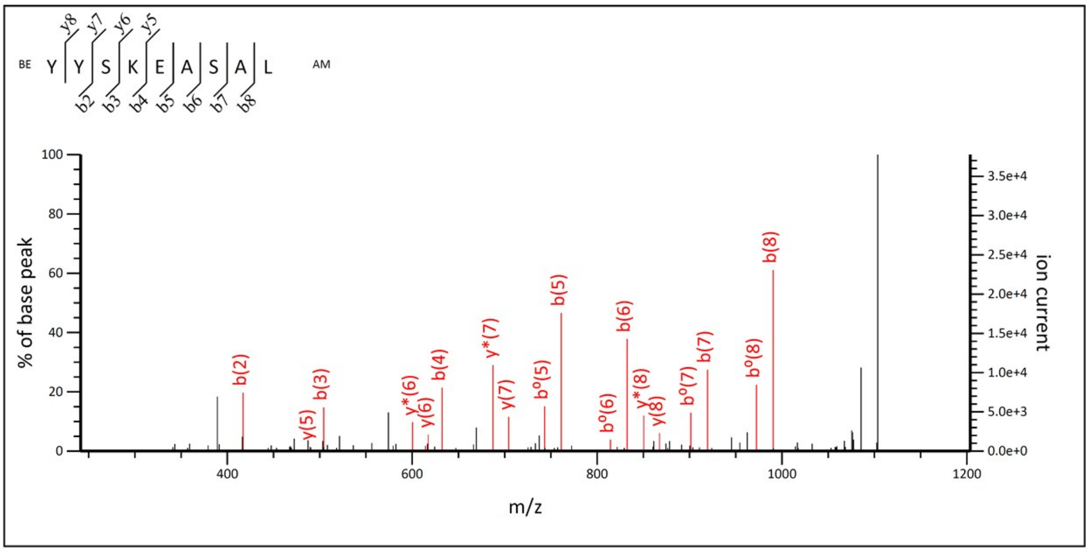

Monoisotopic mass of neutral peptide Mr(calc): 1119.5600
Variable modifications:
N-term : Benzyl (Protein N-term)
C-term : Amidated (Protein C-term)
Ions Score: 64 **Expect:** 4e-007 (help)

#	b	b*	b⁰	Seq.	y	y*	y⁰	#
1	254.1176			Y				9
2	**417.1809**			Y	**867.4571**	**850.4305**	849.4465	8
3	**504.2129**		486.2023	S	**704.3937**	**687.3672**	686.3832	7
4	**632.3079**	615.2813	**614.2973**	K	**617.3617**	**600.3352**	599.3511	6
5	**761.3505**	744.3239	**743.3399**	E	**489.2667**		471.2562	5
6	**832.3876**	815.3610	**814.3770**	A	360.2241		342.2136	4
7	**919.4196**	902.3931	**901.4090**	S	289.1870		271.1765	3
8	**990.4567**	973.4302	**972.4462**	A	202.1550			2
9				L	131.1179			1

Trypsin digested MS spectrum for **3q**:

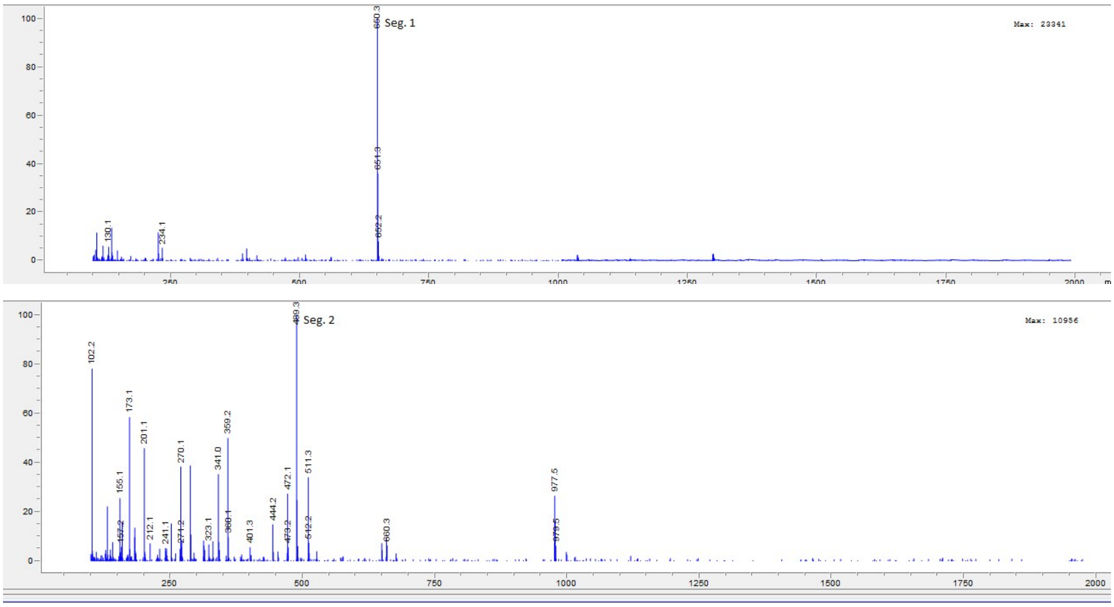

TIC for **3r**:

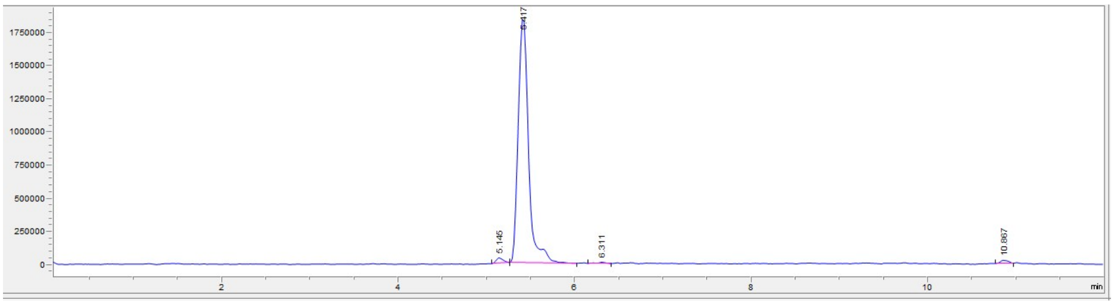

MS Spectrum for **3r**:

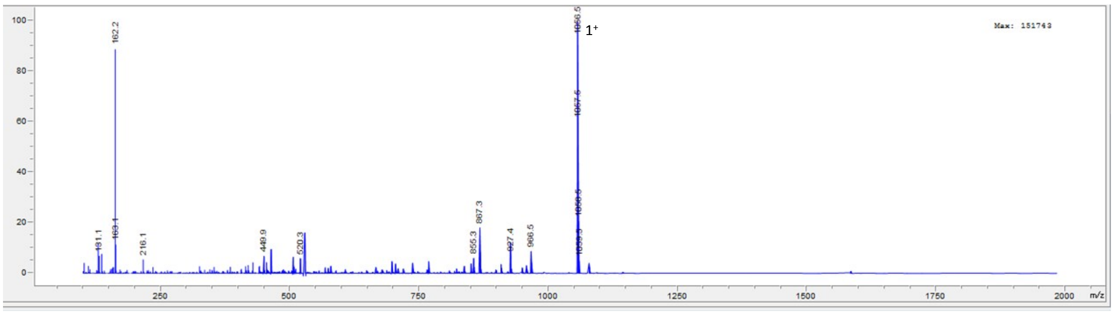

MS MS Spectrum for **3r**:

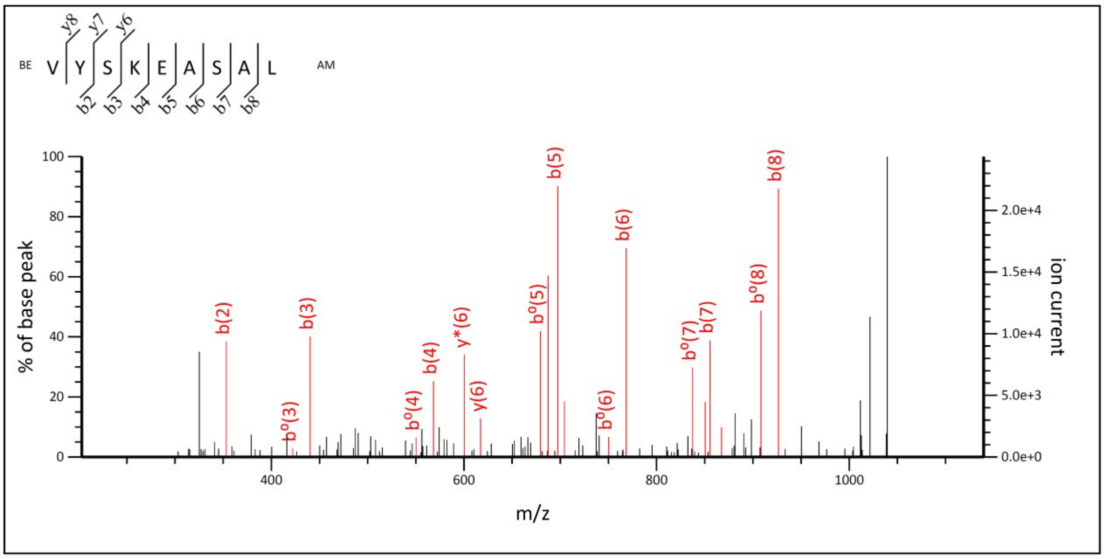

Monoisotopic mass of neutral peptide Mr(calc): 1055.5651
Variable modifications:
N-term : Benzyl (Protein N-term)
C-term : Amidated (Protein C-term)
Ions Score: 69 **Expect:** 1.2e-007 (help)

#	b	b*	b⁰	Seq.	y	y*	y⁰	#
1	190.1226			V				9
2	**353.1860**			Y	**867.4571**	**850.4305**	849.4465	8
3	**440.2180**		**422.2074**	S	**704.3937**	**687.3672**	**686.3832**	7
4	**568.3130**	551.2864	**550.3024**	K	**617.3617**	**600.3352**	599.3511	6
5	**697.3556**	680.3290	**679.3450**	E	489.2667		471.2562	5
6	**768.3927**	751.3661	**750.3821**	A	360.2241		342.2136	4
7	**855.4247**	838.3981	**837.4141**	S	289.1870		271.1765	3
8	**926.4618**	909.4353	**908.4512**	A	202.1550			2
9				L	131.1179			1

Trypsin digested MS spectrum for **3r**:

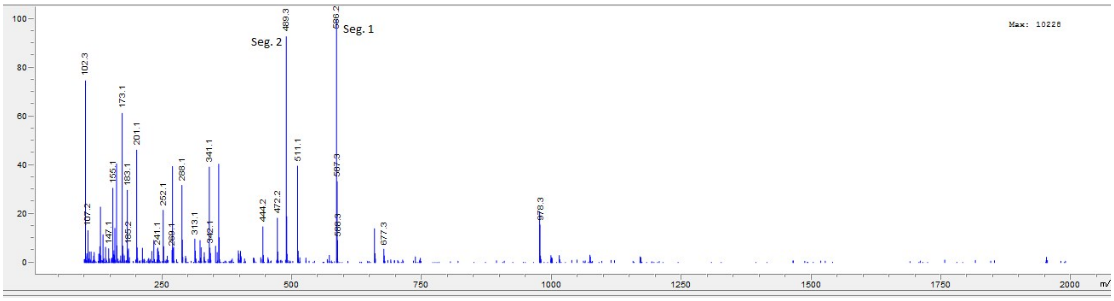

TIC for **3s**:

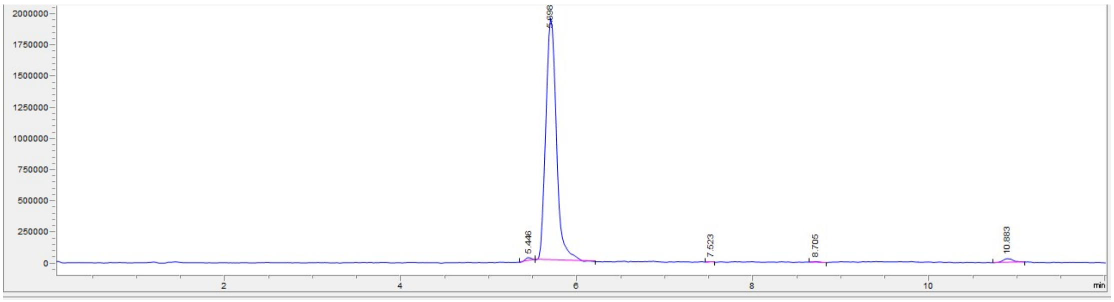

MS Spectrum for **3s**:

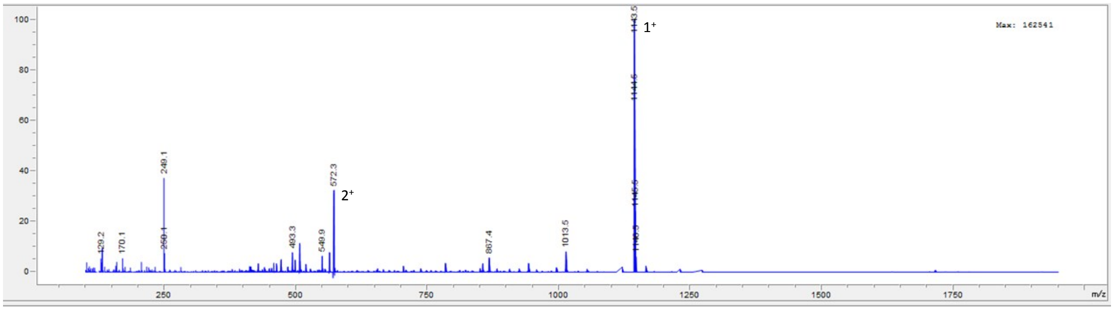

MS MS Spectrum for **3s**:

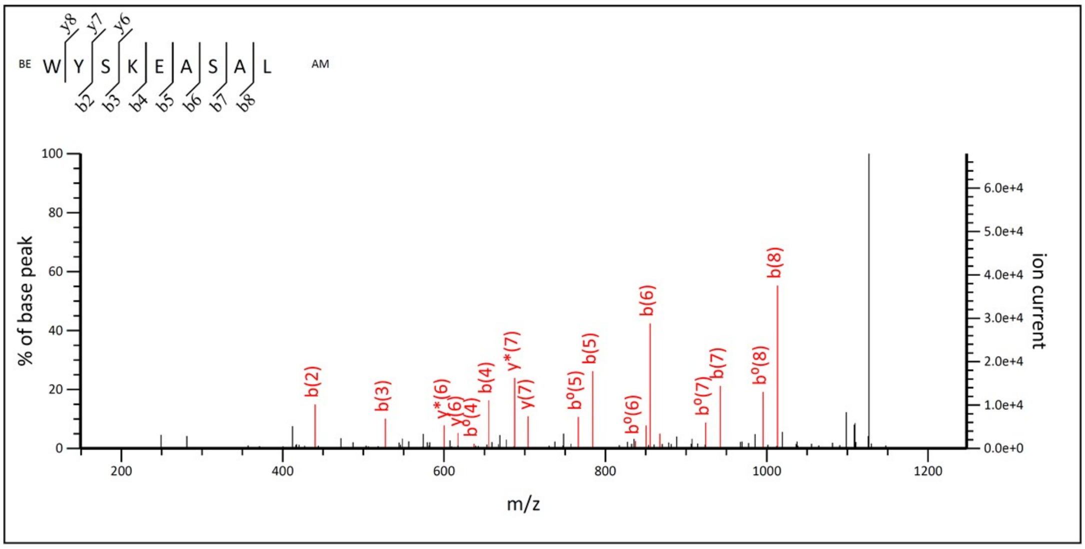

Monoisotopic mass of neutral peptide Mr(calc): 1142.5760
Variable modifications:
N-term : Benzyl (Protein N-term)
C-term : Amidated (Protein C-term)
Ions Score: 58 **Expect:** 1.5e-006 (help)

#	b	b*	b^0	Seq.	y	y*	y^0	#
1	277.1335			W				9
2	**440.1969**			Y	**867.4571**	**850.4305**	849.4465	8
3	**527.2289**		509.2183	S	**704.3937**	**687.3672**	**686.3832**	7
4	**655.3239**	638.2973	**637.3133**	K	**617.3617**	**600.3352**	599.3511	6
5	**784.3665**	767.3399	**766.3559**	E	489.2667		471.2562	5
6	**855.4036**	838.3770	**837.3930**	A	360.2241		342.2136	4
7	**942.4356**	925.4090	**924.4250**	S	289.1870		271.1765	3
8	**1013.4727**	996.4462	**995.4621**	A	202.1550			2
9				L	131.1179			1

Trypsin digested MS spectrum for **3s**:

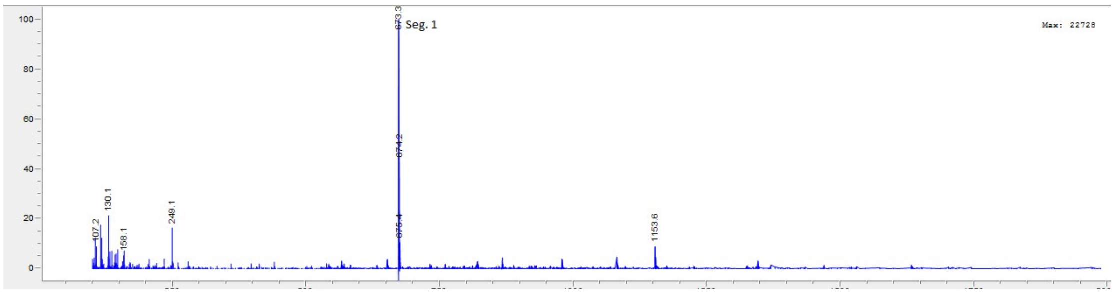

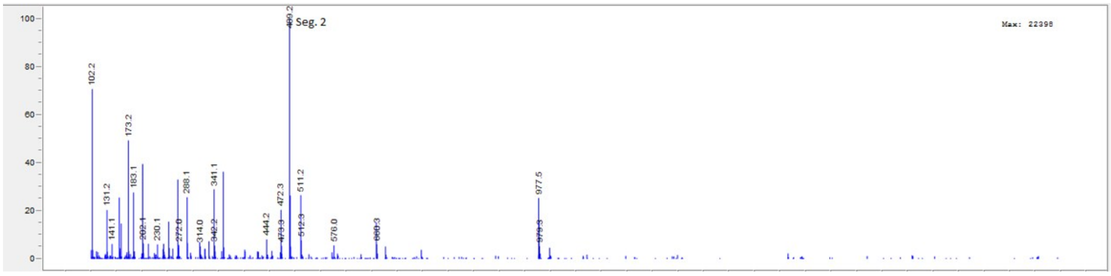

TIC for **3t**:

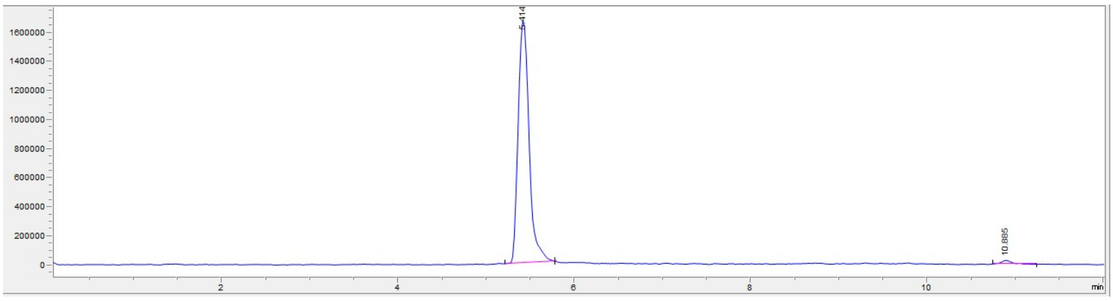

MS Spectrum for **3t**:

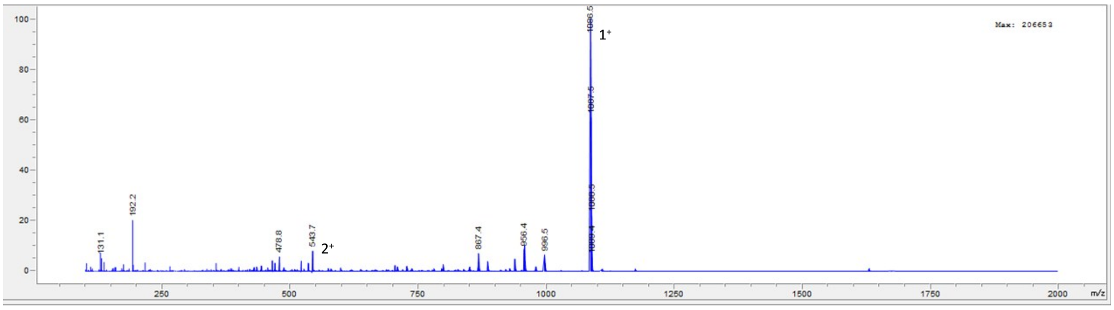

MS MS Spectrum for **3t**:

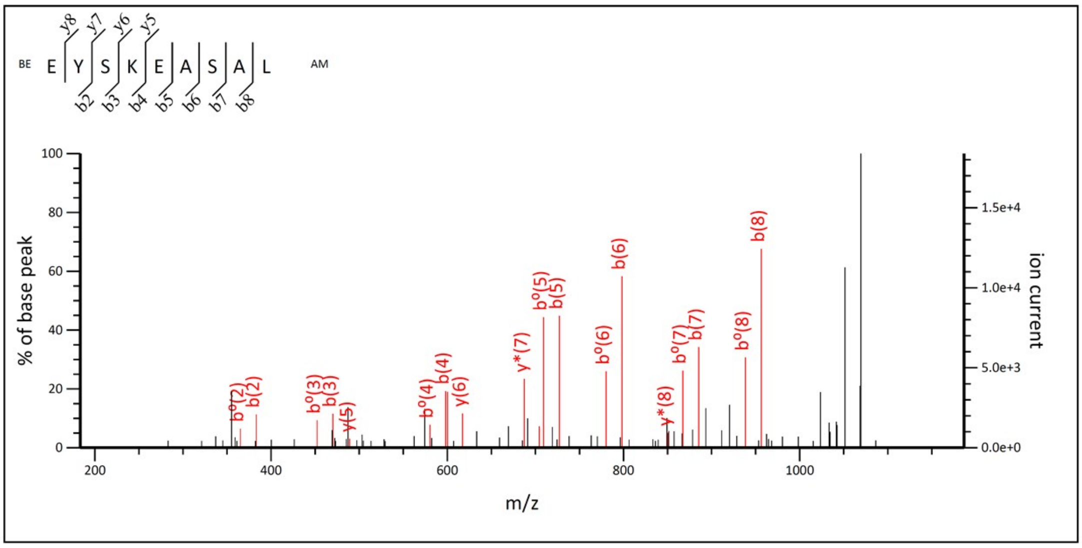

Monoisotopic mass of neutral peptide Mr(calc): 1085.5393
Variable modifications:
N-term : Benzyl (Protein N-term)
C-term : Amidated (Protein C-term)
Ions Score: 62 **Expect:** 6.4e-007 (help)

#	b	b*	b^0	Seq.	y	y*	y^0	#
1	220.0968		202.0863	E				9
2	**383.1601**		**365.1496**	Y	867.4571	**850.4305**	849.4465	8
3	**470.1922**		**452.1816**	S	**704.3937**	**687.3672**	686.3832	7
4	**598.2871**	581.2606	**580.2766**	K	**617.3617**	**600.3352**	599.3511	6
5	**727.3297**	710.3032	**709.3192**	E	**489.2667**		471.2562	5
6	**798.3668**	781.3403	**780.3563**	A	360.2241		342.2136	4
7	**885.3989**	868.3723	**867.3883**	S	289.1870		271.1765	3
8	**956.4360**	939.4094	**938.4254**	A	202.1550			2
9				L	131.1179			1

Trypsin digested MS spectrum for **3t**:

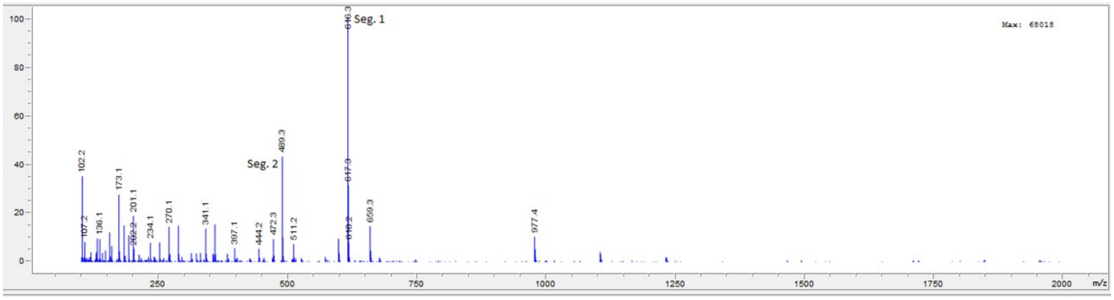

TIC for **3u**:

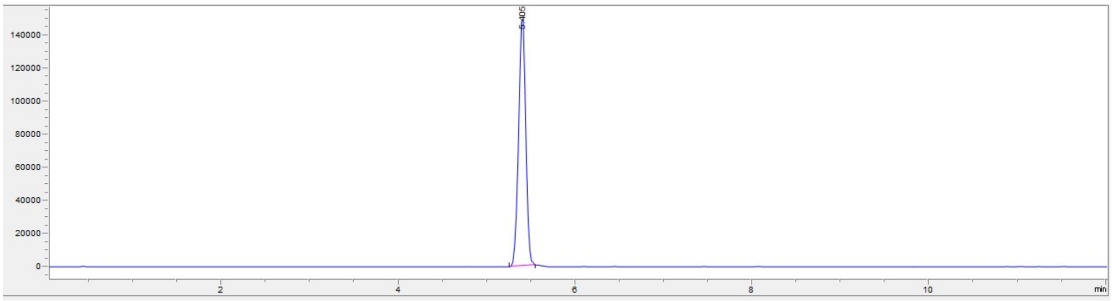

MS Spectrum for **3u**:

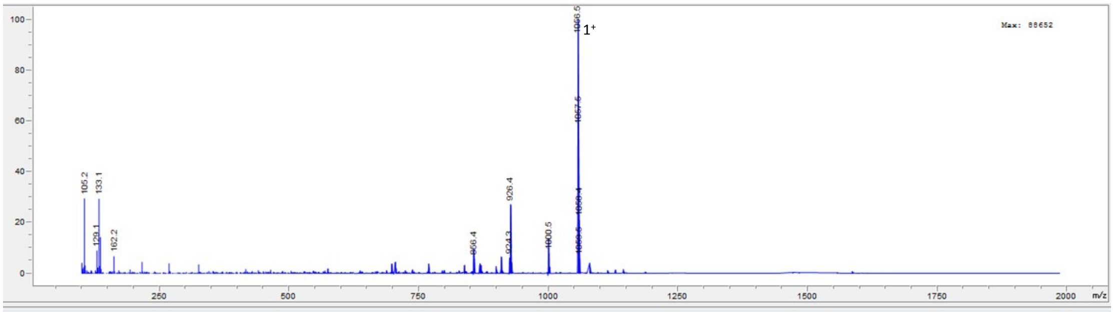

MS/MS Spectrum for **3u**:

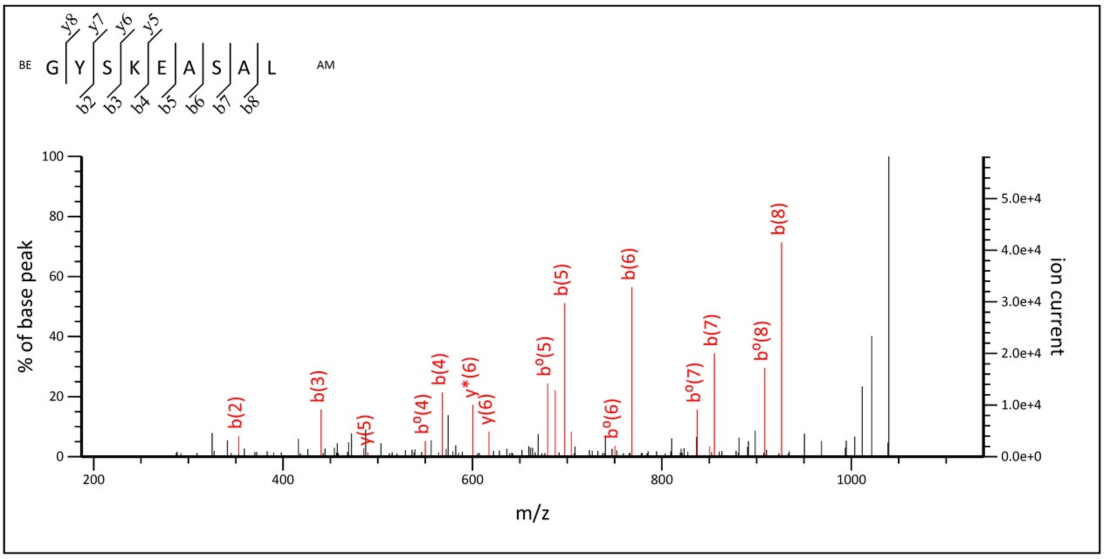

Monoisotopic mass of neutral peptide Mr(calc): 1055.5287
Variable modifications:
N-term : Benzyl-P-Acetyl (N-term)
C-term : Amidated (Protein C-term)
Ions Score: 62 **Expect:** 5.6e-007 (help)

#	b	b*	b^0	Seq.	y	y*	y^0	#
1	190.0863			G				9
2	**353.1496**			Y	867.4571	**850.4305**	849.4465	8
3	**440.1816**		422.1710	S	**704.3937**	**687.3672**	686.3832	7
4	**568.2766**	551.2500	**550.2660**	K	**617.3617**	**600.3352**	599.3511	6
5	**697.3192**	680.2926	**679.3086**	E	**489.2667**		471.2562	5
6	**768.3563**	751.3297	**750.3457**	A	360.2241		342.2136	4
7	**855.3883**	838.3618	**837.3777**	S	289.1870		271.1765	3
8	**926.4254**	909.3989	**908.4149**	A	202.1550			2
9				L	131.1179			1

Trypsin digested MS spectrum for **3u**:

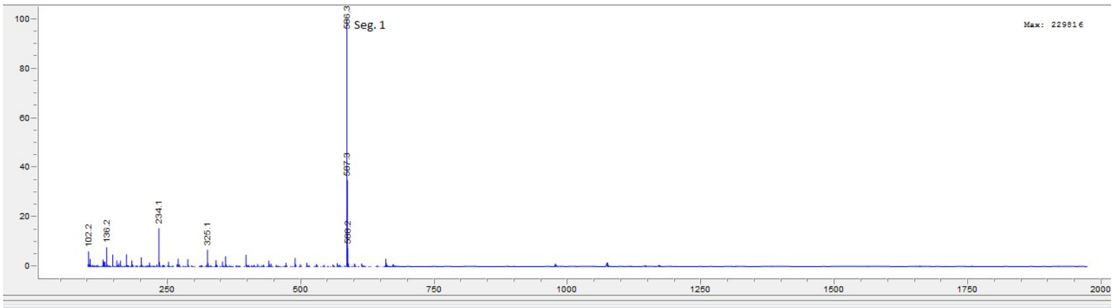

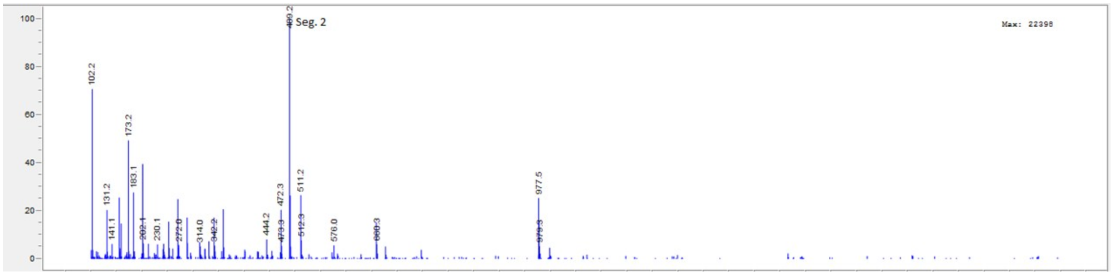

TIC for **3v**:

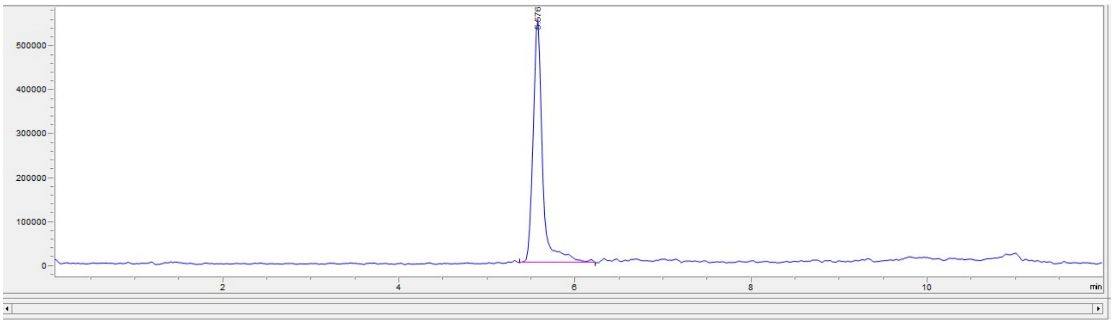

MS Spectrum for **3v**:

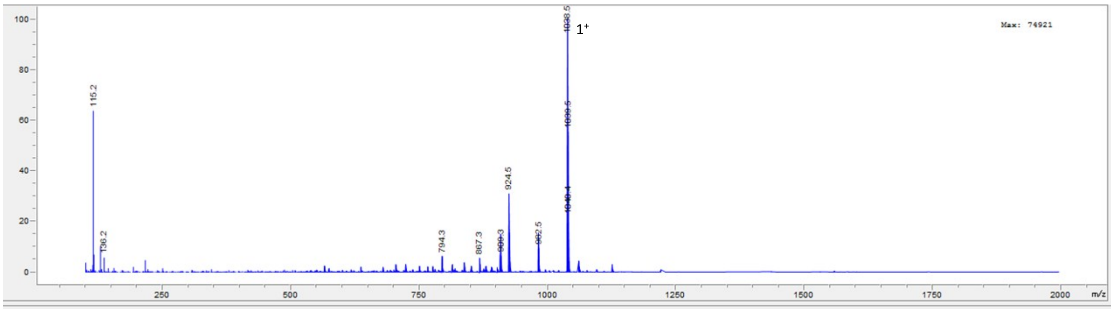

MS MS Spectrum for **3v**:

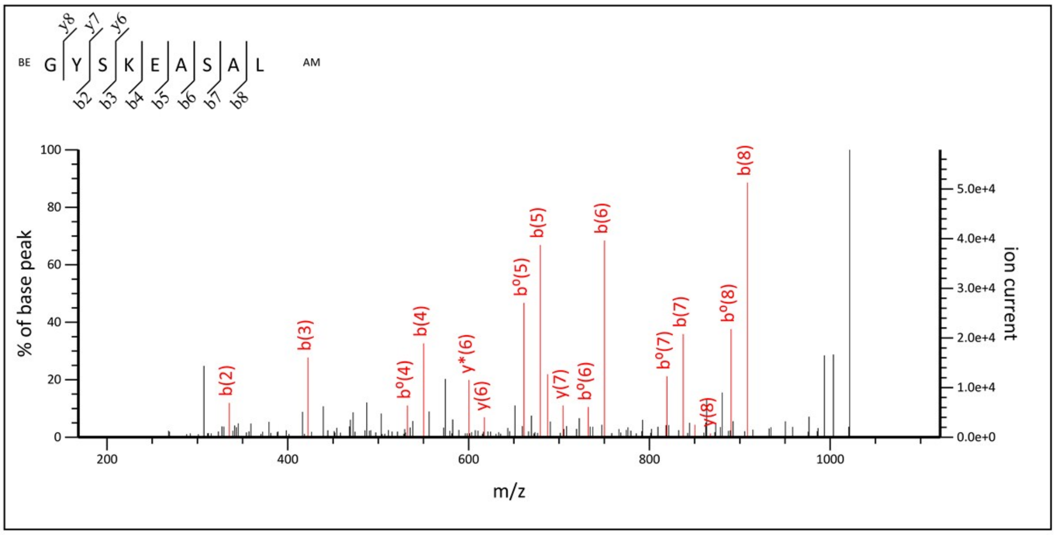

Monoisotopic mass of neutral peptide Mr(calc): 1037.5182
Variable modifications:
N-term : Benzyl-P-Ethylnyl (N-term)
C-term : Amidated (C-term)
Ions Score: 62 **Expect:** 6.2e-007 (help)

#	b	b*	b⁰	Seq.	y	y*	y⁰	#
1	172.0757			G				9
2	**335.1390**			Y	**867.4571**	**850.4305**	849.4465	8
3	**422.1710**		404.1605	S	**704.3937**	**687.3672**	686.3832	7
4	**550.2660**	533.2395	**532.2554**	K	**617.3617**	**600.3352**	599.3511	6
5	**679.3086**	662.2821	**661.2980**	E	489.2667		471.2562	5
6	**750.3457**	733.3192	**732.3352**	A	360.2241		342.2136	4
7	**837.3777**	820.3512	**819.3672**	S	289.1870		271.1765	3
8	**908.4149**	891.3883	**890.4043**	A	202.1550			2
9				L	131.1179			1

Trypsin digested MS spectrum for **3v**:

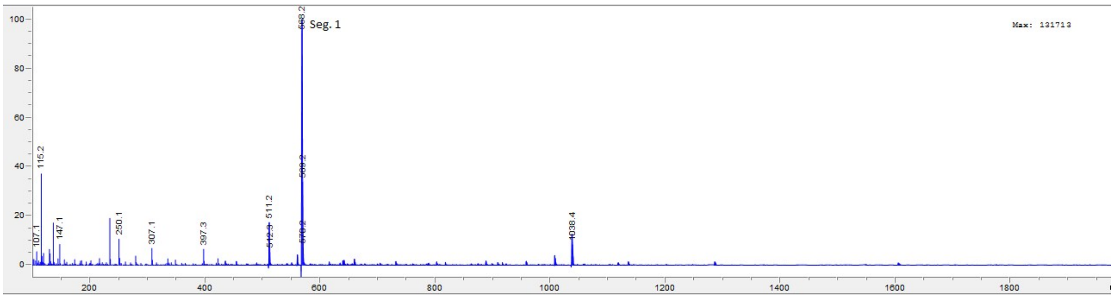

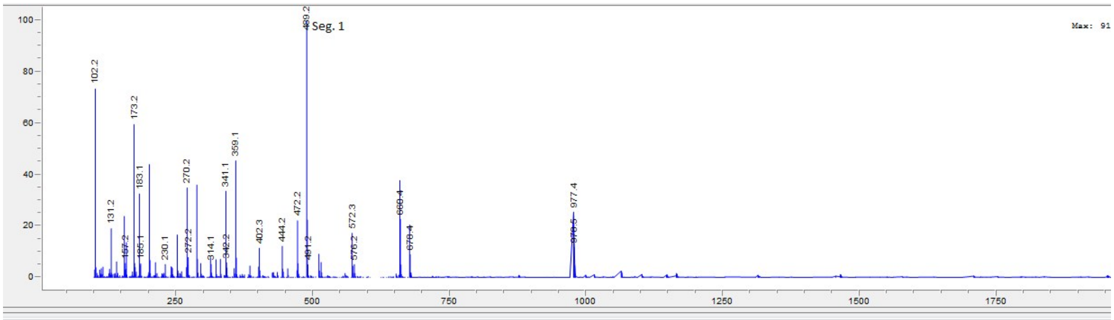

TIC for **3w**:

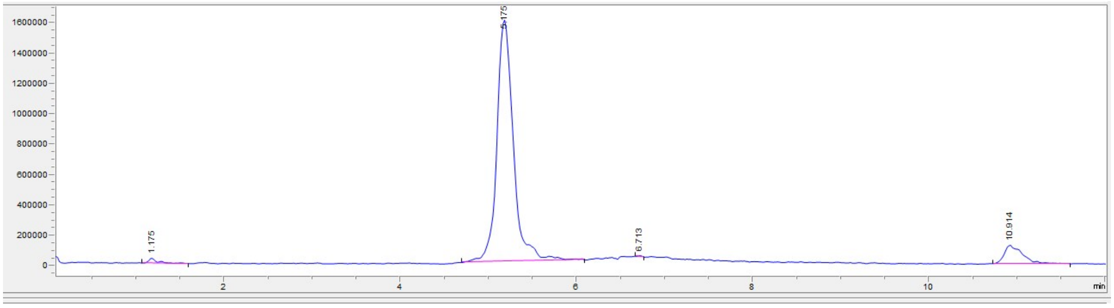

MS Spectrum for **3w**:

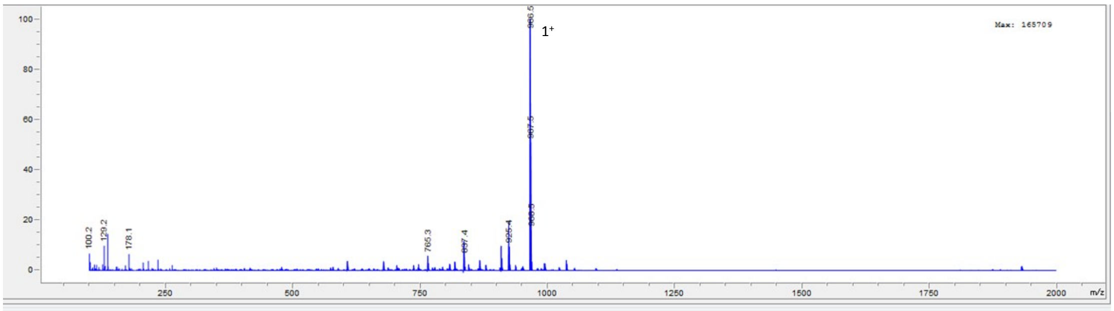

MS MS Spectrum for **3w**:

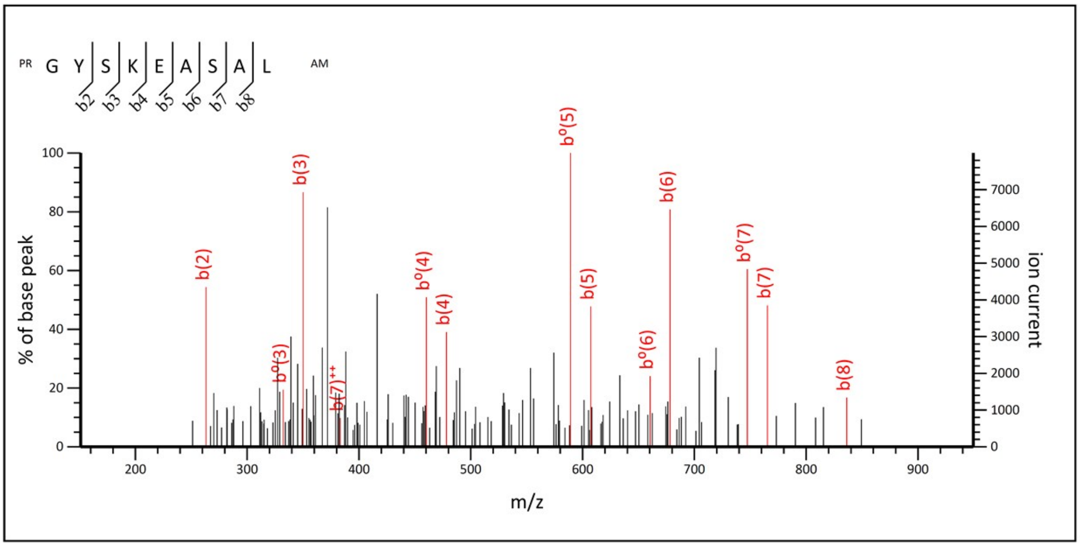

```
Monoisotopic mass of neutral peptide Mr(calc): 965.5182
Variable modifications:
N-term : Propyl (N-term)
C-term : Amidated (Protein C-term)
Ions Score: 69  Expect: 1.3e-007    (help)
```

#	b	b^{++}	b*	b^{*++}	b^{0}	b^{0++}	Seq.	y	y^{++}	y*	y^{*++}	y^{0}	y^{0++}	#
1	100.0757	50.5415					G							9
2	**263.1390**	132.0731					Y	867.4571	434.2322	850.4305	425.7189	849.4465	425.2269	8
3	**350.1710**	175.5892			**332.1605**	166.5839	S	704.3937	352.7005	687.3672	344.1872	686.3832	343.6952	7
4	**478.2660**	239.6366	461.2395	231.1234	**460.2554**	230.6314	K	617.3617	309.1845	600.3352	300.6712	599.3511	300.1792	6
5	**607.3086**	304.1579	590.2821	295.6447	**589.2980**	295.1527	E	489.2667	245.1370			471.2562	236.1317	5
6	**678.3457**	339.6765	661.3192	331.1632	**660.3352**	330.6712	A	360.2241	180.6157			342.2136	171.6104	4
7	**765.3777**	**383.1925**	748.3512	374.6792	**747.3672**	374.1872	S	289.1870	145.0972			271.1765	136.0919	3
8	**836.4149**	418.7111	819.3883	410.1978	818.4043	409.7058	A	202.1550	101.5811					2
9							L	131.1179	66.0626					1

Trypsin digested MS spectrum for **3w**:

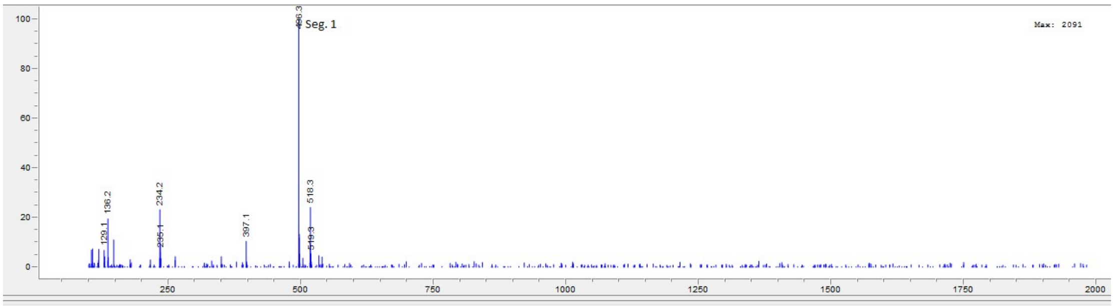

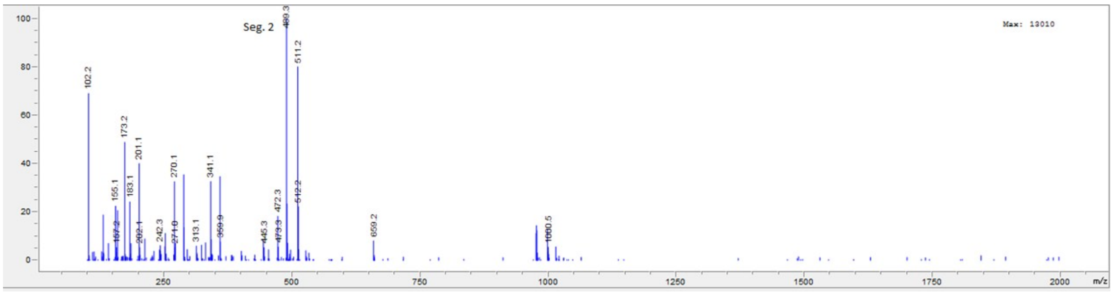

TIC for **3x**:

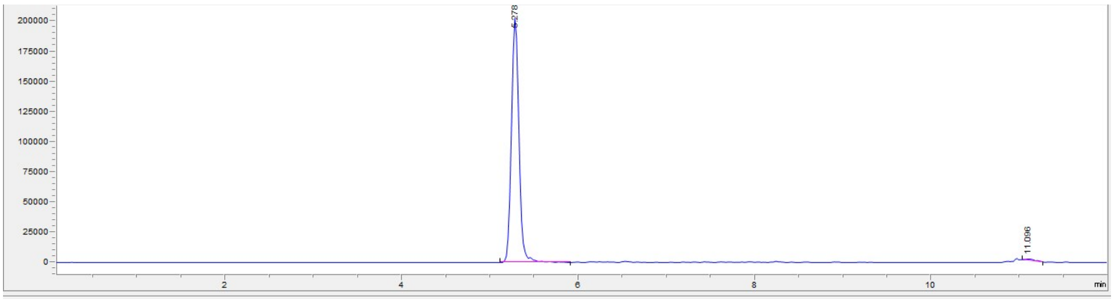

MS Spectrum for **3x**:

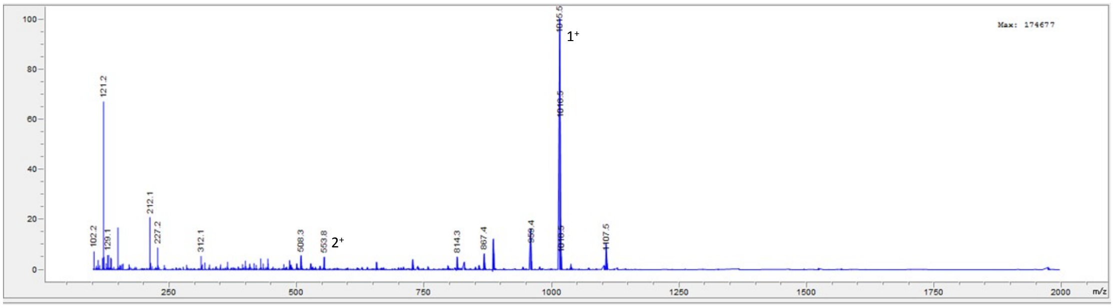

MS MS Spectrum for **3x**:

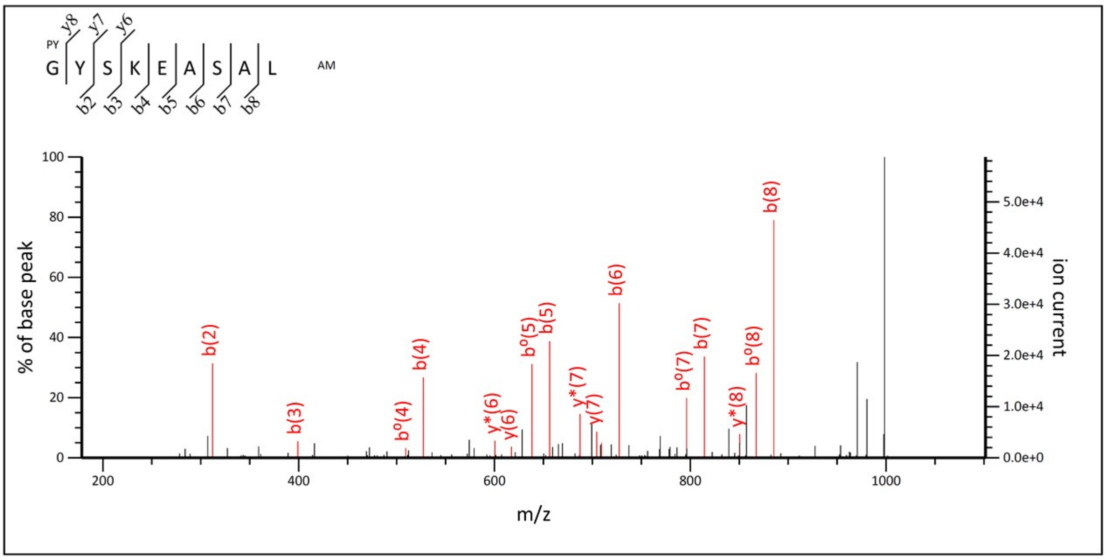

Monoisotopic mass of neutral peptide Mr(calc): 1014.5134
Variable modifications:
G1 : Pyridine-2-yl (G)
C-term : Amidated (C-term)
Ions Score: 78 **Expect:** 1.6e-008 (help)

#	b	b*	b0	Seq.	y	y*	y0	#
1	149.0709			G				9
2	**312.1343**			Y	**867.4571**	**850.4305**	849.4465	8
3	**399.1663**		381.1557	S	**704.3937**	**687.3672**	**686.3832**	7
4	**527.2613**	510.2347	**509.2507**	K	**617.3617**	**600.3352**	599.3511	6
5	**656.3039**	639.2773	**638.2933**	E	489.2667		471.2562	5
6	**727.3410**	710.3144	**709.3304**	A	360.2241		342.2136	4
7	**814.3730**	797.3464	**796.3624**	S	289.1870		271.1765	3
8	**885.4101**	868.3836	**867.3995**	A	202.1550			2
9				L	131.1179			1

Trypsin digested MS spectrum for **3x**:

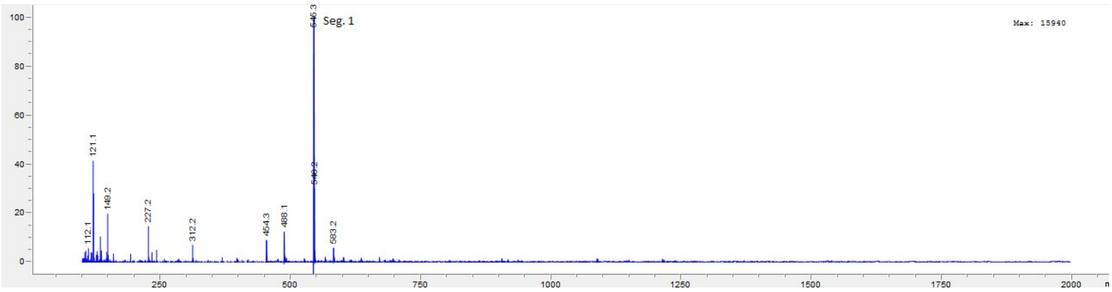

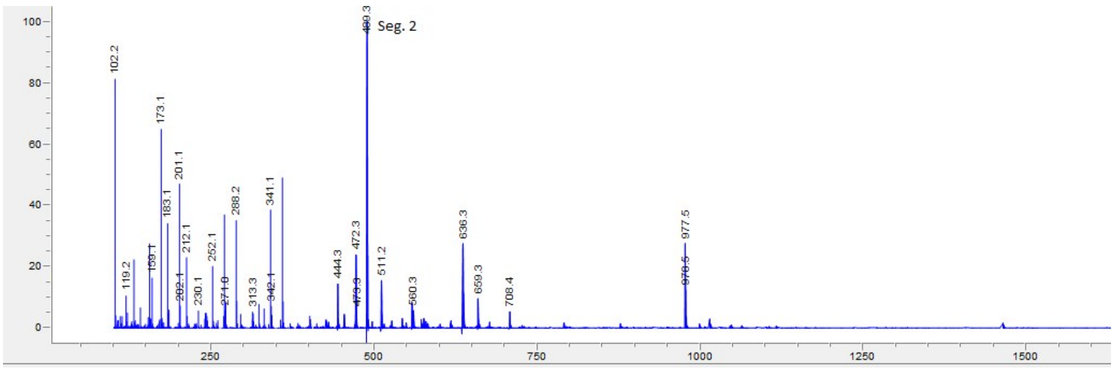

TIC for **3y**:

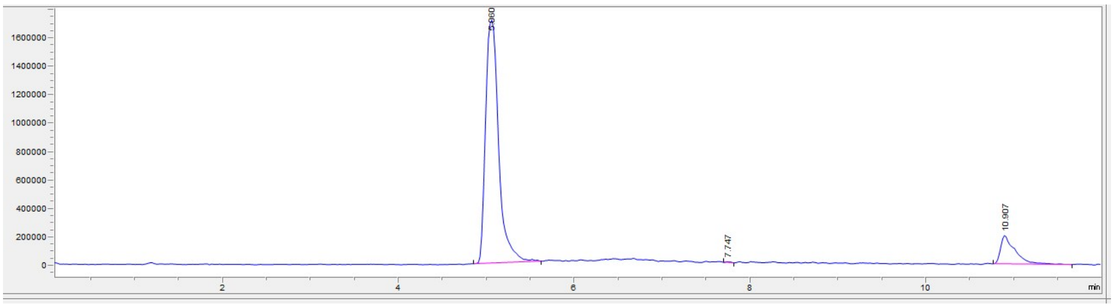

MS Spectrum for **3y**:

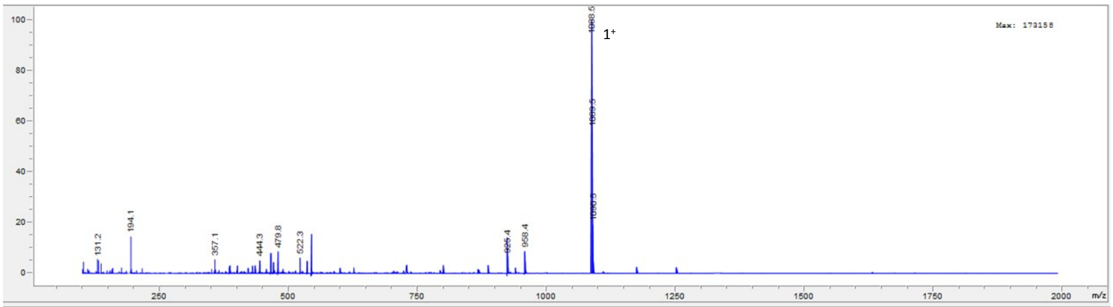

MS MS Spectrum for **3y**:

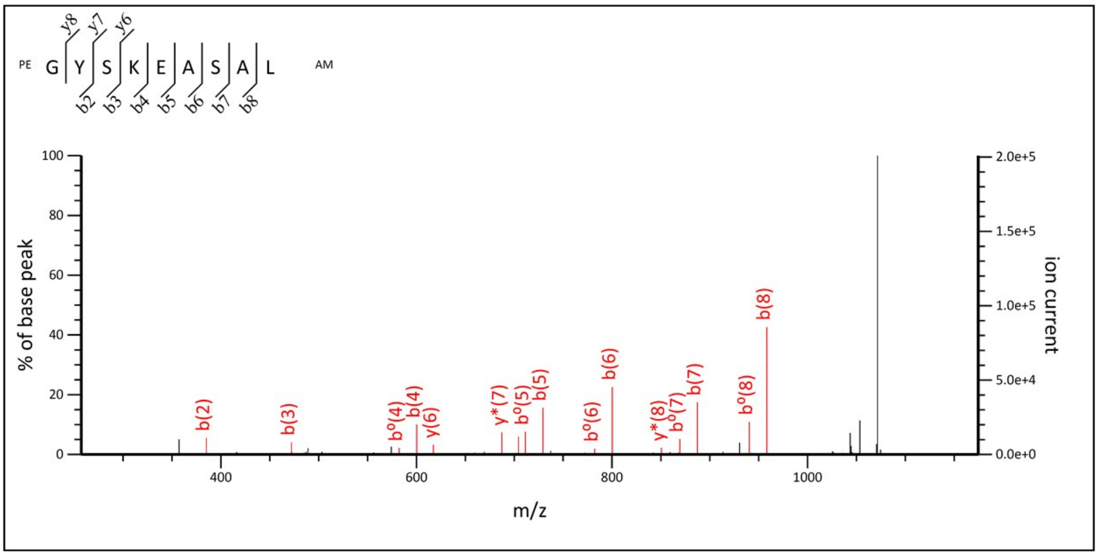

```
Monoisotopic mass of neutral peptide Mr(calc): 1087.5397
Variable modifications:
N-term : PentaHydroxyHexyl (N-term)
C-term : Amidated (Protein C-term)
Ions Score: 79   Expect: 1.2e-008    (help)
```

#	b	b*	b⁰	Seq.	y	y*	y⁰	#
1	222.0972			G				9
2	**385.1605**			Y	**867.4571**	**850.4305**	849.4465	8
3	**472.1926**		454.1820	S	**704.3937**	**687.3672**	686.3832	7
4	**600.2875**	583.2610	**582.2770**	K	**617.3617**	**600.3352**	599.3511	6
5	**729.3301**	712.3036	**711.3196**	E	489.2667		471.2562	5
6	**800.3672**	783.3407	**782.3567**	A	360.2241		342.2136	4
7	**887.3993**	870.3727	**869.3887**	S	289.1870		271.1765	3
8	**958.4364**	941.4098	**940.4258**	A	202.1550			2
9				L	131.1179			1

Trypsin digested MS spectrum for **3y**:

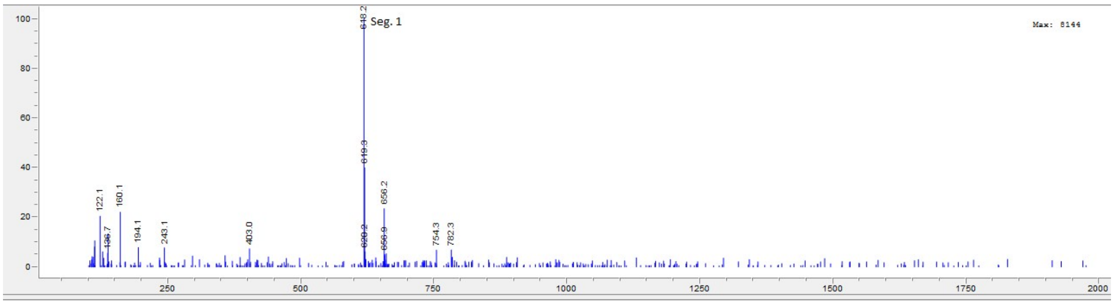

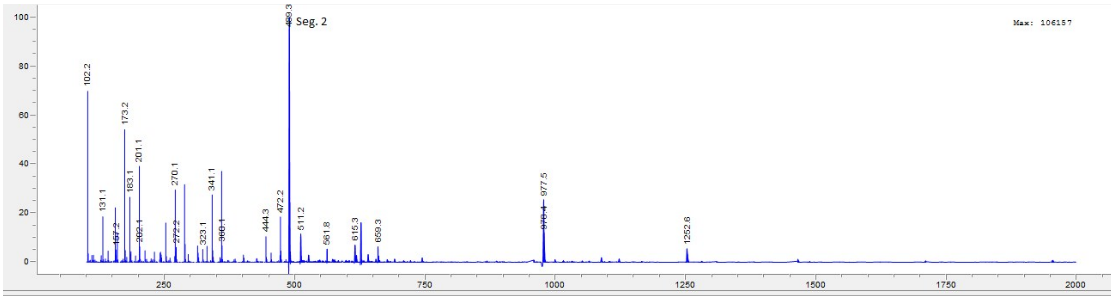

TIC for **3z**:

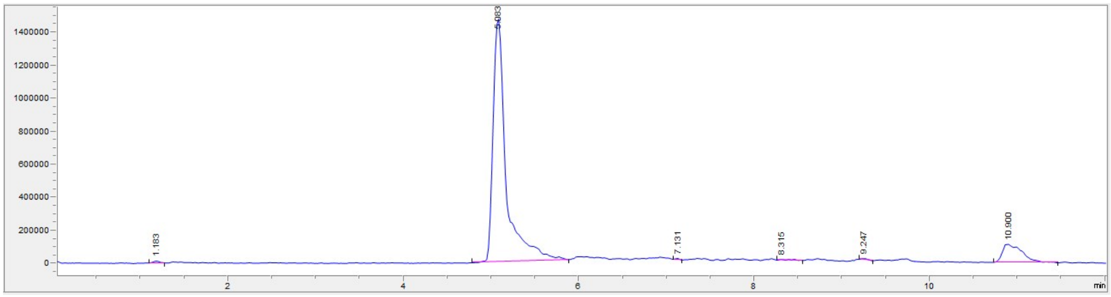

MS Spectrum for **3z**:

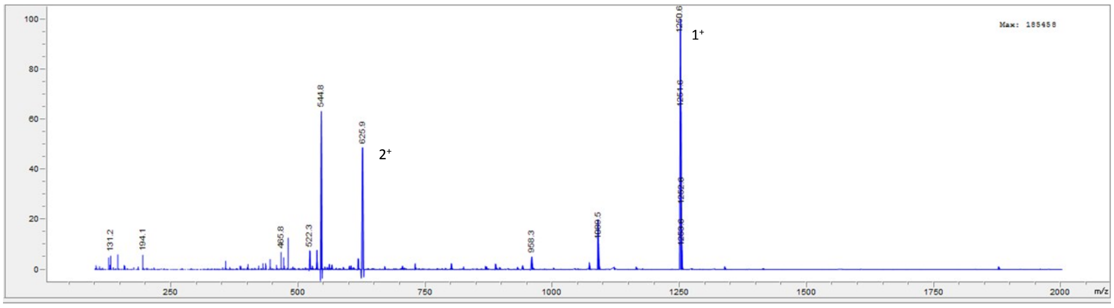

MS MS Spectrum for **3z**:

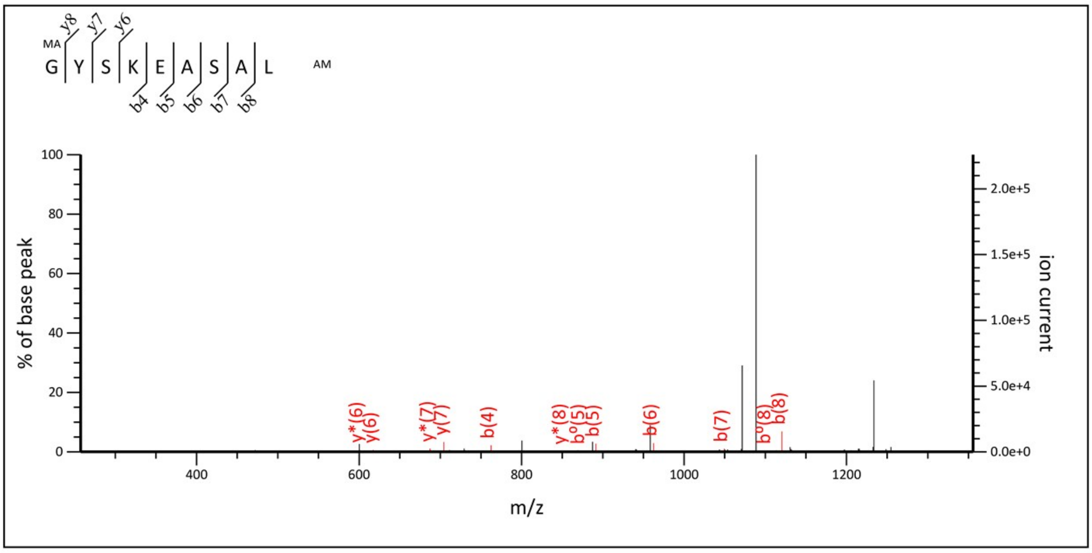

Monoisotopic mass of neutral peptide Mr(calc): 1249.5925
Variable modifications:
G1 : Maltose-yl (G)
C-term : Amidated (C-term)
Ions Score: 22 **Expect:** 0.0065 (help)

#	b	b*	b^0	Seq.	y	y*	y^0	#
1	384.1500			G				9
2	547.2134			Y	867.4571	**850.4305**	849.4465	8
3	634.2454		616.2348	S	**704.3937**	**687.3672**	686.3832	7
4	**762.3404**	745.3138	744.3298	K	**617.3617**	**600.3352**	599.3511	6
5	**891.3830**	874.3564	**873.3724**	E	489.2667		471.2562	5
6	**962.4201**	945.3935	944.4095	A	360.2241		342.2136	4
7	**1049.4521**	1032.4255	1031.4415	S	289.1870		271.1765	3
8	**1120.4892**	1103.4627	**1102.4786**	A	202.1550			2
9				L	131.1179			1

Trypsin digested MS spectrum for **3z**:

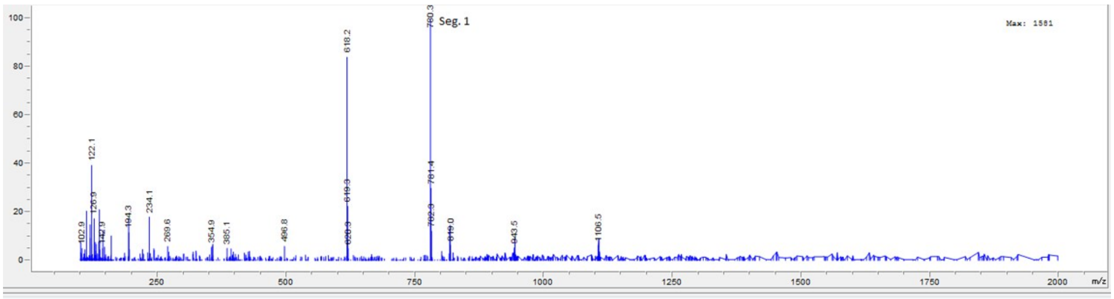
Seg. 1
Max: 1581
m/z

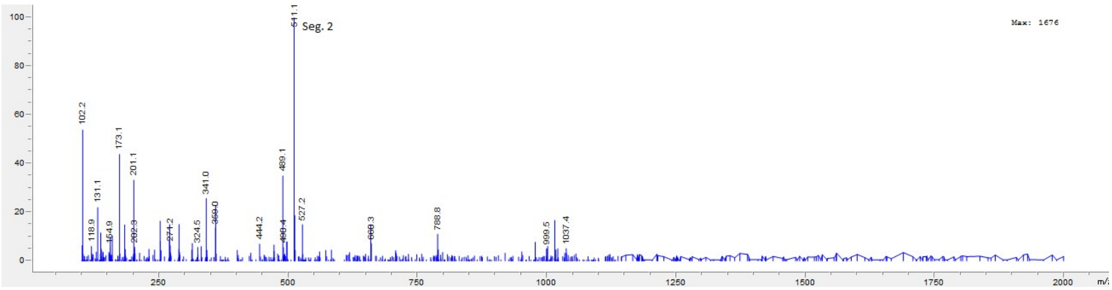
Seg. 2
Max: 1676
m/z

ESI for big protein:

GLP-1:

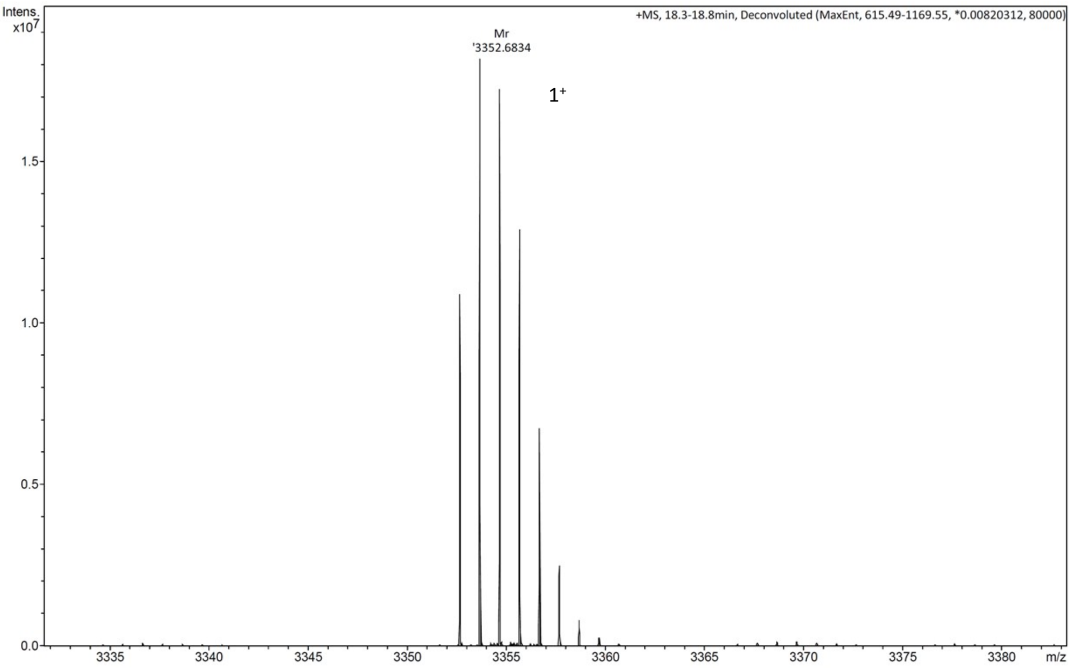

Benzyl_GLP-1 (**4**)

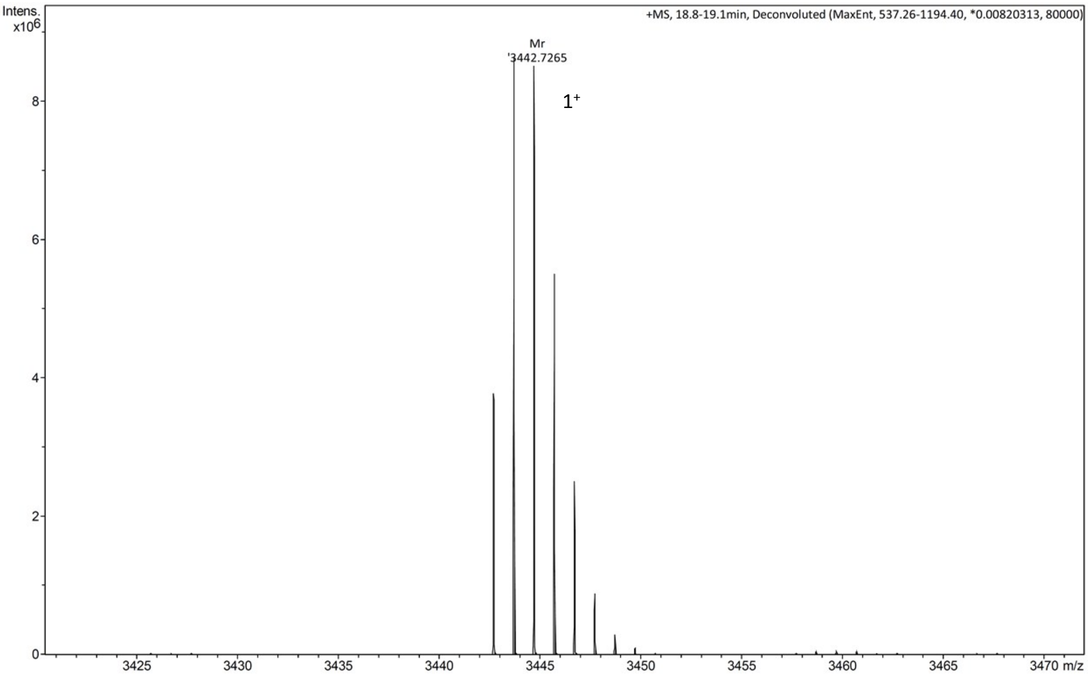

Trypsin digested MS spectrum for **4**:

For Seg.1, sequence: Benzyl-HAEGTFTSDVSSYLEGQAAK (MW: 2188.19)

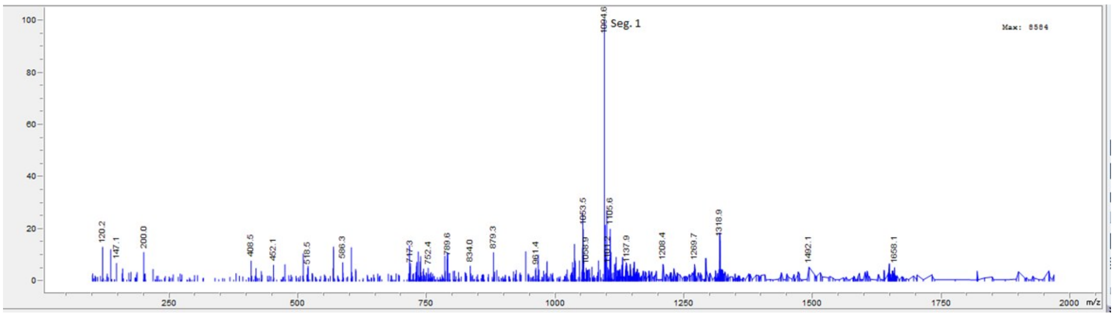

For Seg.2, sequence:EFIAWLVK (MW: 1005.21)

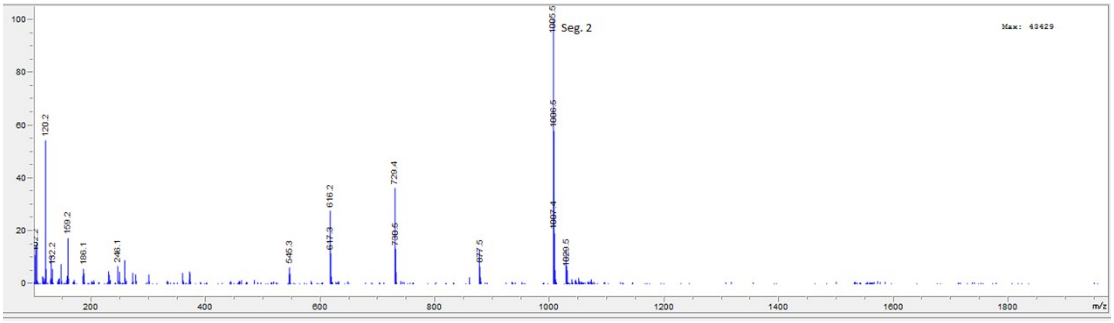

MS MS Spectrum for Seg.1:

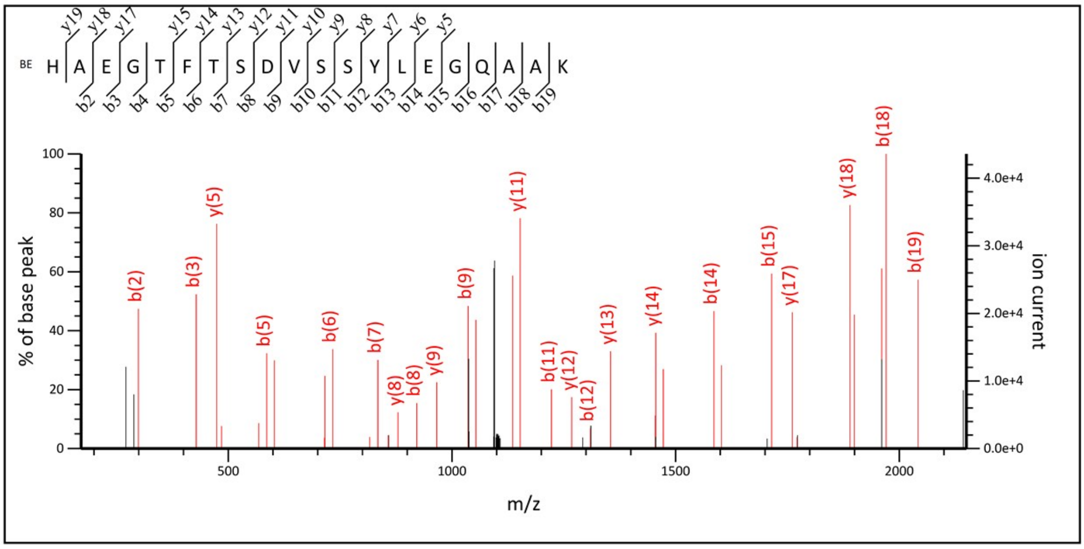

Monoisotopic mass of neutral peptide Mr(calc): 2187.0120
Variable modifications:
N-term : Benzyl (N-term)
Ions Score: 168 **Expect:** 1.5e-017
Matches : 38/182 fragment ions using 53 most intense peaks (help)

#	b	b++	b*	b*++	b0	b0++	Seq.	y	y++	y*	y*++	y0	y0++	#
1	228.1131	114.5602					H							20
2	299.1503	150.0788					A	1960.9134	980.9604	1943.8869	972.4471	1942.9029	971.9551	19
3	428.1928	214.6001			410.1823	205.5948	E	1889.8763	945.4418	1872.8498	936.9285	1871.8658	936.4365	18
4	485.2143	243.1108			467.2037	234.1055	G	1760.8337	880.9205	1743.8072	872.4072	1742.8232	871.9152	17
5	586.2620	293.6346			568.2514	284.6293	T	1703.8123	852.4098	1686.7857	843.8965	1685.8017	843.4045	16
6	733.3304	367.1688			715.3198	358.1636	F	1602.7646	801.8859	1585.7380	793.3727	1584.7540	792.8807	15
7	834.3781	417.6927			816.3675	408.6874	T	1455.6962	728.3517	1438.6696	719.8385	1437.6856	719.3464	14
8	921.4101	461.2087			903.3995	452.2034	S	1354.6485	677.8279	1337.6220	669.3146	1336.6379	668.8226	13
9	1036.4371	518.7222			1018.4265	509.7169	D	1267.6165	634.3119	1250.5899	625.7986	1249.6059	625.3066	12
10	1135.5055	568.2564			1117.4949	559.2511	V	1152.5895	576.7984	1135.5630	568.2851	1134.5790	567.7931	11
11	1222.5375	611.7724			1204.5269	602.7671	S	1053.5211	527.2642	1036.4946	518.7509	1035.5106	518.2589	10
12	1309.5695	655.2884			1291.5590	646.2831	S	966.4891	483.7482	949.4625	475.2349	948.4785	474.7429	9
13	1472.6329	736.8201			1454.6223	727.8148	Y	879.4571	440.2322	862.4305	431.7189	861.4465	431.2269	8
14	1585.7169	793.3621			1567.7064	784.3568	L	716.3937	358.7005	699.3672	350.1872	698.3832	349.6952	7
15	1714.7595	857.8834			1696.7489	848.8781	E	603.3097	302.1585	586.2831	293.6452	585.2991	293.1532	6
16	1771.7810	886.3941			1753.7704	877.3888	G	474.2671	237.6372	457.2405	229.1239			5

MS MS Spectrum for Seg.2:

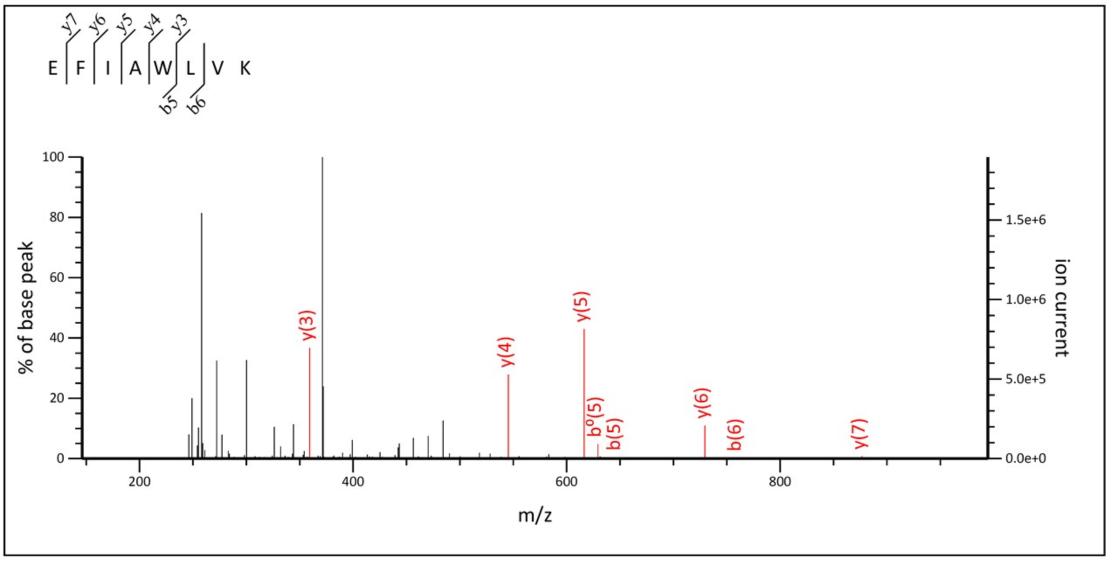

Label all possible matches ○ Label matches used for scoring ⦿

Monoisotopic mass of neutral peptide Mr(calc): 1004.5695
Ions Score: 31 **Expect:** 0.00081
Matches : 8/56 fragment ions using 13 most intense peaks (help)

#	b	b++	b0	b0++	Seq.	y	y++	y*	y*++	#
1	130.0499	65.5286	112.0393	56.5233	E					8
2	277.1183	139.0628	259.1077	130.0575	F	876.5342	438.7707	859.5076	430.2575	7
3	390.2023	195.6048	372.1918	186.5995	I	729.4658	365.2365	712.4392	356.7233	6
4	461.2395	231.1234	443.2289	222.1181	A	616.3817	308.6945	599.3552	300.1812	5
5	647.3188	324.1630	629.3082	315.1577	W	545.3446	273.1759	528.3180	264.6627	4
6	760.4028	380.7051	742.3923	371.6998	L	359.2653	180.1363	342.2387	171.6230	3
7	859.4713	430.2393	841.4607	421.2340	V	246.1812	123.5942	229.1547	115.0810	2
8					K	147.1128	74.0600	130.0863	65.5468	1

RNase A:

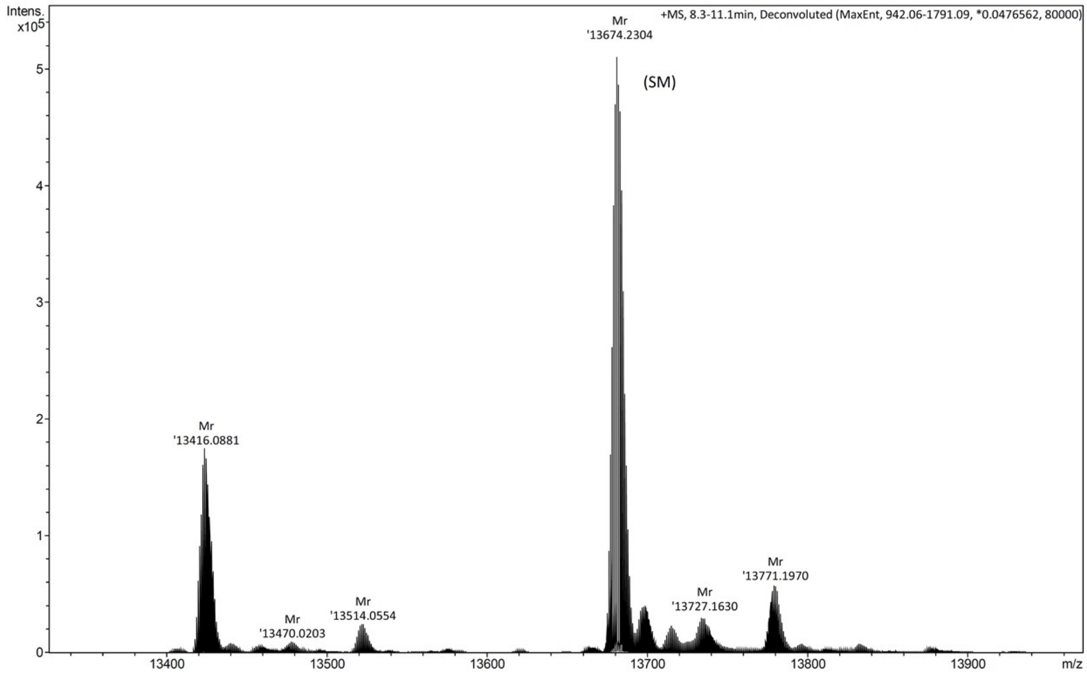

Benzyl_RNase A (**5**):

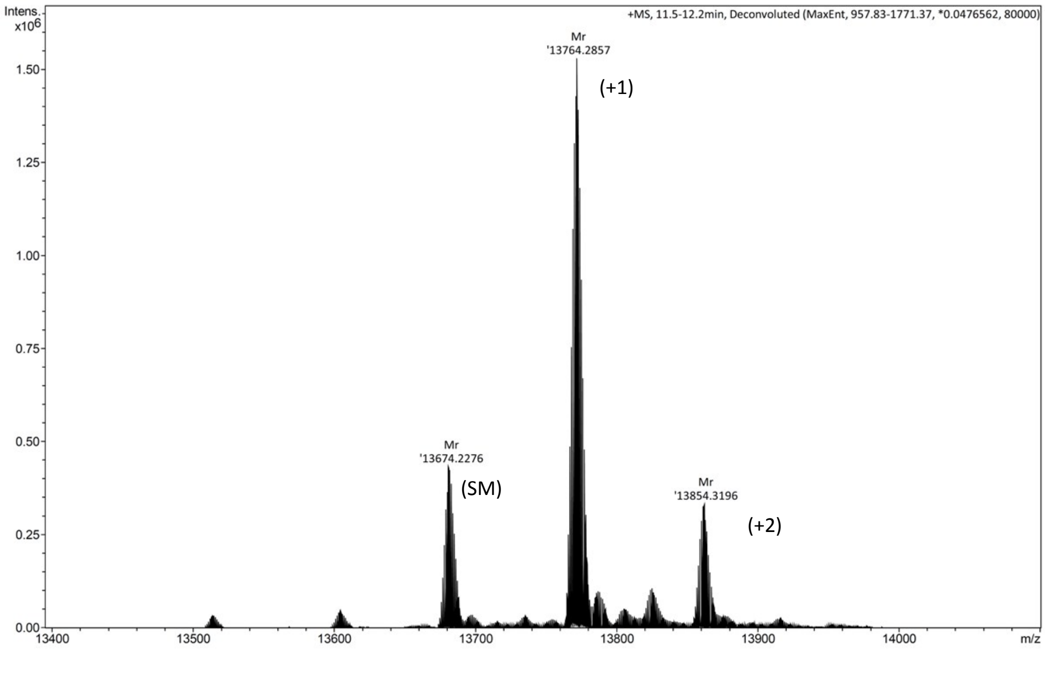

Trypsin digested MS spectrum for **5**:

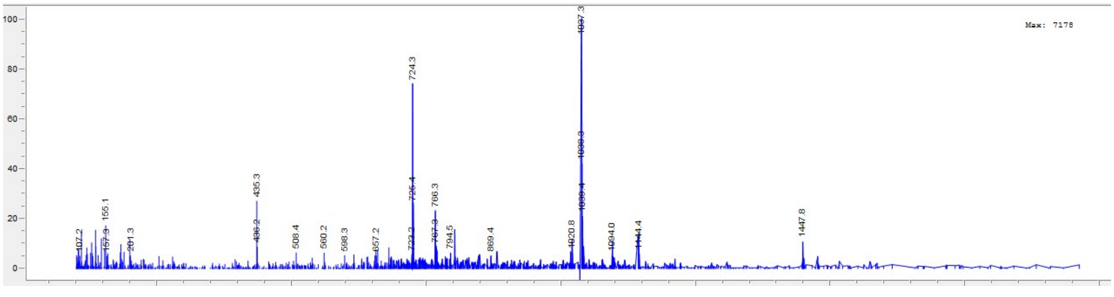

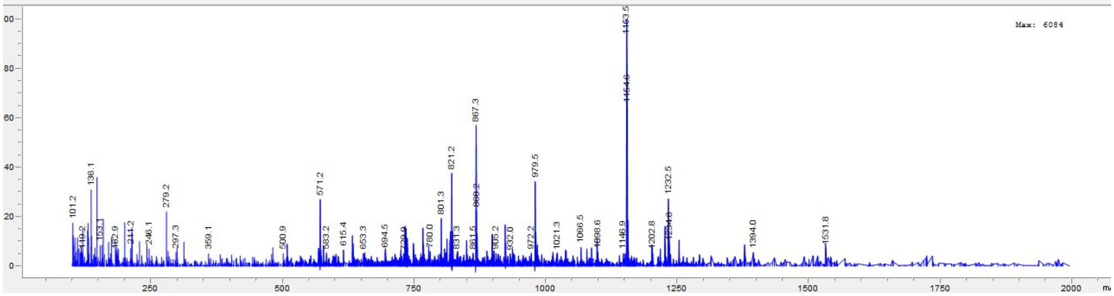

Lysozyme:

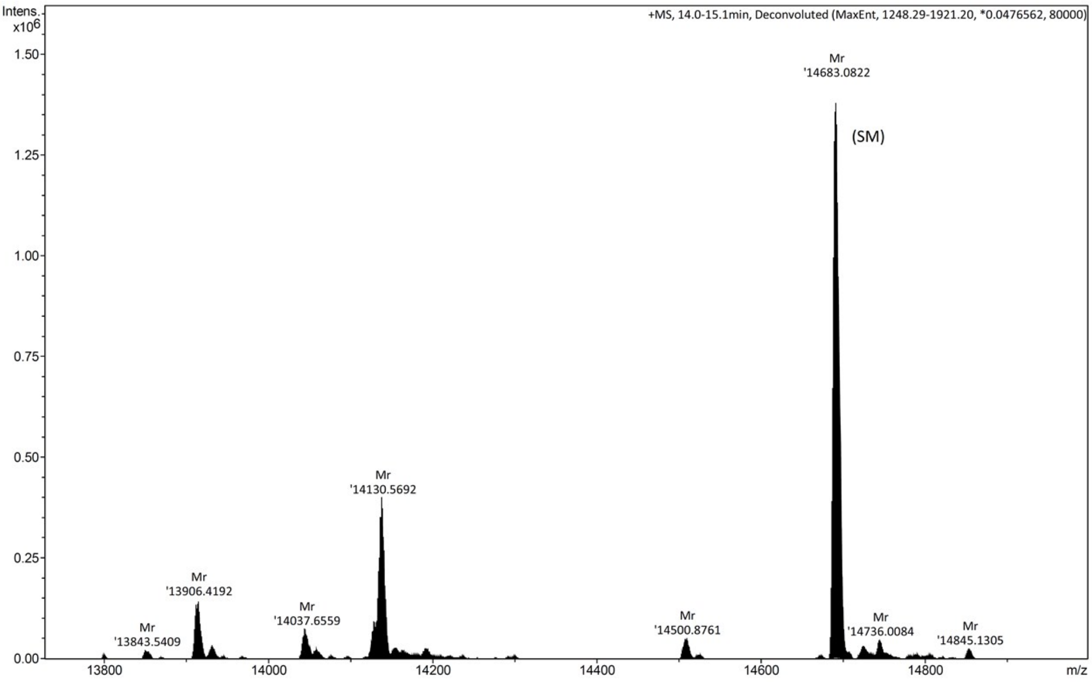

Benzyl_Lysozyme (**6**):

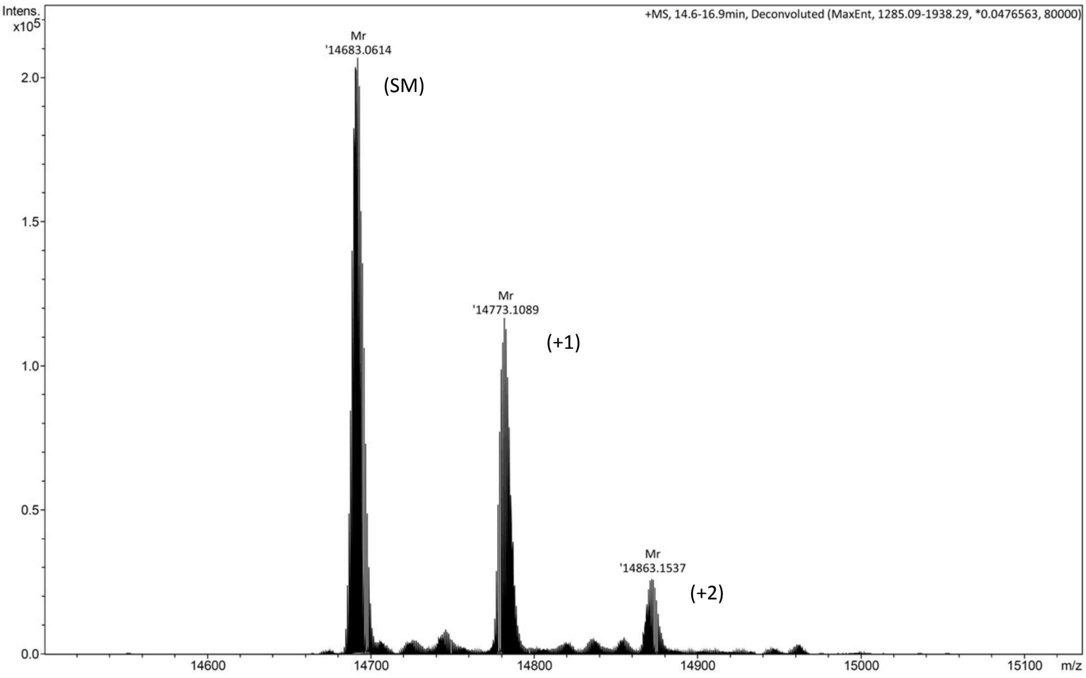

Trypsin digested MS spectrum for **6**:

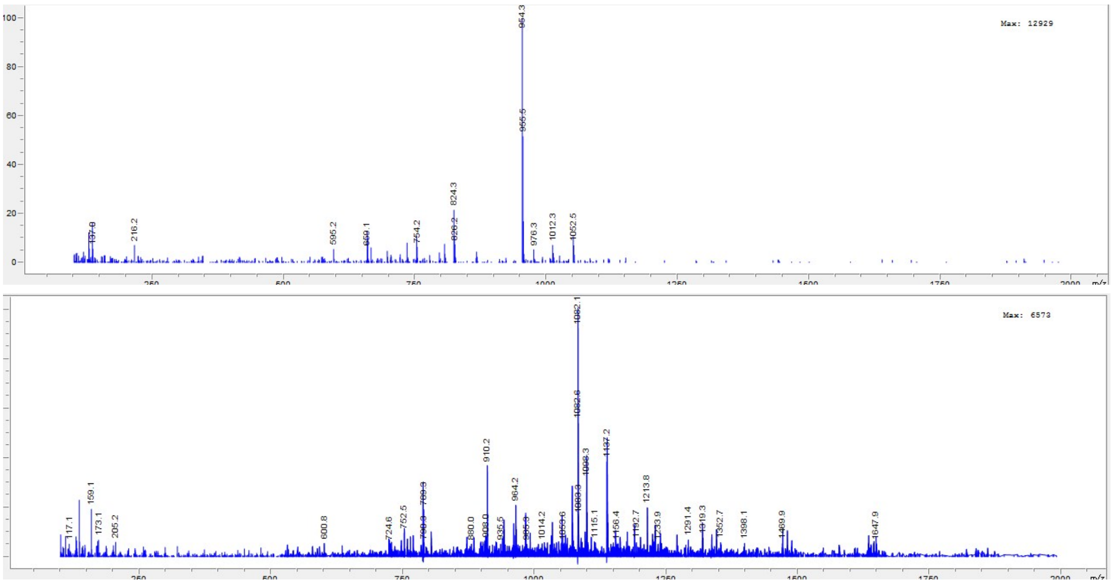

TIC for B1_Benzyl_Insulin (**7**):

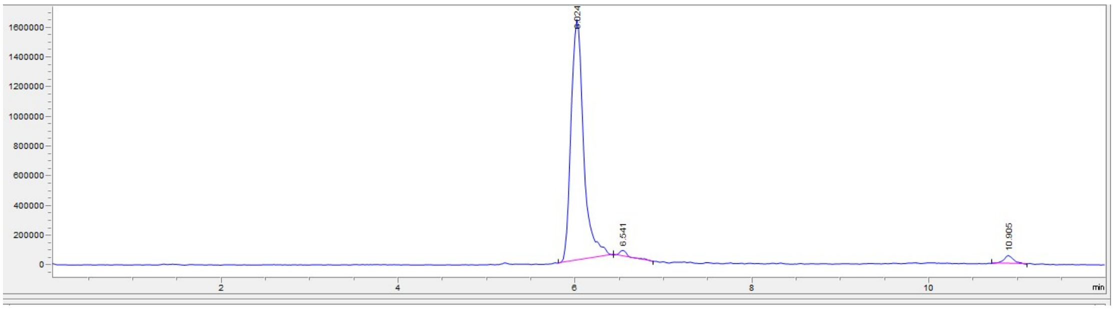

MS Spectrum for **7** (MW 5898.5):

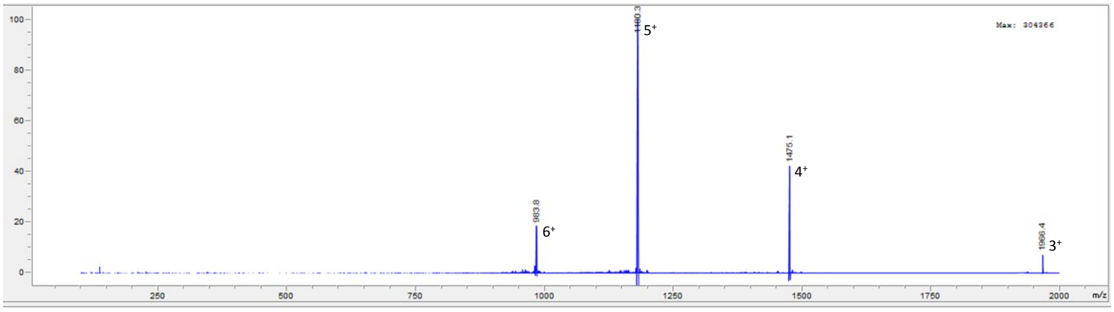

After DTT Analysis of **7**, In A chain (MW 2383.7)

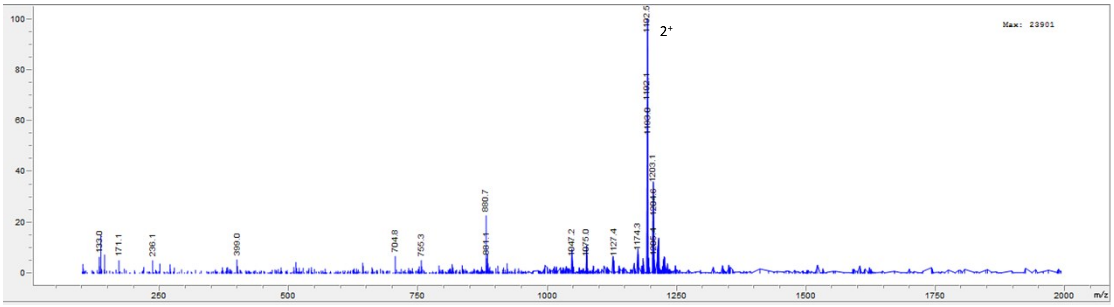

After DTT Analysis of **7**, In B_benzyl (MW 3519.9)

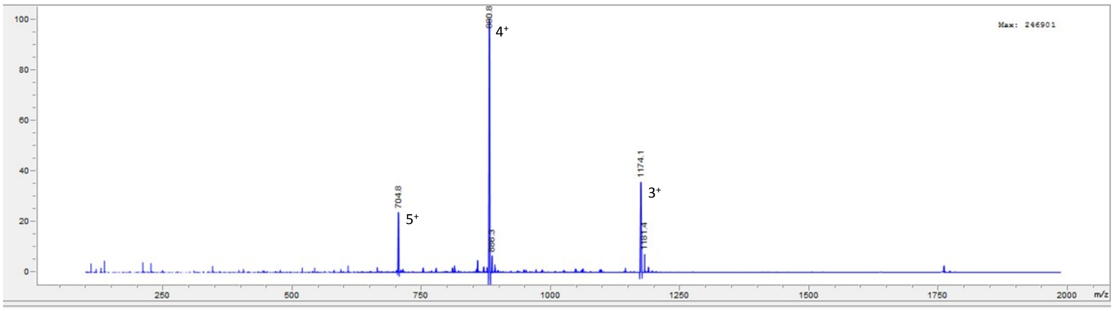

MS MS Spectrum for InB_benzyl:

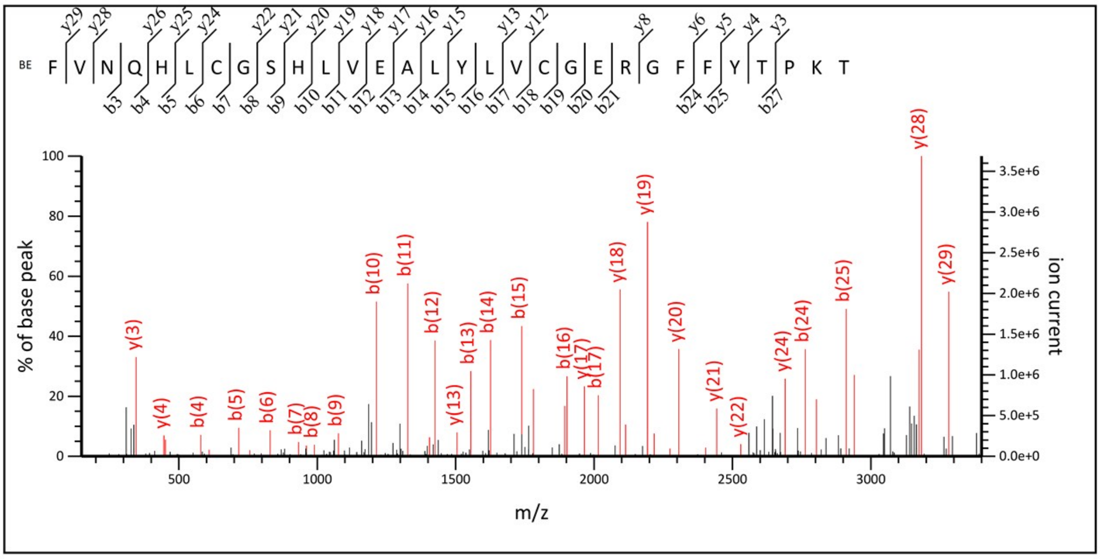

Monoisotopic mass of neutral peptide Mr(calc): 3517.7315
Variable modifications:
N-term : Benzyl (N-term)
Ions Score: 185 **Expect:** 3.5e-019
Matches : 42/326 fragment ions using 66 most intense peaks (help)

#	b	b^{++}	b*	b^{*++}	b^0	b^{0++}	Seq.	y	y^{++}	y*	y^{*++}	y^0	y^{0++}	#
1	238.1226	119.5650					F							30
2	337.1911	169.0992					V	***3281.6234***	1641.3154	3264.5969	1632.8021	3263.6129	1632.3101	29
3	***451.2340***	226.1206	434.2074	217.6074			N	***3182.5550***	1591.7812	3165.5285	1583.2679	3164.5445	1582.7759	28
4	***579.2926***	290.1499	562.2660	281.6366			Q	3068.5121	1534.7597	3051.4855	1526.2464	3050.5015	1525.7544	27
5	***716.3515***	358.6794	699.3249	350.1661			H	***2940.4535***	1470.7304	2923.4270	1462.2171	2922.4430	1461.7251	26
6	***829.4355***	415.2214	812.4090	406.7081			L	***2803.3946***	1402.2009	2786.3681	1393.6877	2785.3840	1393.1957	25
7	***932.4447***	466.7260	915.4182	458.2127			C	***2690.3105***	1345.6589	2673.2840	1337.1456	2672.3000	1336.6536	24
8	***989.4662***	495.2367	972.4396	486.7235			G	2587.3014	1294.1543	2570.2748	1285.6410	2569.2908	1285.1490	23
9	***1076.4982***	538.7527	1059.4717	530.2395	1058.4876	529.7475	S	***2530.2799***	1265.6436	2513.2533	1257.1303	2512.2693	1256.6383	22
10	***1213.5571***	607.2822	1196.5306	598.7689	1195.5466	598.2769	H	***2443.2479***	1222.1276	2426.2213	1213.6143	2425.2373	1213.1223	21
11	***1326.6412***	663.8242	1309.6146	655.3110	1308.6306	654.8189	L	***2306.1890***	1153.5981	2289.1624	1145.0848	2288.1784	1144.5928	20
12	***1425.7096***	713.3584	1408.6831	704.8452	1407.6990	704.3532	V	***2193.1049***	1097.0561	2176.0783	1088.5428	2175.0943	1088.0508	19
13	***1554.7522***	777.8797	1537.7256	769.3665	1536.7416	768.8745	E	***2094.0365***	1047.5219	2077.0099	1039.0086	2076.0259	1038.5166	18
14	***1625.7893***	813.3983	1608.7628	804.8850	1607.7787	804.3930	A	***1964.9939***	983.0006	1947.9673	974.4873	1946.9833	973.9953	17
15	***1738.8734***	869.9403	1721.8468	861.4271	1720.8628	860.9350	L	***1893.9568***	947.4820	1876.9302	938.9687	1875.9462	938.4767	16
16	***1901.9367***	951.4720	1884.9102	942.9587	1883.9261	942.4667	Y	***1780.8727***	890.9400	1763.8462	882.4267	1762.8621	881.9347	15
17	***2015.0208***	1008.0140	1997.9942	999.5007	1997.0102	999.0087	L	1617.8094	809.4083	1600.7828	800.8951	1599.7988	800.4030	14
18	***2114.0892***	1057.5482	2097.0626	1049.0350	2096.0786	1048.5429	V	***1504.7253***	752.8663	1487.6988	744.3530	1486.7147	743.8610	13
19	***2217.0984***	1109.0528	2200.0718	1100.5395	2199.0878	1100.0475	C	***1405.6569***	703.3321	1388.6304	694.8188	1387.6463	694.3268	12

Aldolase:

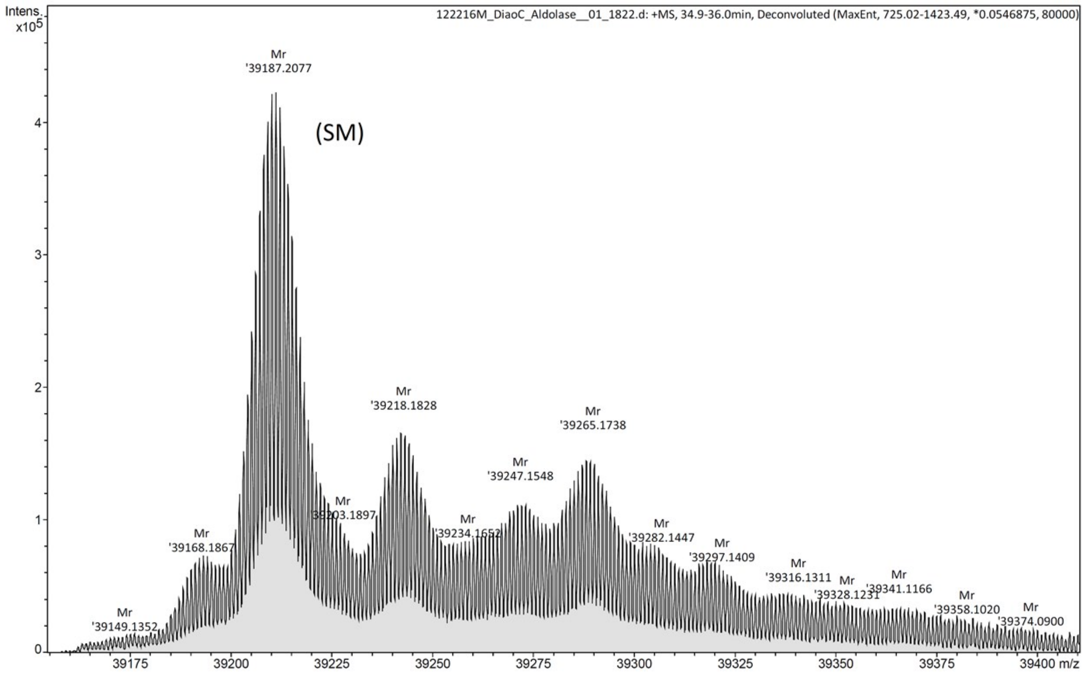

Benzyl_ aldolase (**8**):

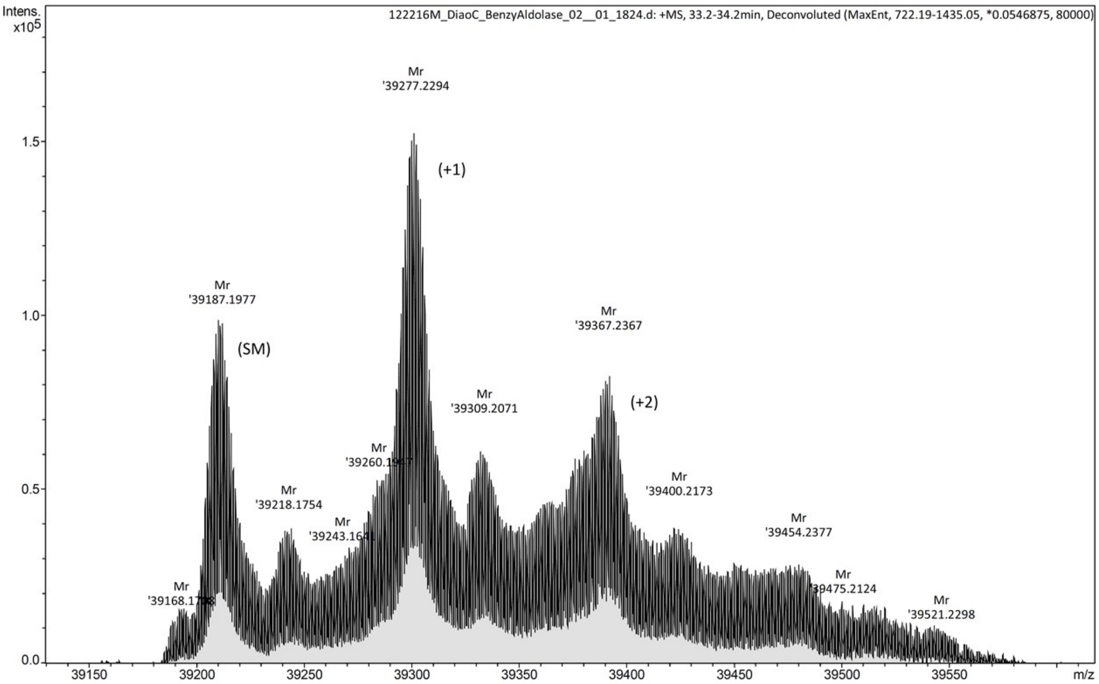

Trypsin digested MS spectrum for **8**:

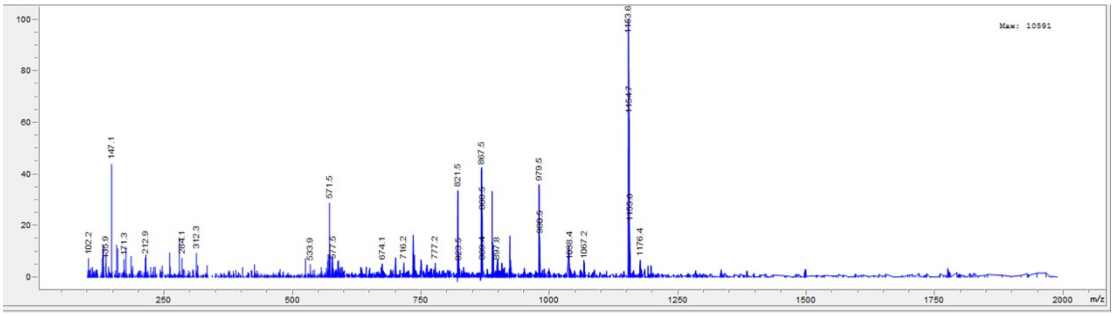

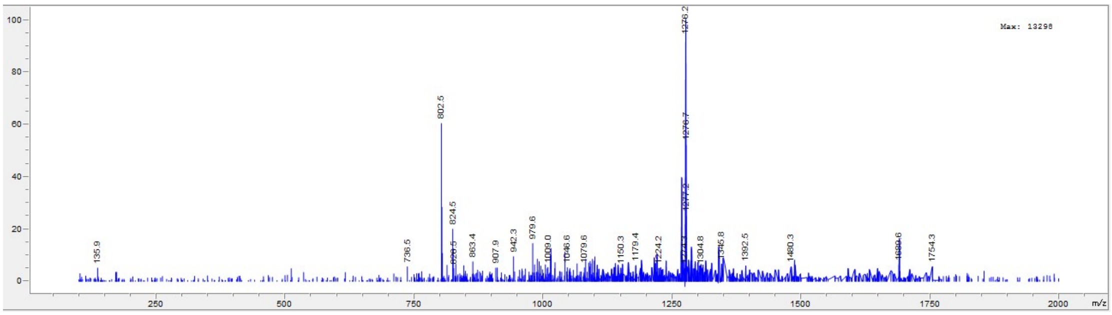

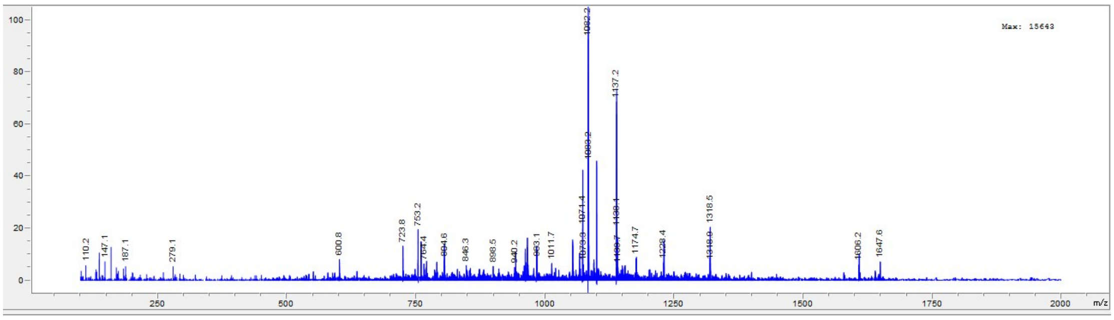

Creatine phosphokinase:

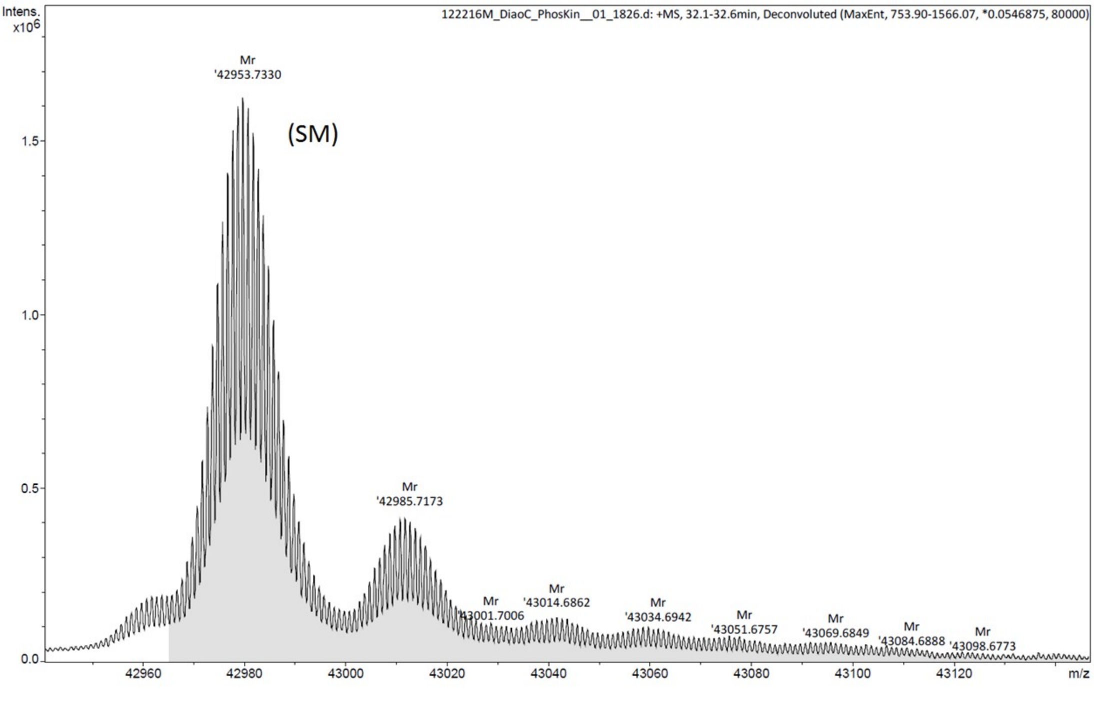

Benzyl_ Creatine phosphokinase (**9**):

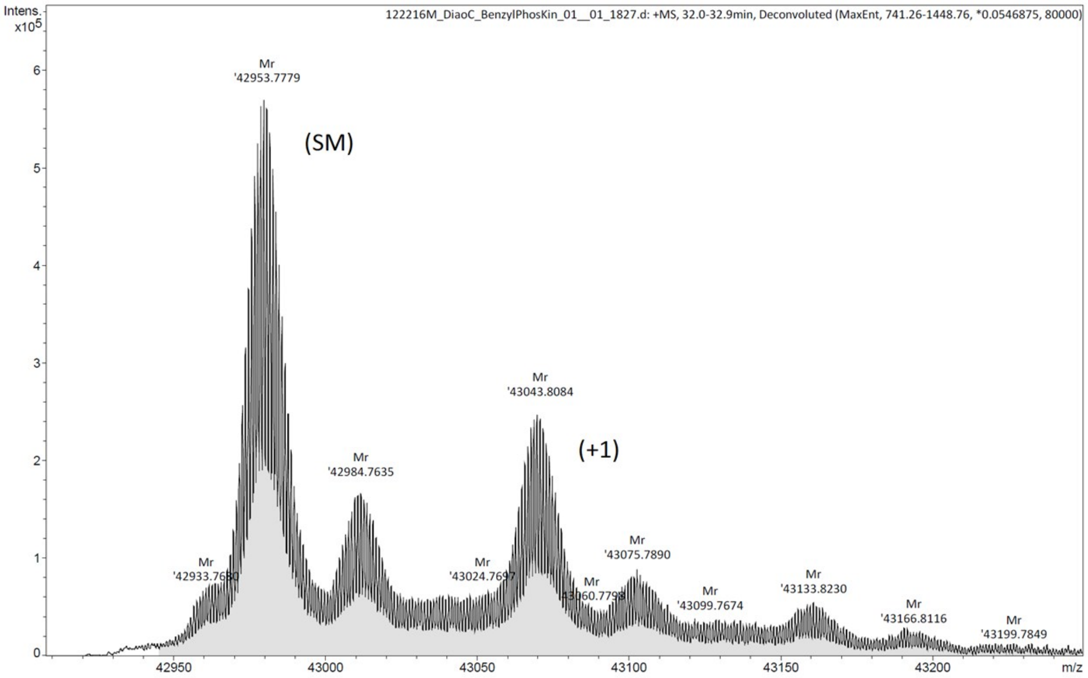

Trypsin digested MS spectrum for **9**:

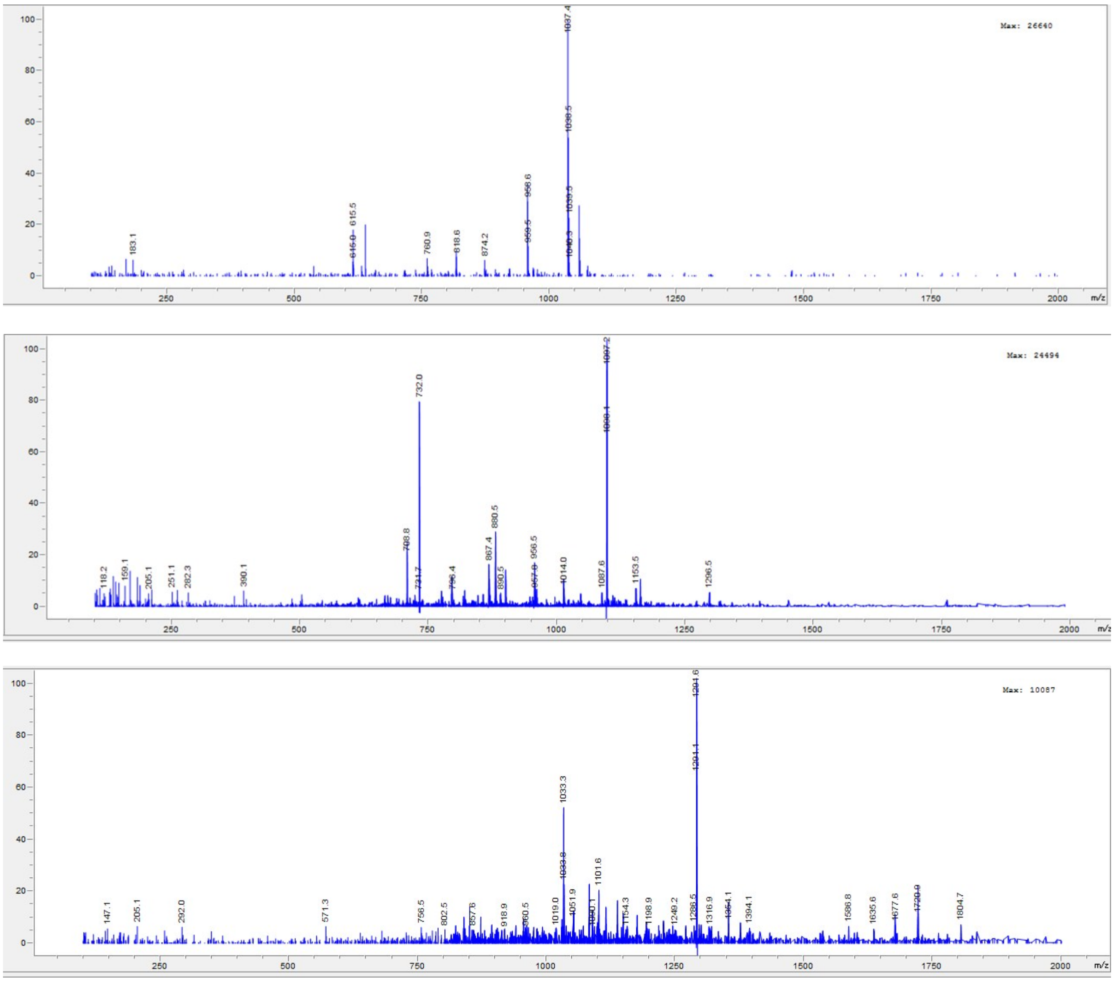

TIC for Biotin_GLP-1 (**11**):

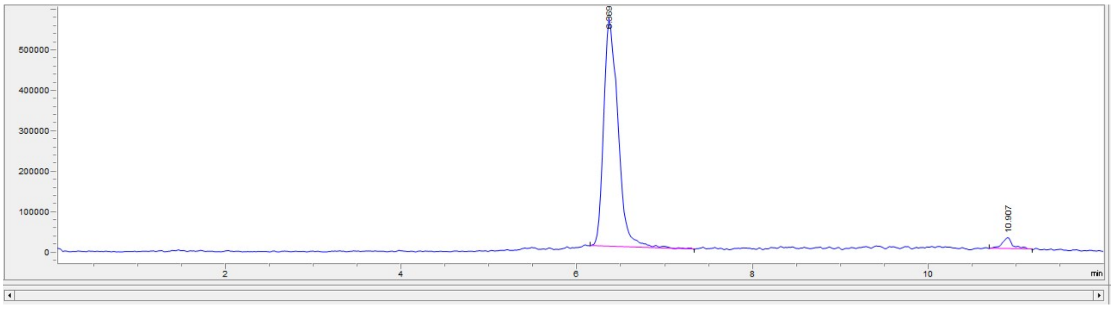

MS Spectrum for **11** (MW 3868.6):

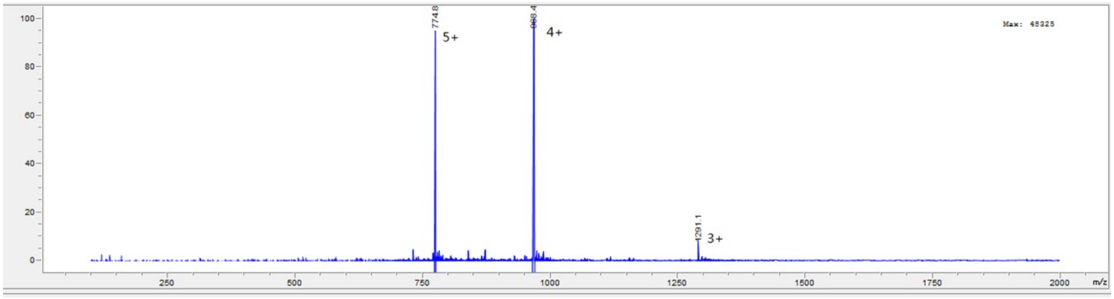

TIC for B1_FITC_Insulin (**14**):

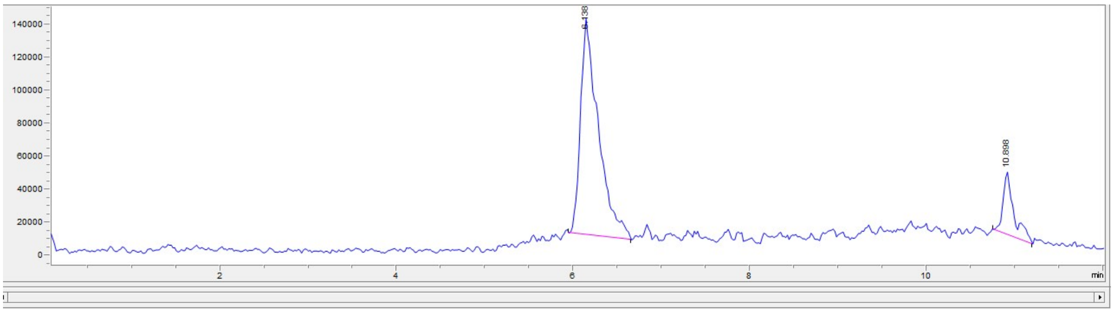

MS Spectrum for **14** (MW 6411.6):

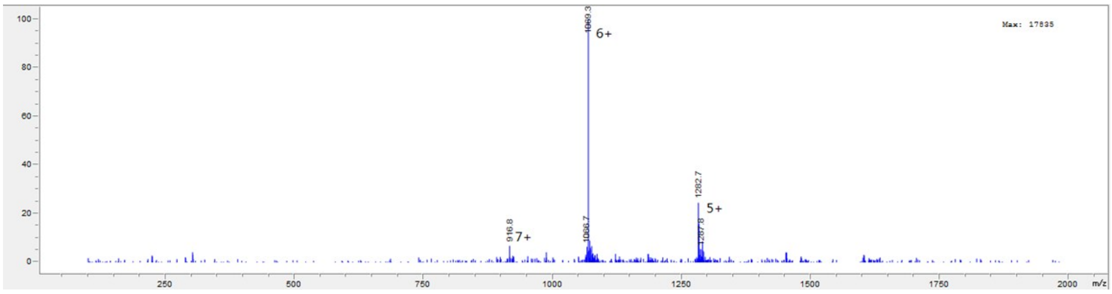

Dtt analysis for **14**:

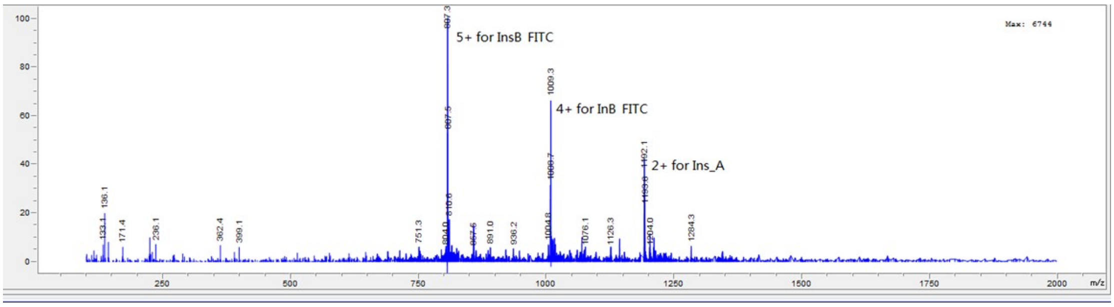

TIC for A1_Benzyl_Insulin (**15**):

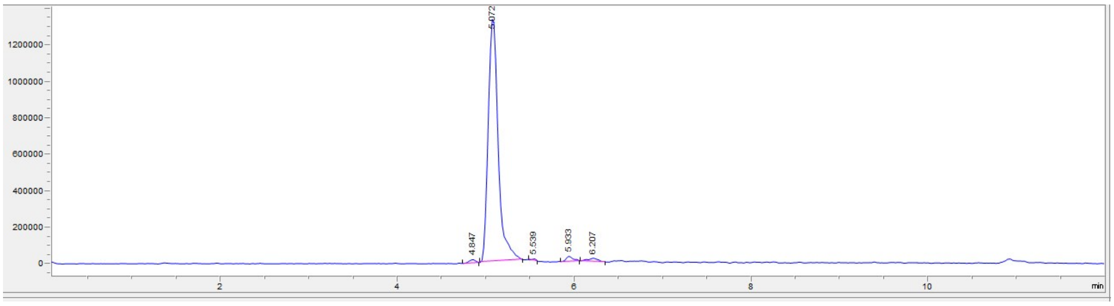

MS Spectrum for **15** (MW 5898.5):

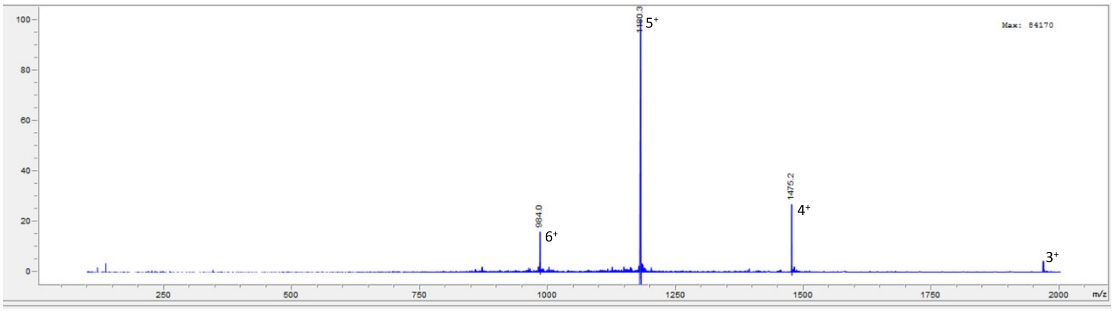

After DTT Analysis of **15**, In A_benzyl (MW 2473.7)

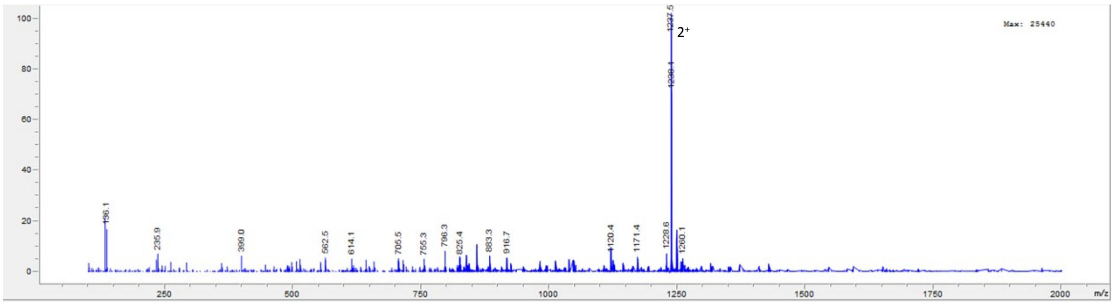

After DTT Analysis of **15**, In B chain (MW 3429.9)

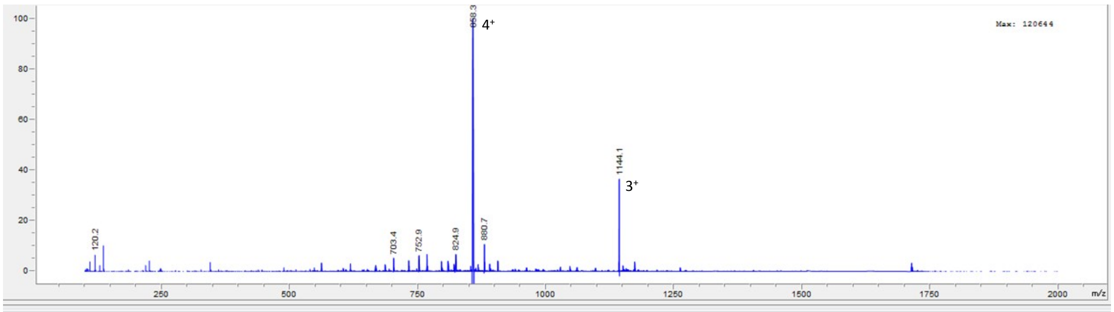

A chain_Benzoyl_Insulin (**16**):

TIC for **16**:

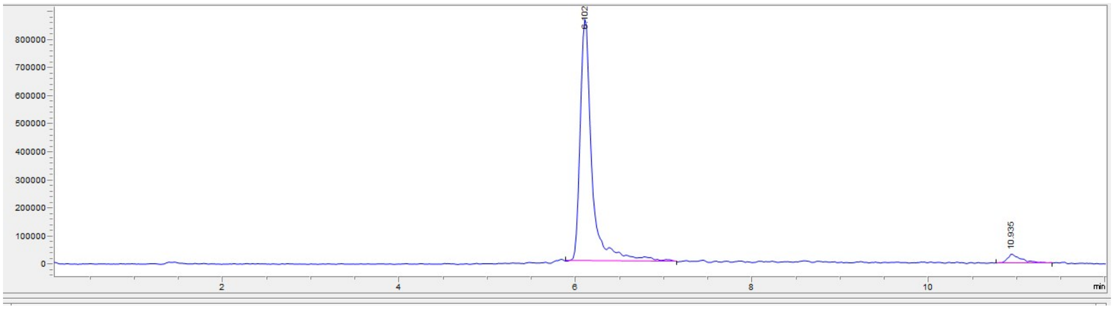

MS Spectrum for **16** (MW 5912.5):

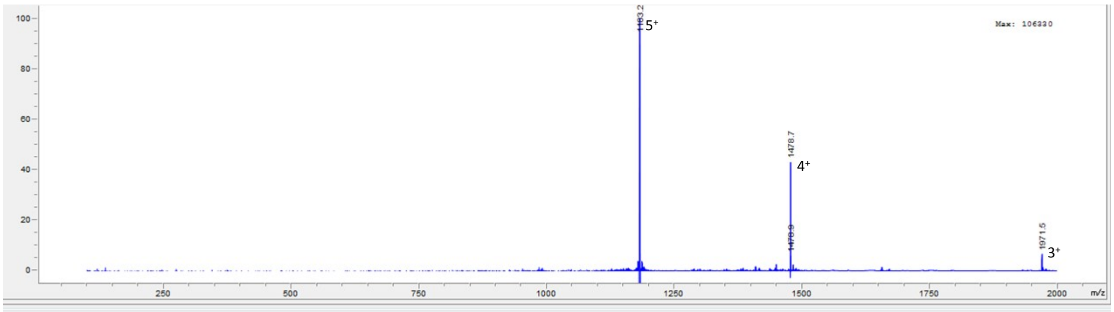

After DTT Analysis of **16**, In A_benzoyl (MW 2487.7)

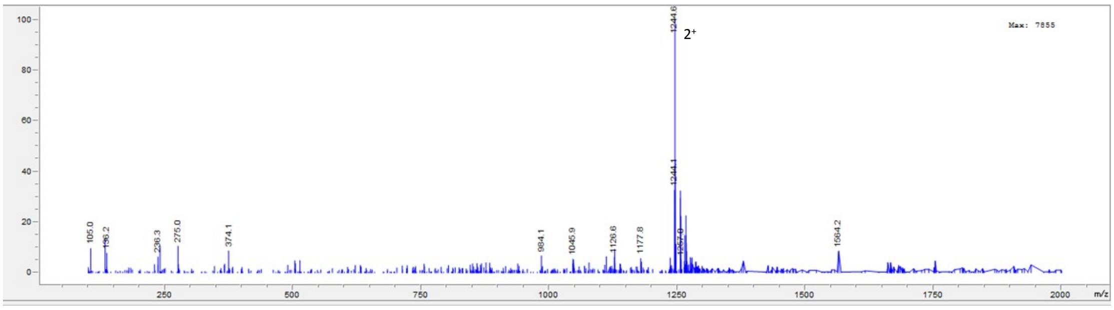

After DTT Analysis of **16**, In B (MW 3429.9)

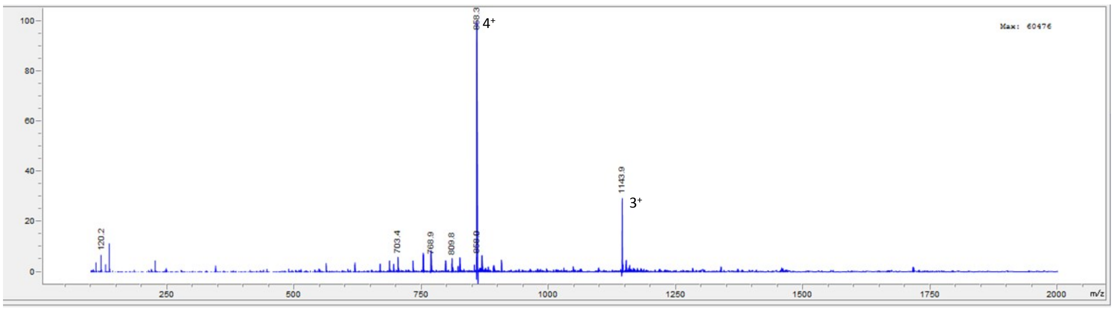

Supplement: Supplementary file 1 [file SC-008-C6SC04744K-s001.pdf]
